# Supplementary material for: Neoadjuvant tislelizumab plus stereotactic body radiotherapy and adjuvant tislelizumab in early-stage resectable hepatocellular carcinoma: the Notable-HCC phase 1b trial
Source: Nat Commun. 2024 Apr 16;15:3260. doi: 10.1038/s41467-024-47420-3 (PMC11021407; doi:10.1038/s41467-024-47420-3)
Supplement: Supplementary file 1 — Supplementary Information [file 41467_2024_47420_MOESM1_ESM.pdf]

Supplementary results

Safety analysis of the curative HCC resection after the neoadjuvant therapy

In order to validate whether the neoadjuvant therapy of tislelizumab plus SBRT increase the surgical difficulties and the risk of complications in the following HCC resection, we retrospectively analyzed a cohort of 203 HCC patients who underwent upfront curative resection in our center during the same period as the trial was ongoing.

Results

The baseline demographics, surgical characteristics, and post-resection features of the 19 patients recruited in the trial were compared to the upfront resection cohort. The two groups showed no significant difference in the distributions of baseline age, gender and BCLC stage. The two groups showed no significant difference in the mean operation time (135.8±54.2 vs. 159.7±51.9, P=0.058; min, mean ± SD), blood loss (68.9±47.7 vs. 83.3±58.0, P=0.296; ml, mean ± SD), abdominal drainage time (4.3±2.6 vs. 5.7±4.0, P=0.070; days, mean ± SD) and post-operative hospital stay (16.4±6.7 vs. 15.4±5.3, P=0.439; days, mean ± SD). In patients after the neoadjuvant

therapy, post-operative increase of alanine aminotransferase was significantly lower than the patients of upfront resection (178.74 [106.25~679.19] vs. 502.45 [198.83~1030.43], P=0.019; %, median [IQR]), aspartate aminotransferase increase was of borderline significance (253.55 [135.96~828.43] vs. 519.68 [253.69~924.57], P=0.051; %, median [IQR]); changes of red blood cells, hemoglobin, bilirubin and albumin were not significantly different compared with the patients who underwent upfront surgery. Generally, the incidences of postoperative complications of the patients after the neoadjuvant therapy were also similar with the patients who underwent upfront surgery (P=0.497). The three most frequent types of the postoperative complications were same in two groups, which were hydrothorax, hypoproteinemia and massive ascites, respectively. No perioperative mortality occurred, and no complications requiring reoperation occurred, either.

Discussion

The combination neoadjuvant therapy did not increase the difficulty of surgical resection, nor did it increase the incidence of post-operative complications, as shown by the peri-operative variables, compared to the data from the patients who were not

recruited and underwent upfront resection during the same period of time in our center as the trial was ongoing.

In patients after the neoadjuvant therapy, resection-induced transient liver function injury was milder, as indicated by the increasing extent of aminotransferase. This could be explained by the possible smaller average tumor size and number in the neoadjuvant therapy patients. Large or multiple tumors are technically challenging for SBRT; so, in our study, the tumor size in 90% participants were less than 5 cm, and the hepatectomy for a smaller tumor normally costs less loss of liver parenchyma (Source Data 1).

Supplementary figure 1

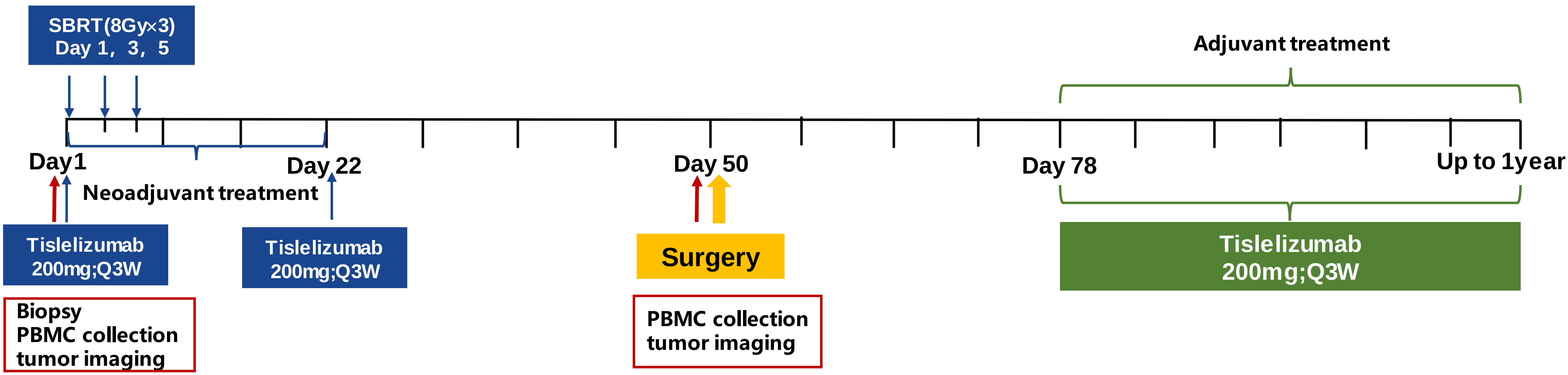

Supplementary figure 1. Trial design. SBRT, stereotactic body radiation therapy; PBMC, peripheral blood mononuclear cells.

Supplementary figure 2

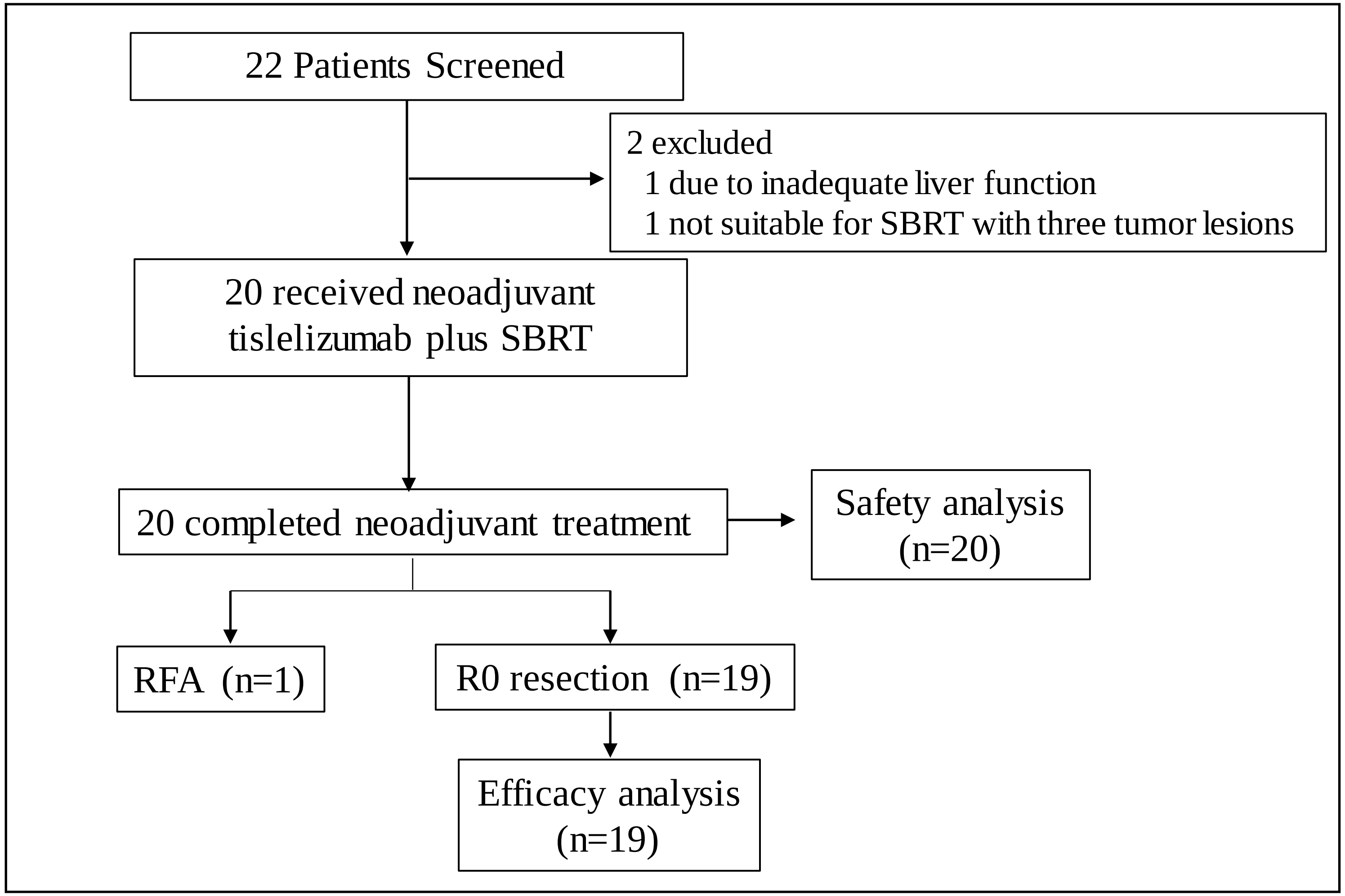

Supplementary figure 2. Patient flow. SBRT, stereotactic body radiotherapy; RFA, radiofrequency ablation; R0, complete surgical resection.

Supplementary figure 3

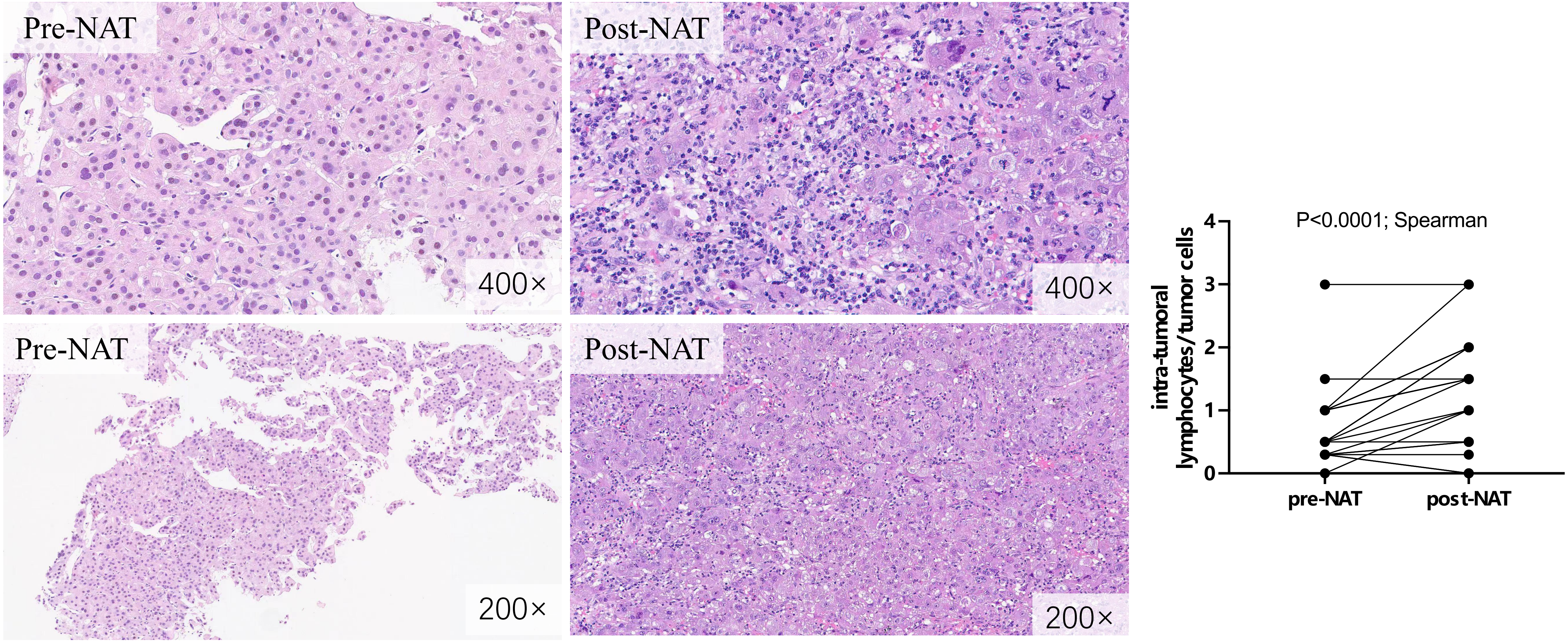

Supplementary figure 3. Intra-tumoral lymphocyte infiltration in the paired pre- and post- neoadjuvant therapy samples from the same patient (n=20). Ratios of infiltrated lymphocytes to tumor cells were calculated by pathological examination and H&E staining, from the paired pre-neoadjuvant biopsy samples and the resected specimen samples from the same patients. Sample images were from patient no. 6; paired non-parametric Spearman analysis was employed to determine the significance of the difference.Source data are provided as a Source Data file.

Supplementary table 1. Baseline characteristics

Characteristics	Patients (n=20)
**Median age, years (range)**	58.5 (48~78)
**Age, years, n (%)**	
＜65	12 (80.0%)
≥65	8 (40.0%)
**Sex, n (%)**	
Female	5 (25.0%)
Male	15 (75.0%)
**Recurrent HCC, n (%)**	
Yes	4 (20.0%)
No	16 (80.0%)
**ECOG PS, n (%)**	
0	19 (95.0%)
1	1 (5.0%)
**Child-Pugh classification, n (%)**	

A5	20 (100.0%)
A6 or above	0 (0%)
**Etiology of HCC, n (%)**	
HBV infection	17 (85.0%)
HBsAg+	16 (80.0%)
HBcAb+	17 (85.0%)
HCV infection	2 (10.0%)
Non-viral	1 (5.0%)
**Serum AFP level, n (%)**	
＜400ng/ml	16 (80.0%)
≥ 400ng/ml	4 (20.0%)
**Tumor number, n (%)**	
Solitary	19 (95.0%)
Multiple	1 (5.0%)
**Tumor size, n (%)**	
＜5cm	18 (90.0%)

≥ 5cm	2 (10.0%)
**Child-Pugh classification, n (%)**	
A5	20 (100.0%)
A6 or above	0 (0%)
**BCLC staging, n (%)**	
0	3 (15%)
A	17 (85.0%)
**CNLC staging, n (%)**	
Ia	17 (85.0%)
Ib	3 (15.0%)

ECOG PS, Eastern Cooperative Oncology Group Performance Status; HBV, hepatitis B virus; HCV, hepatitis C virus; AFP, alpha-fetoprotein; BCLC, Barcelona Clinic Liver Cancer; CNLC, China Liver Cancer Staging.

Supplementary table 2. Details of adverse events of myelosuppression, ALT and AST during neoadjuvant treatment (n=20).

CTCAE terms	grade			deteriorating grade than baseline			
	any	3	4-5	△0	△1	△2	△3
White blood cell decrease	13(65.0%)	2(10.0%)	0	8(40.0%)	9(45.0%)	3(15.0%)	0
Lymphocyte count decrease	18(90.0%)	3(15.0%)	0	4(20.0%)	7(35.0%)	9(45.0%)	0
Neutrophil count decrease	8(40.0%)	3(15.0%)	0	16(80.0%)	2(10.0%)	2(10.0%)	0
Platelet count decrease	14(70.0%)	1(5.0%)	0	10(50.0%)	10(50.0%)	0	0
ALT increase	5(25.0%)	1(5.0%)	0	15(75.0%)	3(15.0%)	1(5.0%)	1(5.0%)
AST increase	9(45.0%)	2(10.0%)	0	11(55.0%)	6(30.0%)	1(5.0%)	2(10.0%)

ALT, alanine aminotransferase; AST, aspartate aminotransferase.

Supplementary table 3. Baseline myelosuppression, ALT and AST grades and the dynamic shifts of deteriorating grades during neoadjuvant therapy(n=20).

			Baseline CTCAE Toxicity Grade				
	Post-baseline Worst Grade		0	1	2	3	4
White blood cell decrease	Max	0	7				
		1	5				
		2	3	2	1		
		3			2		
		4					
						Total	20
	EOT	0	10				
		1	3				
		2	2	2	2		
		3			1		
		4					
						Total	20
Lymphocyte count decrease	Max	0	2				
		1	4	1			
		2	9	1			
		3			2	1	
		4					
						Total	20
	EOT	0	4				
		1	8	1			
		2	3	1			
		3			2	1	
		4					
						Total	20
Neutrophil count decrease	Max	0	12				
		1	2				

		**2**	1		2		
		**3**		1		2	
		**4**					
						**Total**	**20**
	EOT	**0**	13				
		**1**	1		1		
		**2**	1		1		
		**3**		1		2	
		**4**					
						**Total**	**20**
Platelet count decrease	Max	**0**	6				
		**1**	6	3			
		**2**		3	1		
		**3**			1		
		**4**					
						**Total**	**20**
	EOT	**0**	6				
		**1**	6	4			
		**2**		2	1		
		**3**			1		
		**4**					
						**Total**	**20**
ALT increase	Max	**0**	15				
		**1**	3				
		**2**	1				
		**3**	1				
		**4**					
						**Total**	**20**
	EOT	**0**	19				
		**1**	1				
		**2**					
		**3**					

		**4**			
				**Total**	**20**
AST increase	Max	**0**	11		
		**1**	6		
		**2**	1		
		**3**	2		
		**4**			
				**Total**	**20**
	EOT	**0**	15		
		**1**	5		
		**2**			
		**3**			
		**4**			
				**Total**	**20**

ALT, alanine aminotransferase; AST, aspartate aminotransferase; Max, maximal grade during treatment; EOT, grade at the end of treatment.

Supplementary table 4. Characteristics of surgical and postoperative features

	Resection after neoadjuvant therapy (n=19)	Upfront resection (n=203)	t/Z/χ^2	P value
**Demographics**				
Age, years, n (%)			0.574	0.449
＜65	12(63.2%)	145(71.4%)		
≥65	7(36.8%)	58(28.6%)		
Sex, n (%)			0.026	0.873
Male	15(78.9%)	157(77.3%)		
Female	4(21.1%)	46(22.7%)		
BCLC, n (%)			0.334	0.473
0	3(15.8%)	23(11.3%)		
A	16(84.2%)	180(88.7%)		
**Operative variables**				
operation time (min, mean±SD)	135.8±54.2	159.7±51.9	1.907	**0.058**
blood loss (ml, mean±SD)	68.9±47.7	83.3±58.0	1.047	0.296

**Postoperative variables**				
Postoperative hospital stay (days, mean±SD)	16.4±6.7	15.4±5.3	-0.775	0.439
Abdominal drainage time （days, mean±SD）	4.3±2.6	7.1±25.7	0.476	0.635
**Lab tests changes (median, IQR)**				
ΔRBC%	-8.67(-16.325~-4.56)	-6.56(-12.42~0.71)	-1.013	0.311
ΔHb%	-8.28(-14.75~-4.17)	-5.80(-11.46~0.00)	-1.218	0.223
ΔALT%	178.74(106.25~679.19)	502.45(198.83~1030.43)	-2.340	**0.019**
ΔAST%	253.55(135.96~828.43)	519.68(253.69~924.57)	-1.952	0.051
ΔBilirubin%	58.77(32.76~104.33)	49.12(18.32~49.12)	-0.334	0.738
ΔAlbumin%	-9.80(-15.350~-5.51)	-11.33(-17.21~-3.93)	-0.347	0.728
**Postoperative complications, n (%)**	n=11 (57.9%)	n=101 (49.8%)	0.461	0.497
Hydrothorax	8(72.7%)	60(59.4%)		
Hypoproteinemia	7(63.6%)	40(39.6%)		

Massive ascites	4(36.4%)	30(29.7%)
Fever caused by infection	3(27.3%)	25(24.8%)
Pneumonia	1(9.1%)	16(15.8%)
Kaliopenia	1(9.1%)	9(8.9%)
Pelvic effusion	0(0)	8(7.9%)
Spontaneous bacterial peritonitis	0(0)	1(1.0%)

Δ, calculated by subtracting the first corresponding postoperative value from the last pre-operative one. BCLC, Barcelona Clinic Liver Cancer; RBC, red blood cell; Hb, hemoglobin; ALT, alanine aminotransferase; AST, aspartate aminotransferase; DVT, deep vein thrombosis. Operative variables and postoperative variables were indicated as mean plus or minus standard deviation ($\bar{x} \pm SD$) and the Student t-test was used for statistical analysis. Whitney U test was performed to compare lab tests changes because it is a non-normal distribution. Comparative demographics and postoperative complications were performed by Chi-square (χ^2) test. For all tests, P value <0.05 was considered to indicate statistical significance. All analyses were performed using SPSS version 26.0.

Supplementary table 5. Radiographic and pathological responses by investigator assessment

	Number of patients (n=19)	
	RECIST v1.1	mRECIST
**Radiographic response**		
Best overall response		
Complete response	0	3 (15.8%)
Partial response	8 (42.1%)	9 (47.4%)
Objective response	8 (42.1%)	12 (63.2%)
Stable disease	11 (57.9%)	7 (36.8%)
Disease control rate	19 (100%)	19 (100%)
Progressive disease	0	0
**Pathological response**		
Major pathological response	6 (31.6%)	
including: Pathological complete response	2 (10.5 %)	

Data are n (%). RECIST, Response Evaluation Criteria in Solid Tumors; mRECIST, modified RECIST; major pathological response, residual tumor cells <30%; pathological complete response, no residual tumor cell.

Supplementary table 6. Treatment-related adverse events during adjuvant treatment (n=17).

	Any grade	Grade 3
Any TRAE	17 (100.0%)	3 (17.6%)
Lymphocyte count decrease	13 (76.5%)	3 (17.6%)
Platelet count decrease	12 (70.6%)	1 (5.9%)
White blood cell decrease	7 (41.2%)	0
Ascites	7 (41.2%)	0
Blood bilirubin increase	7 (41.2%)	0
Anemia	7 (41.2%)	0
Alkaline phosphatase increase	5 (29.4%)	0
GGT increase	5 (29.4%)	0
Aspartate aminotransferase increase	5 (29.4%)	0
Hypoalbuminemia	4 (23.5%)	0
Neutrophil count decrease	4 (23.5%)	0
Blood lactate dehydrogenase increase	4 (23.5%)	0
Hypothyroidism	3 (17.6%)	0

Electrocardiogram T wave abnormal	2 (11.8%)	0
Alanine aminotransferase increase	2 (11.8%)	0
Fatigue	1 (5.9%)	0
Cardiac troponin T increase	1 (5.9%)	0
Thyroid stimulating hormone increase	1 (5.9%)	0
INR increase	1 (5.9%)	0
Abdominal distension	1 (5.9%)	0
Nausea	1 (5.9%)	0
Hyponatremia	1 5.9%)	0
Activated partial thromboplastin time prolonged	1 (5.9%)	0
Pneumonitis	1 (5.9%)	0

Data are n (%). TRAE, treatment-related adverse event.

Supplementary table 7. Details of adverse events of myelosuppression, ALT and AST during adjuvant treatment(n=17).

CTCAE term	grade			deteriorating grade than baseline			
	any	3	4-5	△0	△1	△2	△3
White blood cell decrease	7(41.2%)	0	0	12(70.6%)	4(23.5%)	1(5.9%)	0
Lymphocyte count decrease	13(76.5%)	3(17.6%)	0	6(35.3%)	5(29.4%)	3(17.6%)	2(11.8%)
Neutrophil count decrease	4(23.5%)	0	0	15(88.2%)	1(5.9%)	0	0
Platelet count decrease	12(70.6%)	1(5.9%)	0	10(58.8%)	6(35.3%)	1(5.9%)	0
ALT increase	2(11.8%)	0	0	15(88.2%)	2(11.8%)	0	0
AST increase	5(29.4%)	0	0	12(70.6%)	5(29.4%)	0	0

ALT, alanine aminotransferase; AST, aspartate aminotransferase.

Supplementary table 8. Baseline myelosuppression, ALT and AST grades and the dynamic shifts of deteriorating grades during adjuvant therapy (n=17).

			**Baseline CTCAE Toxicity Grade**				
	**Post-baseline Worst Grade**		**0**	**1**	**2**	**3**	**4**
White blood cell decrease	Max	**0**	10				
		**1**	3				
		**2**	1	1	2		
		**3**					
		**4**					
						**Total**	**17**
	EOT	**0**	13				
		**1**	1		1		
		**2**		1	1		
		**3**					
		**4**					
						**Total**	**17**
Lymphocyte count decrease	Max	**0**	3	1			
		**1**	5	1			
		**2**	3		1		
		**3**	2			1	
		**4**					
						**Total**	**17**

	EOT	**0**	7	1			
		**1**	4	1			
		**2**	1		1		
		**3**	1			1	
		**4**					
						**Total**	**17**
Neutrophil count decrease	Max	**0**	13				
		**1**					
		**2**		1	2	1	
		**3**					
		**4**					
						**Total**	**17**
	EOT	**0**	13	1		1	
		**1**			2		
		**2**					
		**3**					
		**4**					
						**Total**	**17**
Platelet count decrease	Max	**0**	5				
		**1**	4	4			
		**2**	1	1	1		
		**3**			1		
		**4**					
						**Total**	**17**

	EOT	**0**	6	1			
		**1**	3	4			
		**2**	1		1		
		**3**			1		
		**4**					
						**Total**	**17**
ALT increase	Max	**0**	15				
		**1**	2				
		**2**					
		**3**					
		**4**					
						**Total**	**17**
	EOT	**0**	16				
		**1**	1				
		**2**					
		**3**					
		**4**					
						**Total**	**17**
AST increase	Max	**0**	12				
		**1**	5				
		**2**					
		**3**					
		**4**					
						**Total**	**17**

EOT	**0**	13		
	**1**	4		
	**2**			
	**3**			
	**4**			
			**Total**	**17**

ALT, alanine aminotransferase; AST, aspartate aminotransferase; Max, maximal grade during treatment; EOT, grade at the end of treatment.

A phase Ib study of neoadjuvant tislelizumab with stereotactic body radiotherapy in patients with resectable hepatocellular carcinoma

Protocol

version number： v2.0

date: 22th Jan 2022

Sponsor：Shandong Cancer Hopsital Affiliated to Shandong First Medical University

Signing page of PI

(Be omitted)

A phase Ib study of neoadjuvant tislelizumab with stereotactic body radiotherapy in patients with resectable hepatocellular carcinoma

Background:

(Be omitted)

Study design:

Notable-HCC is a prospective, single-centered, phase Ib explotary study, aiming to evaluate the safety and effectiveness of neoadjuvant stereotactic body radiotherapy (SBRT) plus an an immune checkpoint inhibitor (ICI) prior to hepatic resection in patients with resectable HCC. This study will not compare or evaluate the outcome of different treatments. If the results of this trial are satisfied and encouraging, we will discuss the possibility of further phase II to III trials. Twenty participants are planned to be enrolled in this trial. The study will start on March 1, 2022, and is anticipated to be completed on December 31, 2024.

Tislelizumab (BGB-A317) is a human monoclonal antibody (HuMAb; immunoglobulin G4 [IgG4]) manufactured by BeiGene that inhibits the PD-1 receptor. It is engineered with a nullified Fc portion of the antibody to minimise binding to FcγR on macrophages in order to abrogate antibody-dependent phagocytosis.

Eligible patients will receive SBRT (8 Gy × 3 fractions, every other day) on day 1, day 3 and day 5; the first dose (200 mg) of tislelizumab will be administered concurrently on day 1, then the second dose will be administered on day 22 (the first day of week 4, ± 3 days). Then on day 50 (the first day of week 8, ± 7 days), curative liver resection of HCC will be scheduled. In the adjuvant setting, starting four weeks after the curative resection (day 78, the first day of week 12, ± 7 days), patients received 200 mg of tislelizumab intravenously every 3 weeks for up to 1 year or until disease progression or intolerable toxicity

Eligibility criteria

Notable-HCC will recruit HCC patients who are candidates for hepatic resection, with a confirmed diagnosis of HCC by biopsy or by the noninvasive diagnostic criteria of the American Association for the Study of Liver Diseases(AASLD).

Inclusion criteria:

1. Written informed consent for the trial.
2. Aged ≥18 years, ≤80 years.
3. Willing to provide tissue from an excisional biopsy of a tumor lesion.
4. Confirmed diagnosis of HCC. The diagnosis can be established radiographically by the criteria of the American Association for the Study of Liver Disease (AASLD), or by histologic diagnosis from a core biopsy.
5. Measurable disease by computed tomography (CT)-scan or magnetic resonance imaging (MRI) defined by the Response Evaluation Criteria In Solid Tumors (RECIST) 1.1 criteria and HCC-specific modified RECIST (mRECIST).
6. Medically fit to undergo surgery as determined by the treating medical and surgical oncology team
7. Eastern Cooperative Oncology Group (ECOG) performance status 0 or 1
8. Adequate organ and marrow function as defined below:

1) leukocytes ≥3,000/mcL
2) absolute neutrophil count ≥1,500/mcL
3) platelets ≥100,000/mcL
4) total bilirubin ≤ 2 × institutional upper limit of normal (ULN)
5) aspartate aminotransferase (AST)/ alanine aminotransferase (ALT) ≤ 3 × institutional ULN

HCC patients who presented with chronic viral hepatitis, baseline bone marrow suppression or liver dysfunction were eligible for enrollment if they demonstrated positive response to

symptomatic treatment and were assessed by the investigators as being able to tolerate the neoadjuvant treatment and subsequent hepatic resection. The corresponding indicators needed to be dynamically monitored throughout the course of treatment.

6) creatinine ≤ 1.5 × institutional ULN

7) estimated glomerular filtration rate (GFR) ≥50 mL/min/1.73 m^2 (according to the Cockcroft-Gault formula)

9. Overall Child-Pugh class A

10. Documented virology status of hepatitis, as confirmed by screening tests for hepatitis B virus (HBV) and hepatitis C virus (HCV)

1) For patients with active HBV: HBV DNA <2000 IU/mL during screening, and have initiated anti-HBV treatment at least 14 days prior to SBRT and willingness to continue anti-HBV treatment during the study (per local standard of care; e.g., entecavir).

2) Patients with HCV, either with resolved infection (as evidenced by detectable antibody and negative viral load) or chronic infection (as evidenced by detectable HCV RNA), are eligible.

11. Participants with a prior or concurrent malignancy whose natural history or treatment does not have the potential to interfere with the safety or efficacy assessment of the investigational regimen are eligible for this trial.

12. Female patients of childbearing potential should have a negative serum pregnancy test within 24 hours of their first dose of Investigational Medicinal Product (IMP)

13. Women of childbearing potential must be willing to use a highly effective method of contraception for the course of the study through 6 months after the last dose of IMP. Note: Abstinence is acceptable if this is the usual lifestyle and preferred contraception for the patient.

14. Sexually active males must agree to use an adequate method of contraception starting with the first dose of IMP through 7 months after the last dose of study therapy. Note:

Abstinence is acceptable if this is the usual lifestyle and preferred contraception for the patient.

Exclusion criteria:

1. Extrahepatic metastasis.
2. Prior systemic anticancer treatment for HCC, including an anti-PD-1, anti-PD-L1 or anti-CTLA-4 antibody.
3. Prior orthotopic liver transplantation.
4. Prior abdominal irradiation.
5. Any major surgery within the 3 weeks prior to enrolment.
6. Hepatic encephalopathy.
7. Ascites that is refractory to diuretic therapy.
8. Currently receiving anticancer therapy (chemotherapy, radiation therapy, immunotherapy or biologic therapy) or has participated or is participating in a study of an IMP or used an investigational device within 4 weeks of the first dose of IMP.
9. Diagnosis of immunodeficiency or is receiving systemic steroid therapy or any other form of immunosuppressive therapy.
10. Known history of active Bacillus tuberculosis (TB) infection.
11. History of known hypersensitivity to any monoclonal antibody or any of their excipients.
12. Known additional malignancy that is progressing or requires active treatment. Exceptions include basal cell carcinoma of the skin or squamous cell carcinoma of the skin that has undergone potentially curative therapy or in situ cervical cancer.
13. Active autoimmune disease that has required systemic treatment in the past 2 years (i.e. use of disease-modifying agents, corticosteroids or immunosuppressive drugs). Replacement therapy (e.g., thyroxine, insulin, or physiologic corticosteroid replacement

therapy for adrenal or pituitary insufficiency) is not considered a form of systemic treatment.

14. Active infection requiring systemic therapy, with exceptions relating to Hepatitis B and C virus infection.
15. History or current evidence of any condition, therapy, or laboratory abnormality that might confound the results of the trial, interfere with the patient's participation for the full duration of the trial, or is not in the best interest of the patient to participate, in the opinion of the treating Principal Investigator (PI).
16. Known psychiatric or substance abuse disorders that would interfere with cooperation with the requirements of the trial
17. Pregnant or breastfeeding
18. Known history of human immunodeficiency virus (HIV; HIV 1/2 antibodies)
19. Received a live vaccine within 30 days of the first dose of IMP administration.

Study procedures

The consent form will be signed by a liver surgeon with the participant or his delegate. Patients will undergo baseline tumor imaging including CT scans of the chest, abdomen and pelvis, and by contrast-enhanced MRI scans of the liver at screening. At posttreatment time points prior to surgery (on Day 50), 4 weeks after surgery and then every 3 months after surgery, tumor imaging will be repeated using contrast-enhanced MRI. Triple-phase CT of the liver is an acceptable alternative for intrahepatic staging in patients with contraindications to MRI. Baseline CT/MRI scans do not need to be repeated if obtained within 35 days of the first SBRT. The same method used for assessment at baseline must then be used at all subsequent time points.

Participants will require a full hepatitis serology screen prior to enrolment in the study, which includes HBV and HCV serology. In patients with positive serology for either virus, baseline HBV DNA and HCV quantitative RNA levels will be requested. Participants who are confirmed to have chronic and active hepatitis B and/or C (i.e., with detectable HBV DNA or HCV RNA at baseline)

will have their viral load (HCV RNA and/or HBV DNA as appropriate) monitored at each cycle and at the end of the treatment follow-up visit.

A baseline core tumor biopsy and Peripheral Blood Mononuclear Cell (PBMC) will be collected from participants at screening, and sample tumor tissue from the surgical specimen will be snap-frozen and stored for the future relevant studies.

Treatment will consist of 8 Gy × 3 fractions SBRT together with 2 cycles of tislelizumab 200mg administered intravenously with an interval of 3 weeks.

Patients will be reviewed following the completion of SBRT and tislelizumab treatment (Follow-up visit 1; FU1) prior to surgery. Before surgery, RECIST v1.1 and HCC-specific mRECIST criteria will be used to determine patient response to treatment, including complete response (CR), partial response (PR) and objective response rate (ORR). PBMCs will be collected again.

Hepatic resection will be performed as per the standard of care. Safety FU2 will be conducted after the first dose of the post-resection tislelizumab. All adverse events (AEs) that occur prior to the visit will be recorded. Participants with ongoing AEs at the visit will be followed up by the PI or delegated until resolution or stabilisation of the event. Following resection, participants will be assessed every 3 months (±7 days) thereafter to collect information regarding disease status and survival. Long-term follow-up will continue for a total of 2 years for each patient.

All personal information of the enrolled participants will be maintained and protected in the hospital information system, and be accessible only to the authorized medical staffs to protect the confidentiality. PIs of the trial have access to the final entire trial dataset.

Endpoints

Primary endpoints were the number of patients experiencing a surgery delay over 6 weeks (calculated from the planned date of surgery on day 50), ORR after the neoadjuvant therapy according to the RECIST v1.1 and mRECIST criteria, pathological response rates, and the safety and tolerability of the combination neoadjuvant therapy with SBRT+ tislelizumab, as well as the adjuvant therapy with tislelizumab assessed with the Common Terminology Criteria for Adverse Events (CTCAE) version 5.0. Secondary endpoints were disease-free survival (DFS) and over

survival (OS) rates assessed every 3 or 6 months after hepatic resection, until 2 years after the resection. Exploratory endpoints included patients' immune response, incidence of surgical complications, and mortality rate.

Statistical analysis

A total of 20 patients were planned to be enrolled in this trail. All participants who complete at least one dose of tislelizumab and one fractions of SBRT will be included in the safety analysis (SAS). All participants in SAS who complete curative HCC resection will be included in the efficacy analysis (EAS). The baseline demographic and clinicopathological variables will be presented by descriptive analyses. RECIST 1.1/mRECIST response rates (CR, PR and ORR) and pathological response rates (MPR [defined as residual tumor cells of 30% or fewer in the resected specimen], pCR [complete pathological response], etc) will be presented descriptively. Statistical analyses of clinical parameters were done with SPSS, and the biomarker analyses were done in R (version 4.1.2) and SAS (version 9.4).

Discussion

(Be omitted)
