# Supplementary material for: Neoadjuvant tislelizumab plus stereotactic body radiotherapy and adjuvant tislelizumab in early-stage resectable hepatocellular carcinoma: the Notable-HCC phase 1b trial
Source: Nat Commun. 2024 Apr 16;15:3260. doi: 10.1038/s41467-024-47420-3 (PMC11021407; doi:10.1038/s41467-024-47420-3)
Supplement: Supplementary file 3 — Description of Additional Supplementary Files [file 41467_2024_47420_MOESM3_ESM.pdf]

Description of Additional Supplementary Files

Supplementary Movie 1: The Elekta Unity MRI-linac system enables visualization of all anatomical changes during the course of radiotherapy.
