# Supplementary material for: Neoadjuvant tislelizumab plus stereotactic body radiotherapy and adjuvant tislelizumab in early-stage resectable hepatocellular carcinoma: the Notable-HCC phase 1b trial
Source: Nat Commun. 2024 Apr 16;15:3260. doi: 10.1038/s41467-024-47420-3 (PMC11021407; doi:10.1038/s41467-024-47420-3)

Patient 01

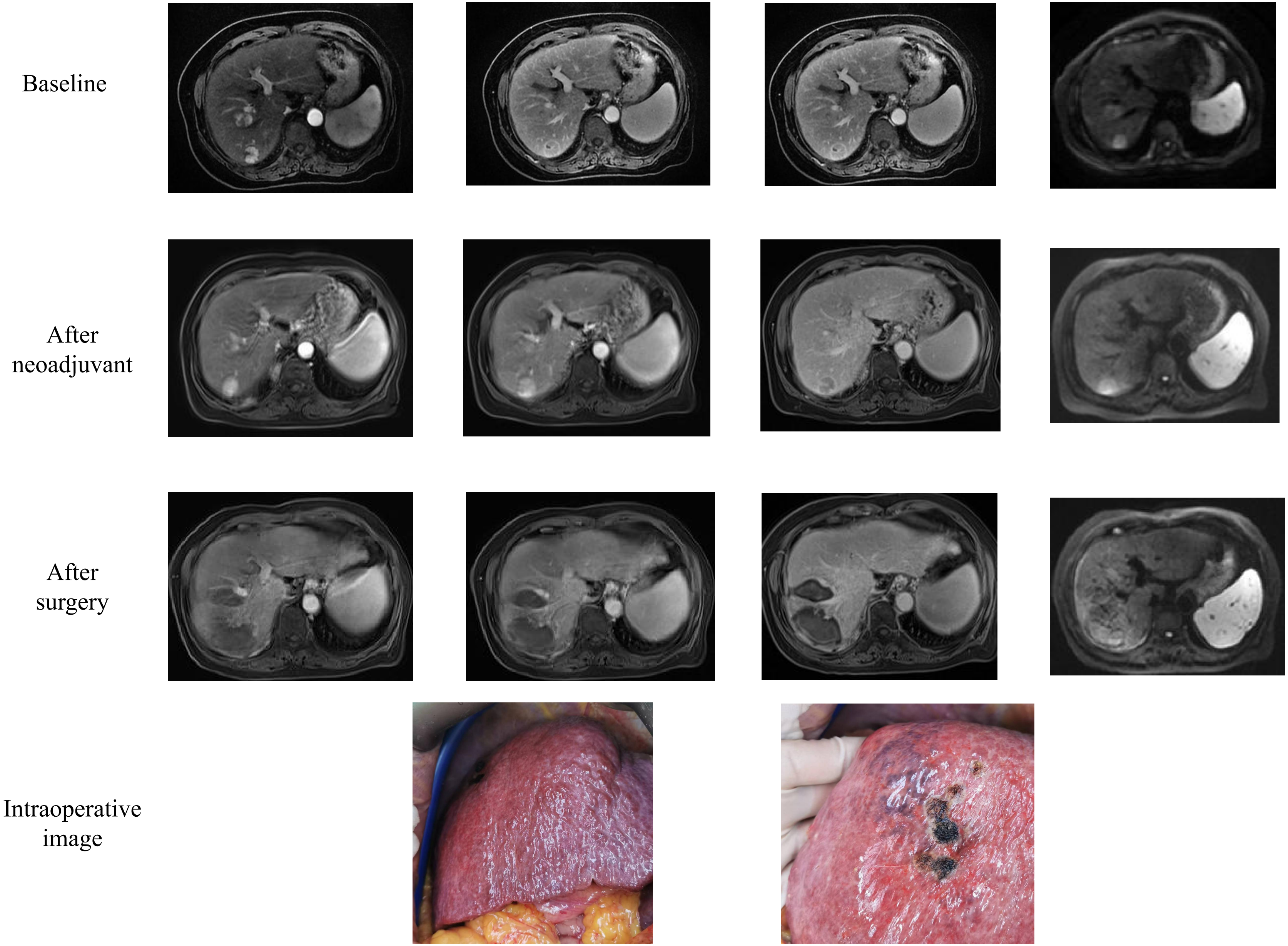
Baseline
After neoadjuvant
After surgery
Intraoperative image

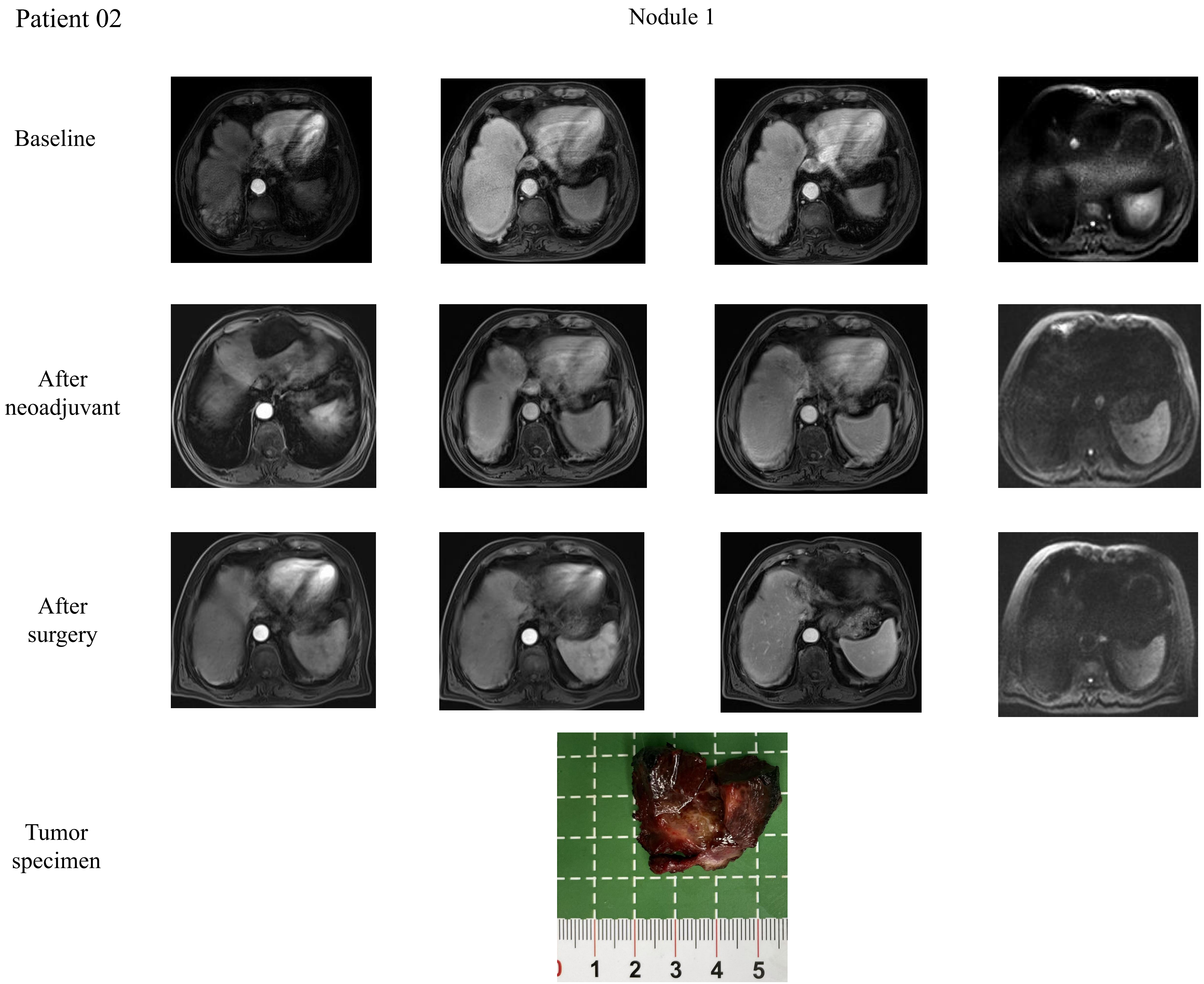
Patient 02
Nodule 1
Baseline
After neoadjuvant
After surgery
Tumor specimen
1 2 3 4 5

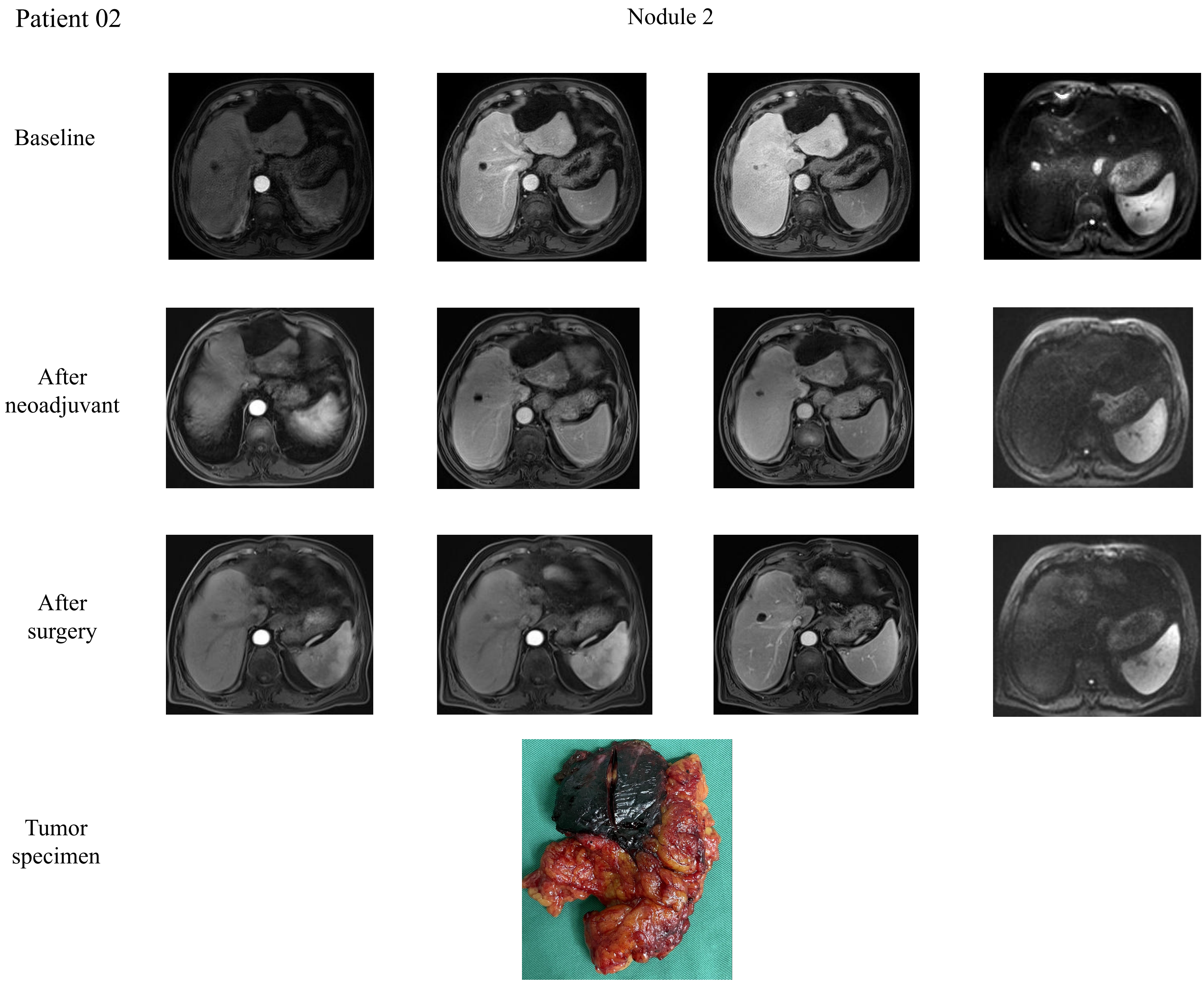
Patient 02
Nodule 2
Baseline
After neoadjuvant
After surgery
Tumor specimen

Patient 03

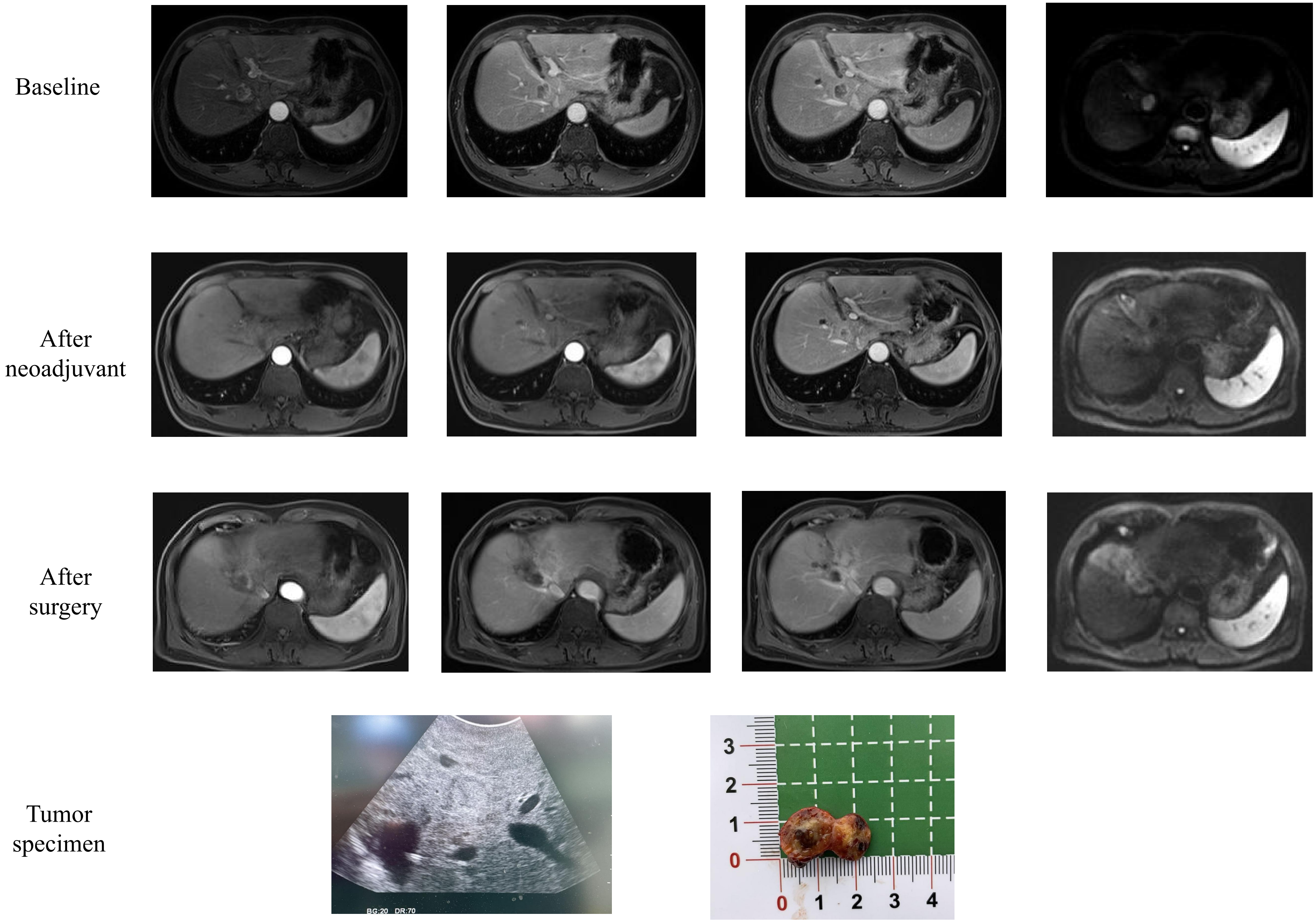

Patient 04

Baseline

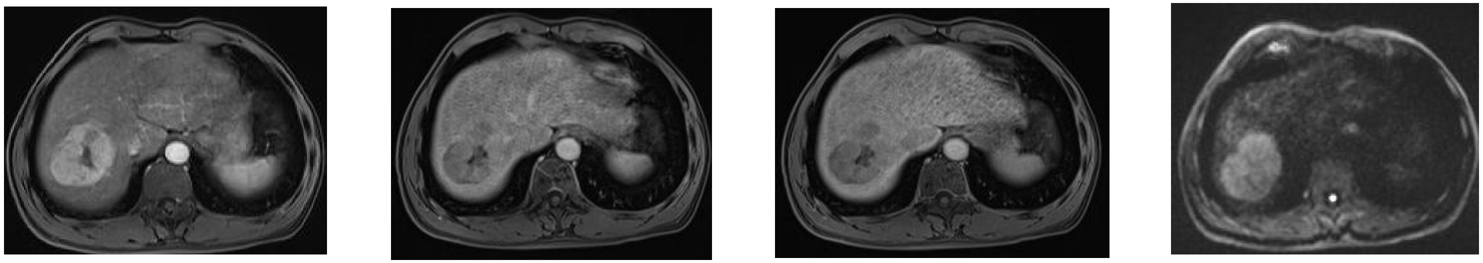

After neoadjuvant

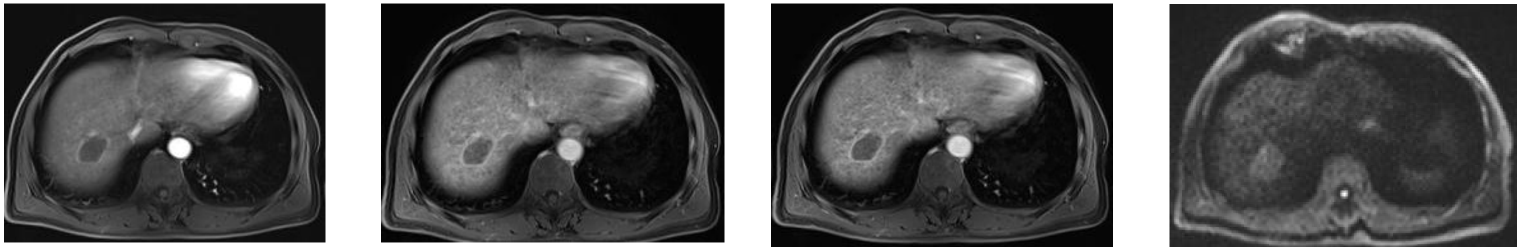

After surgery

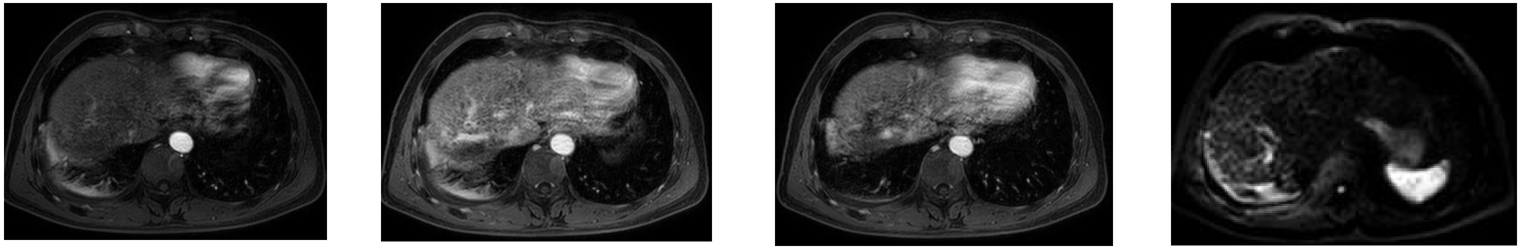

Tumor specimen

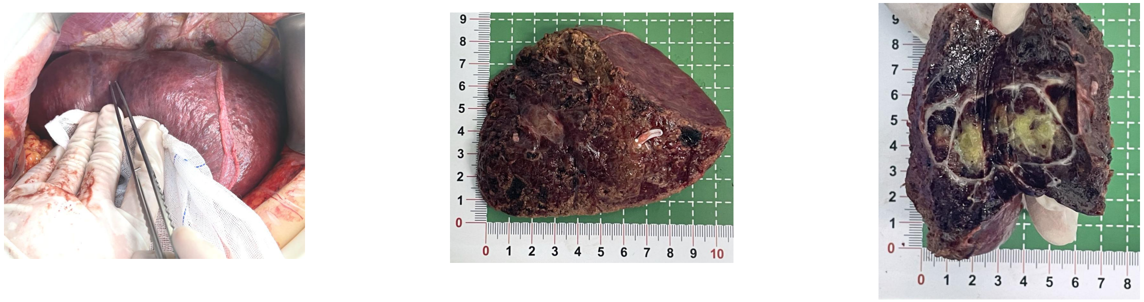

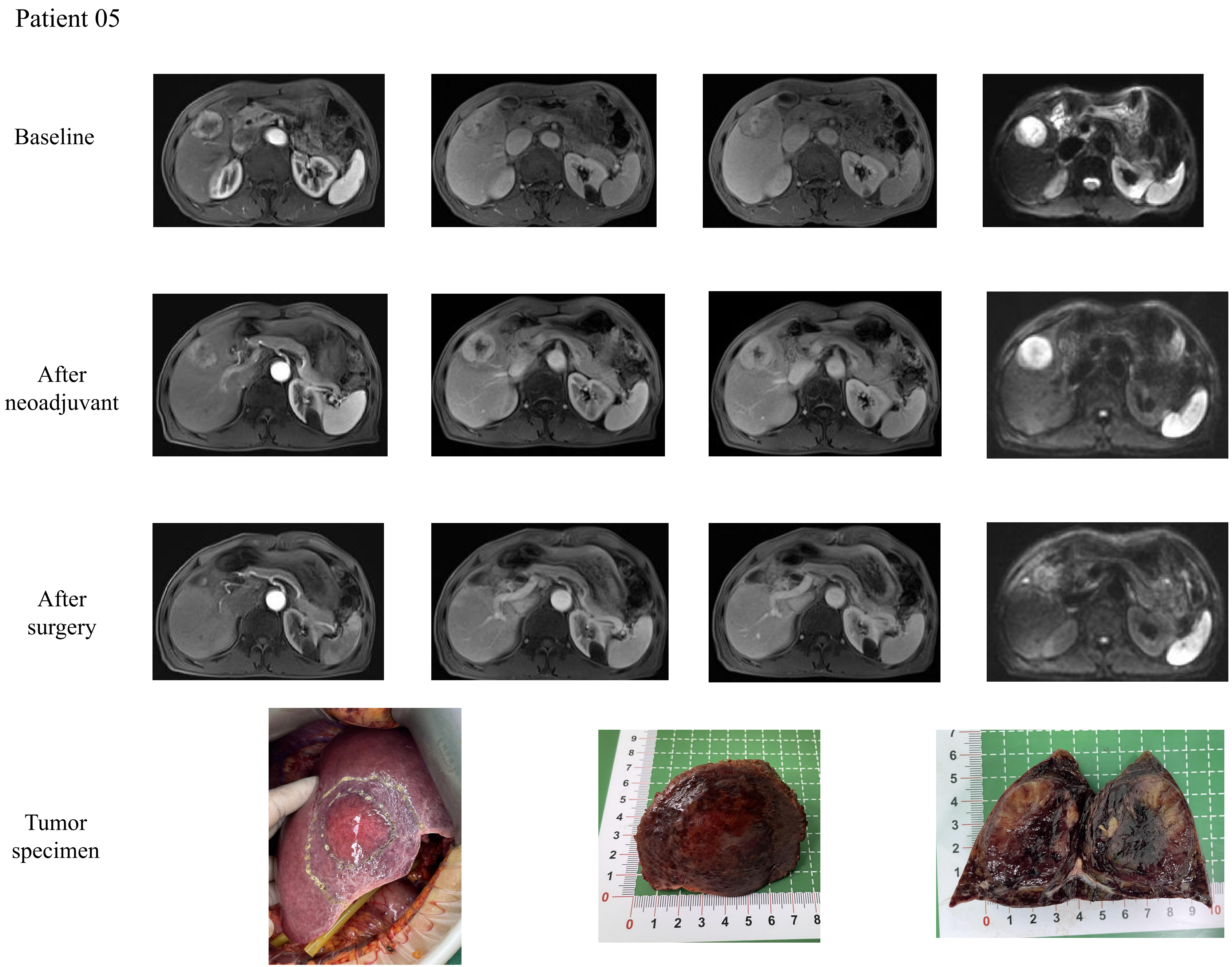
Patient 05
Baseline
After neoadjuvant
After surgery
Tumor specimen

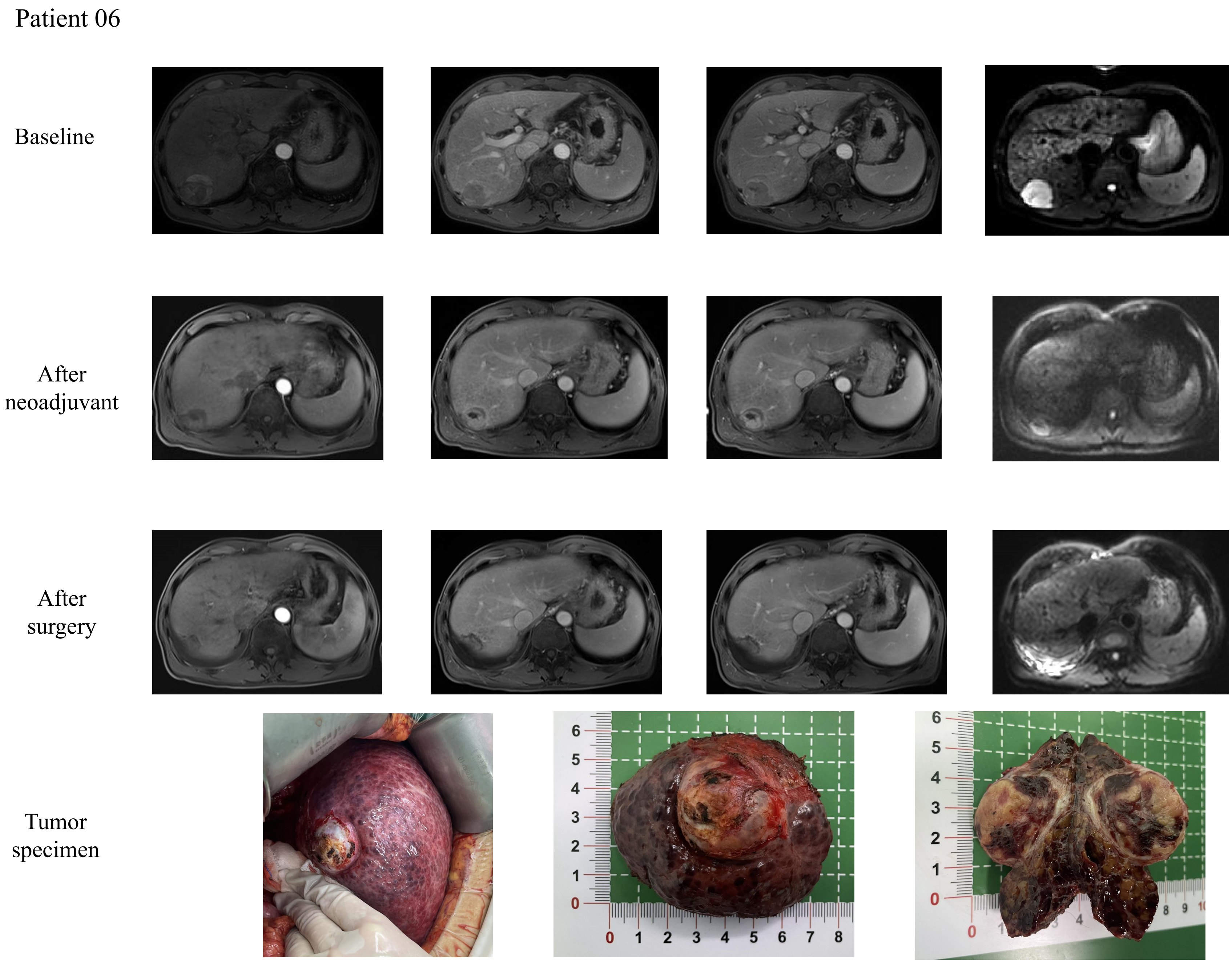
Patient 06
Baseline
After neoadjuvant
After surgery
Tumor specimen

Patient 07

Baseline

After neoadjuvant

After surgery

Tumor specimen

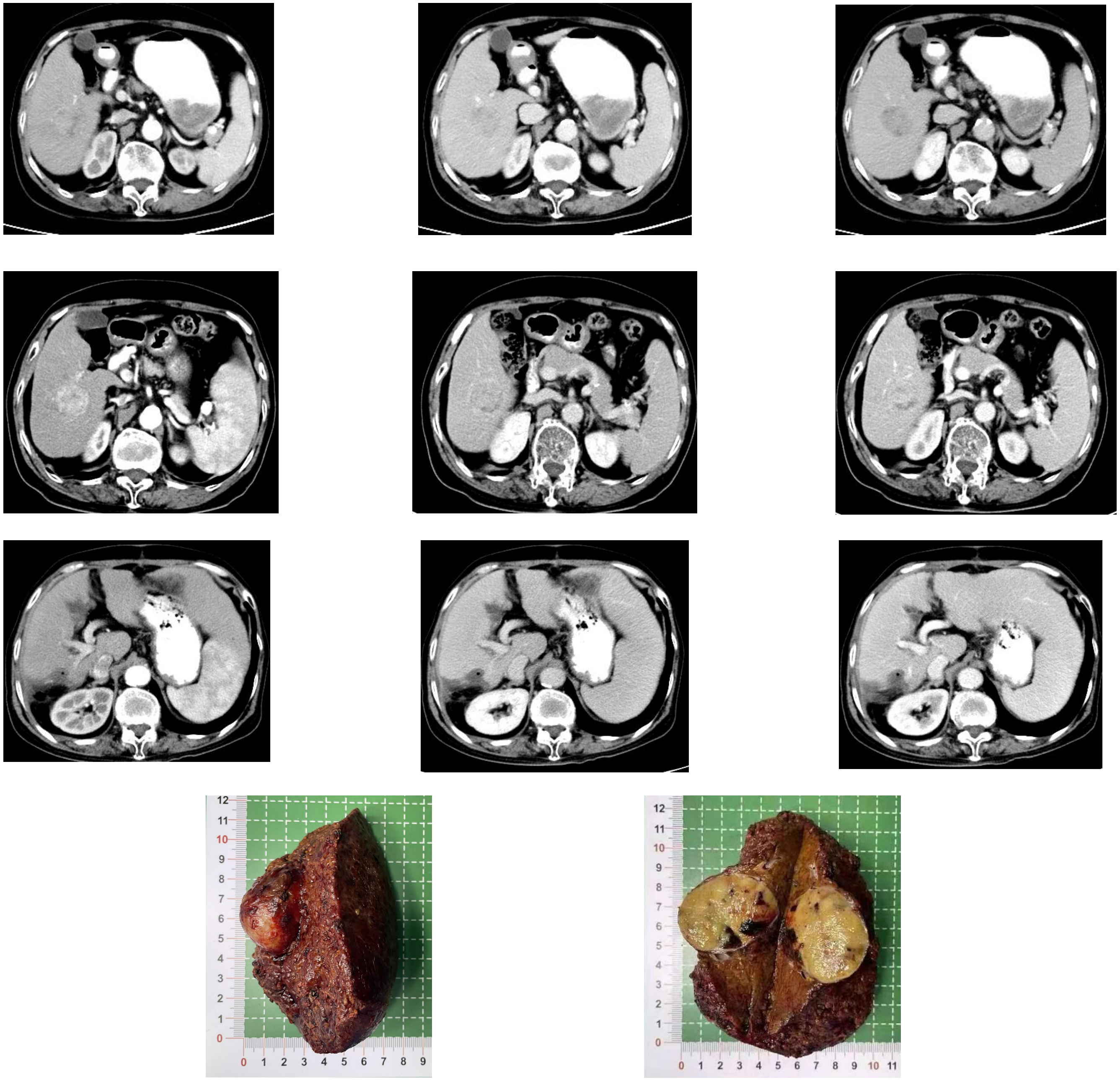

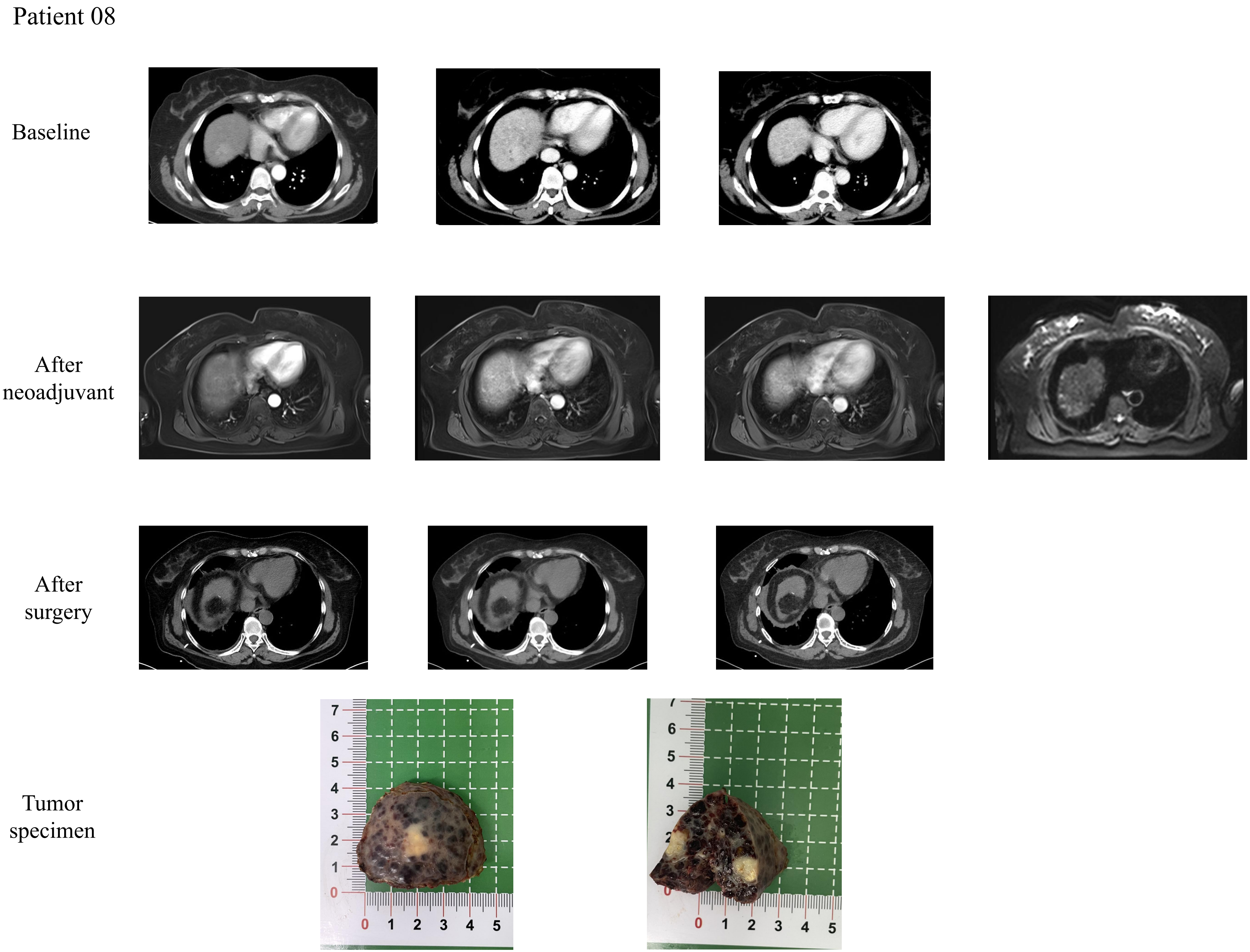
Patient 08
Baseline
After neoadjuvant
After surgery
Tumor specimen

Patient 09

Baseline

After neoadjuvant

After surgery

Tumor specimen

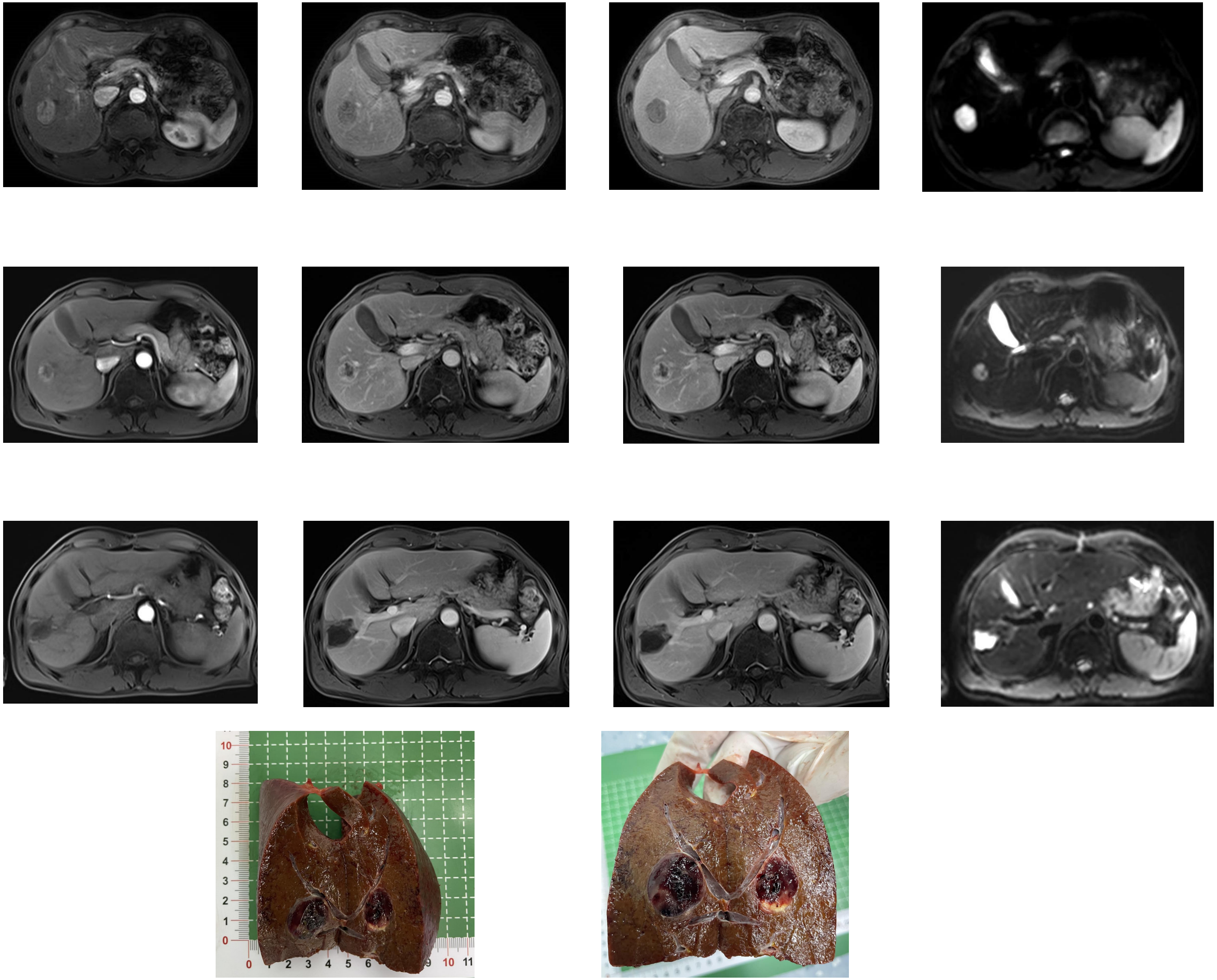

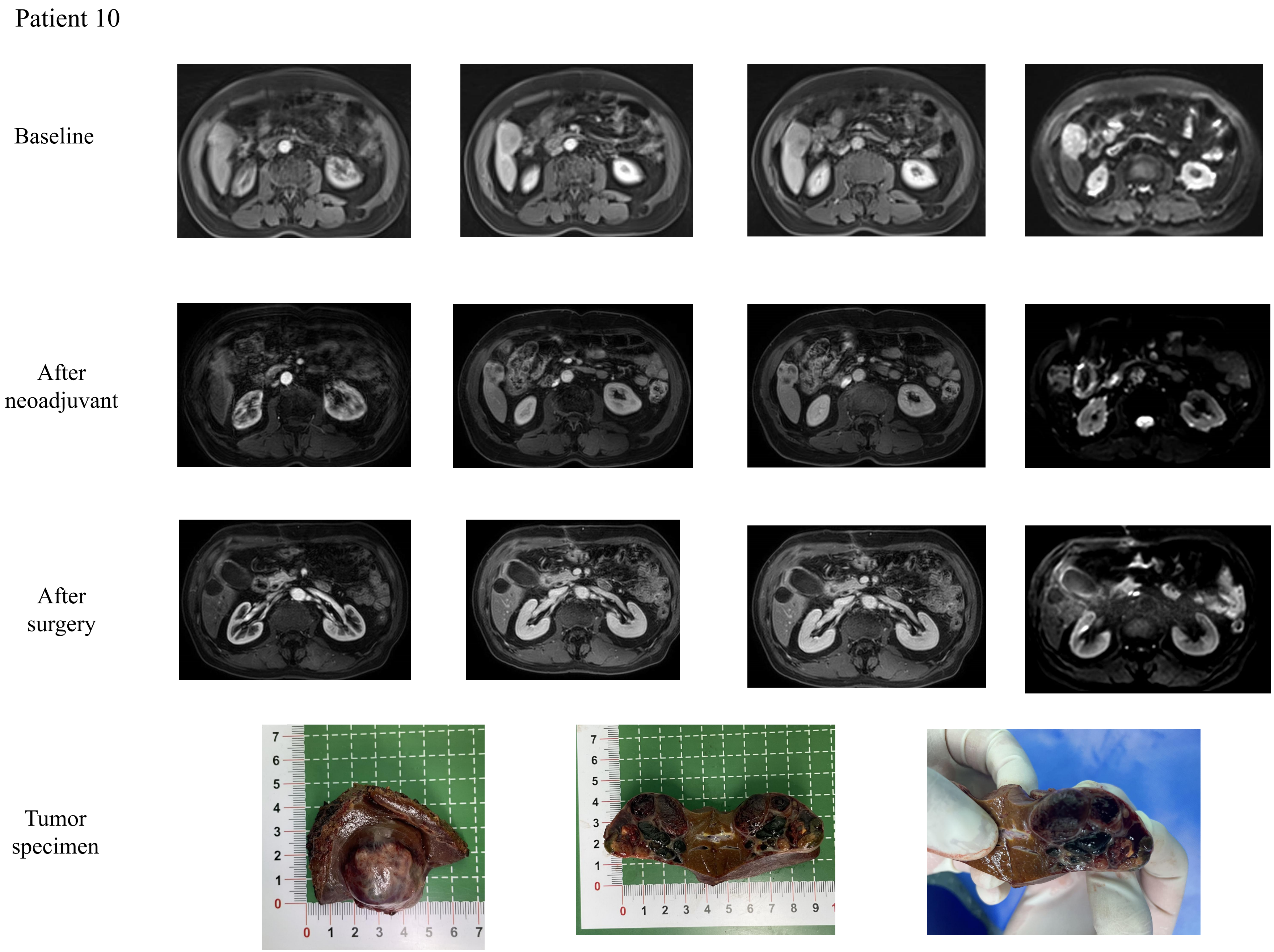
Patient 10
Baseline
After
neoadjuvant
After
surgery
Tumor
specimen

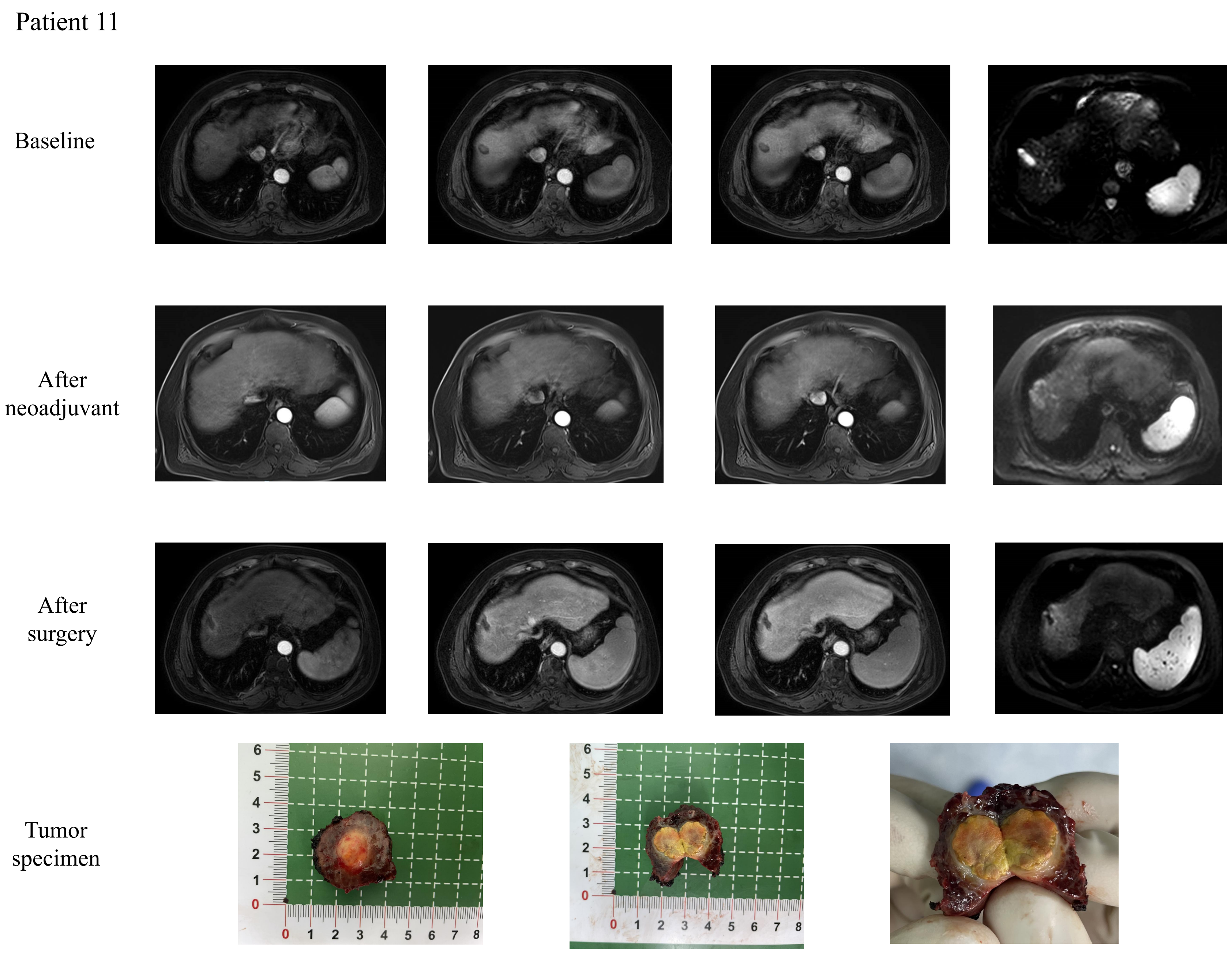
Patient 11
Baseline
After neoadjuvant
After surgery
Tumor specimen

Patient 12

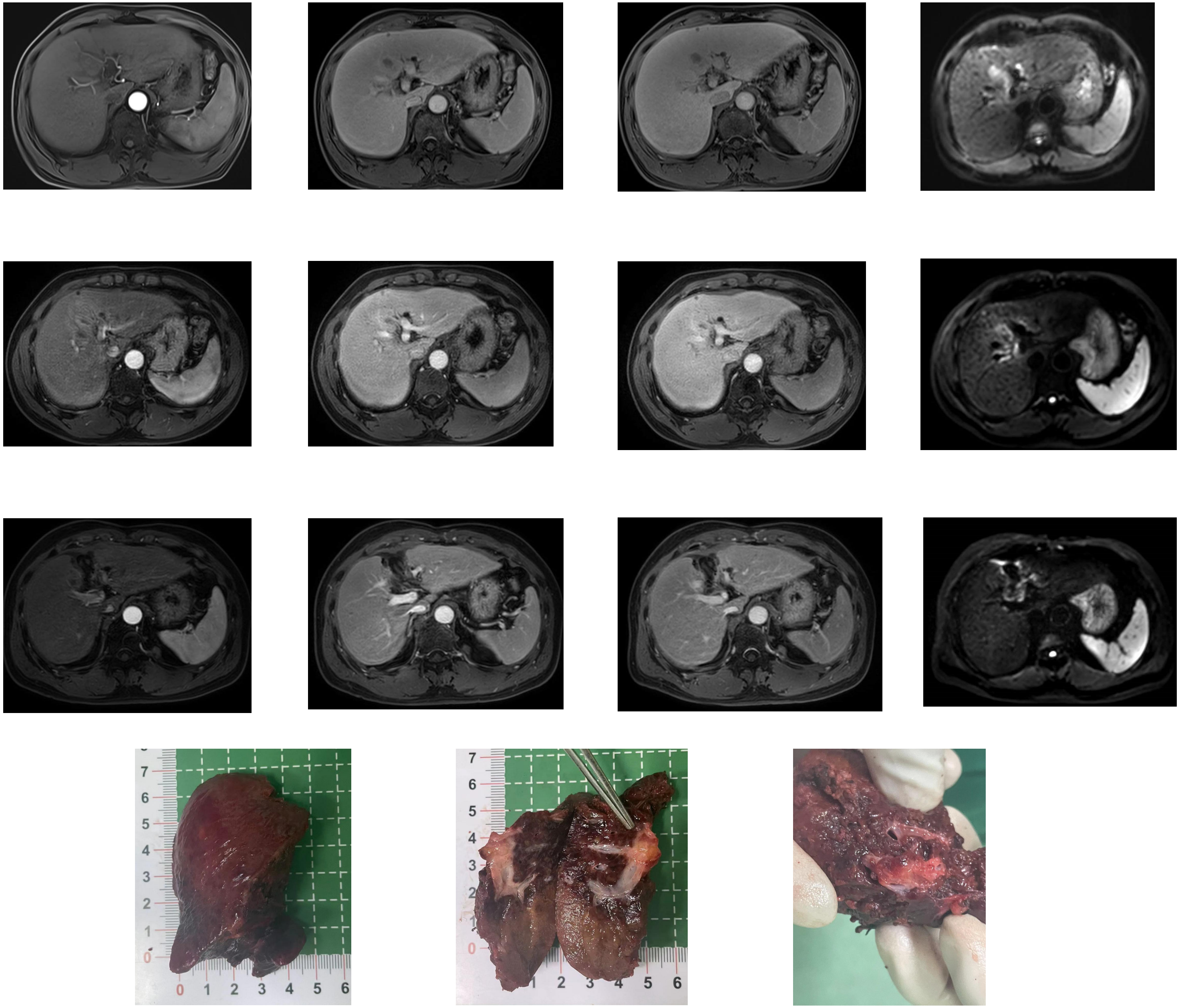

Baseline

After neoadjuvant

After surgery

Tumor specimen

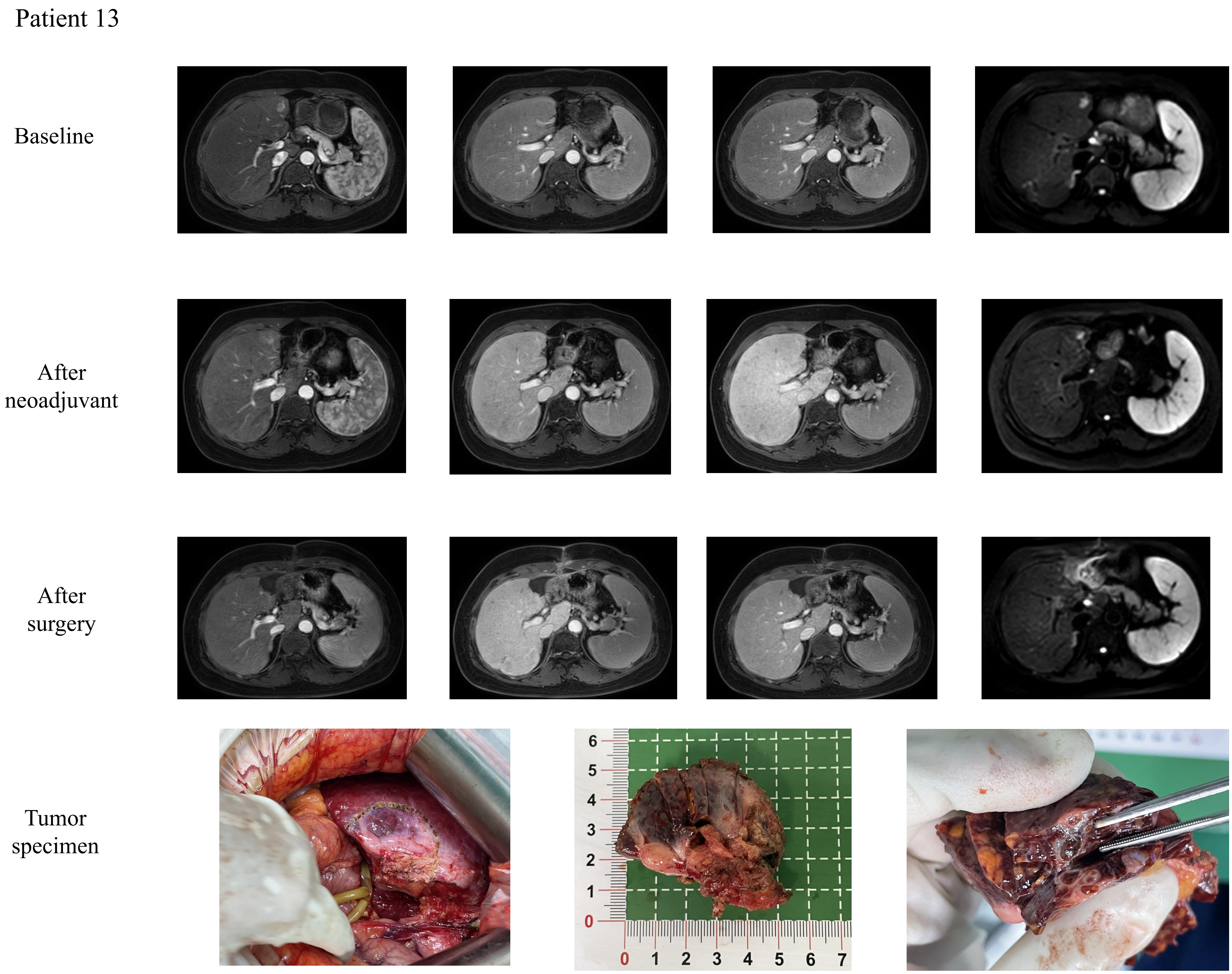
Patient 13
Baseline
After neoadjuvant
After surgery
Tumor specimen
6 5 4 3 2 1 0
0 1 2 3 4 5 6 7

Patient 14

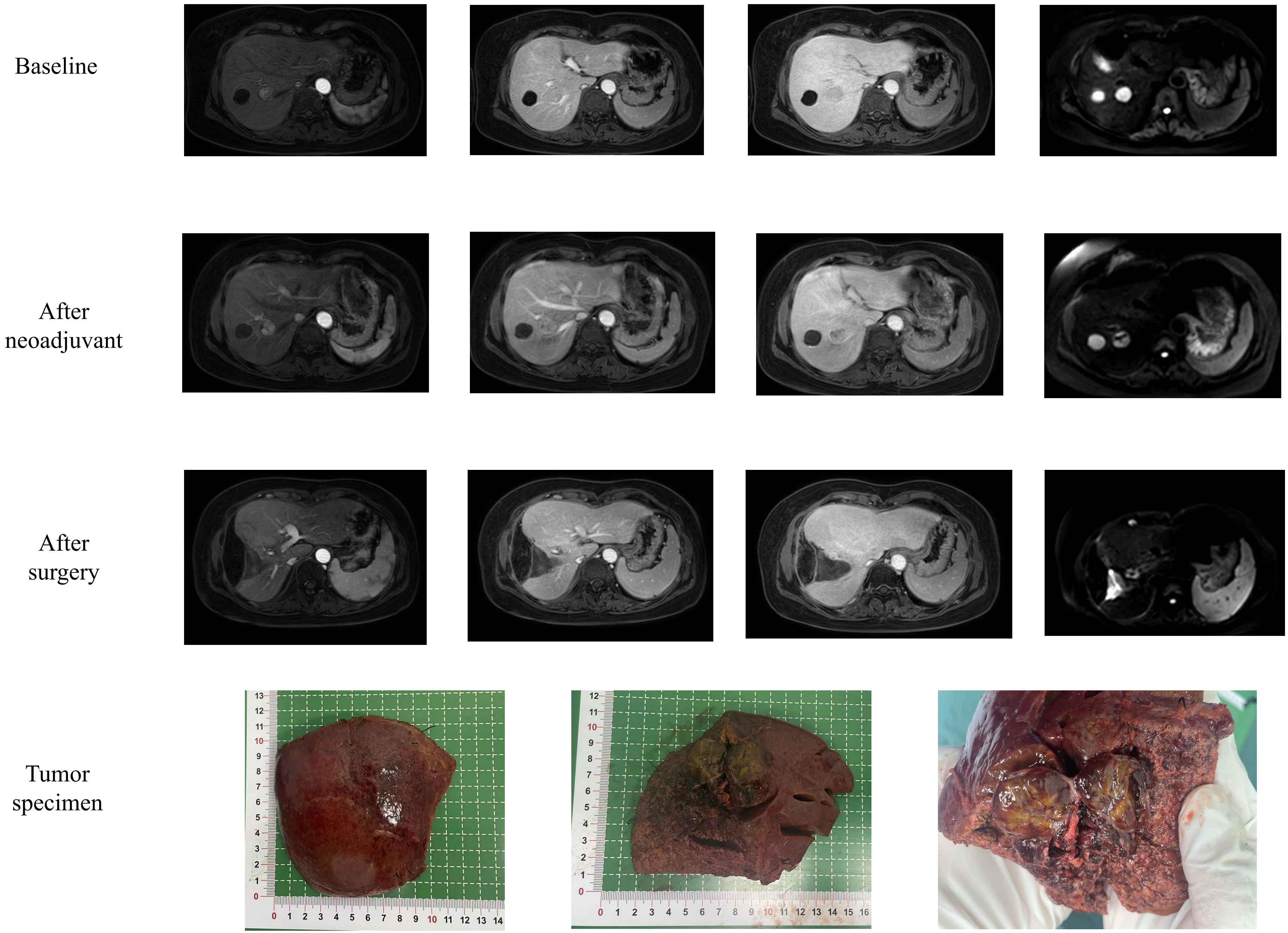

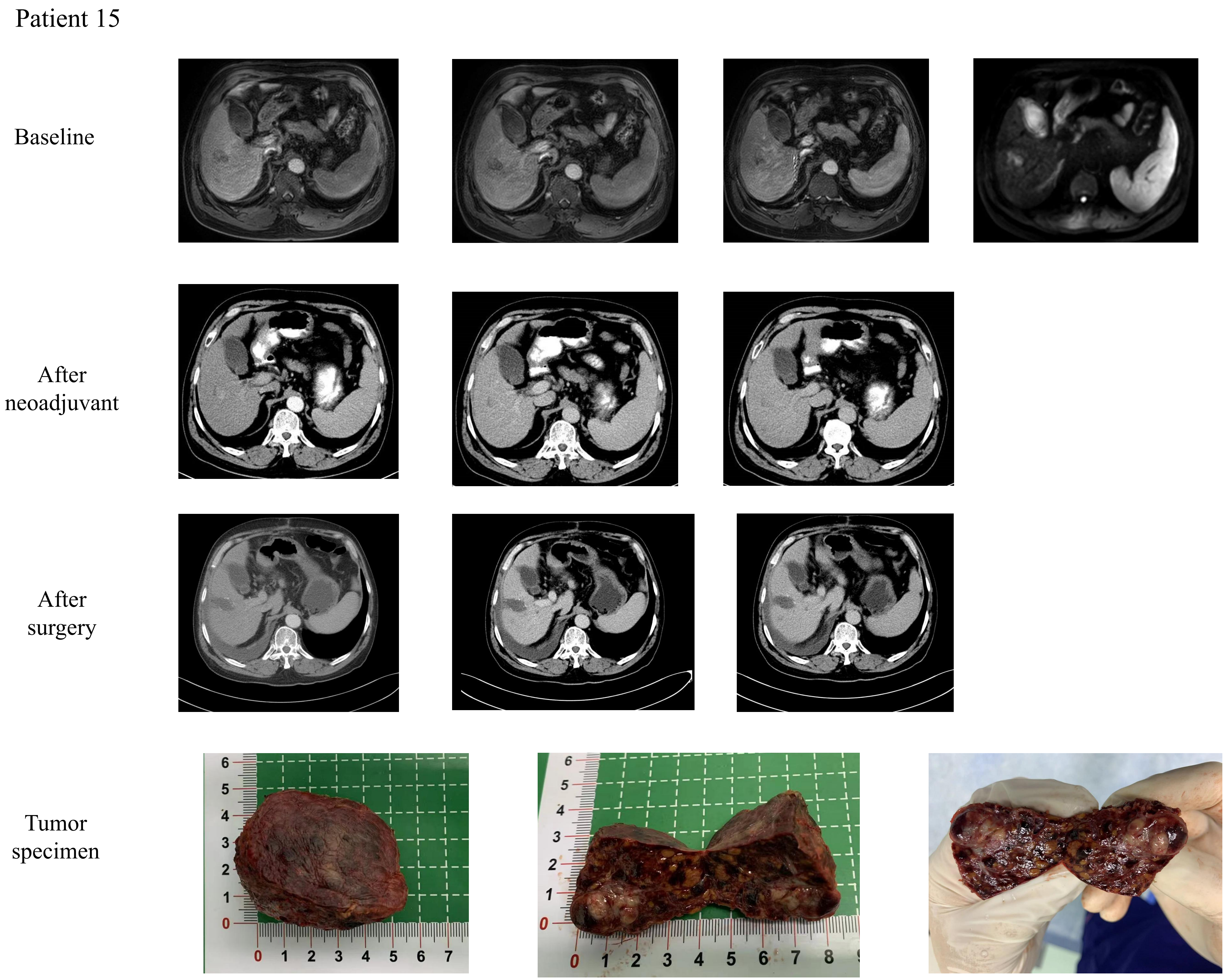
Patient 15
Baseline
After neoadjuvant
After surgery
Tumor specimen

Patient 16

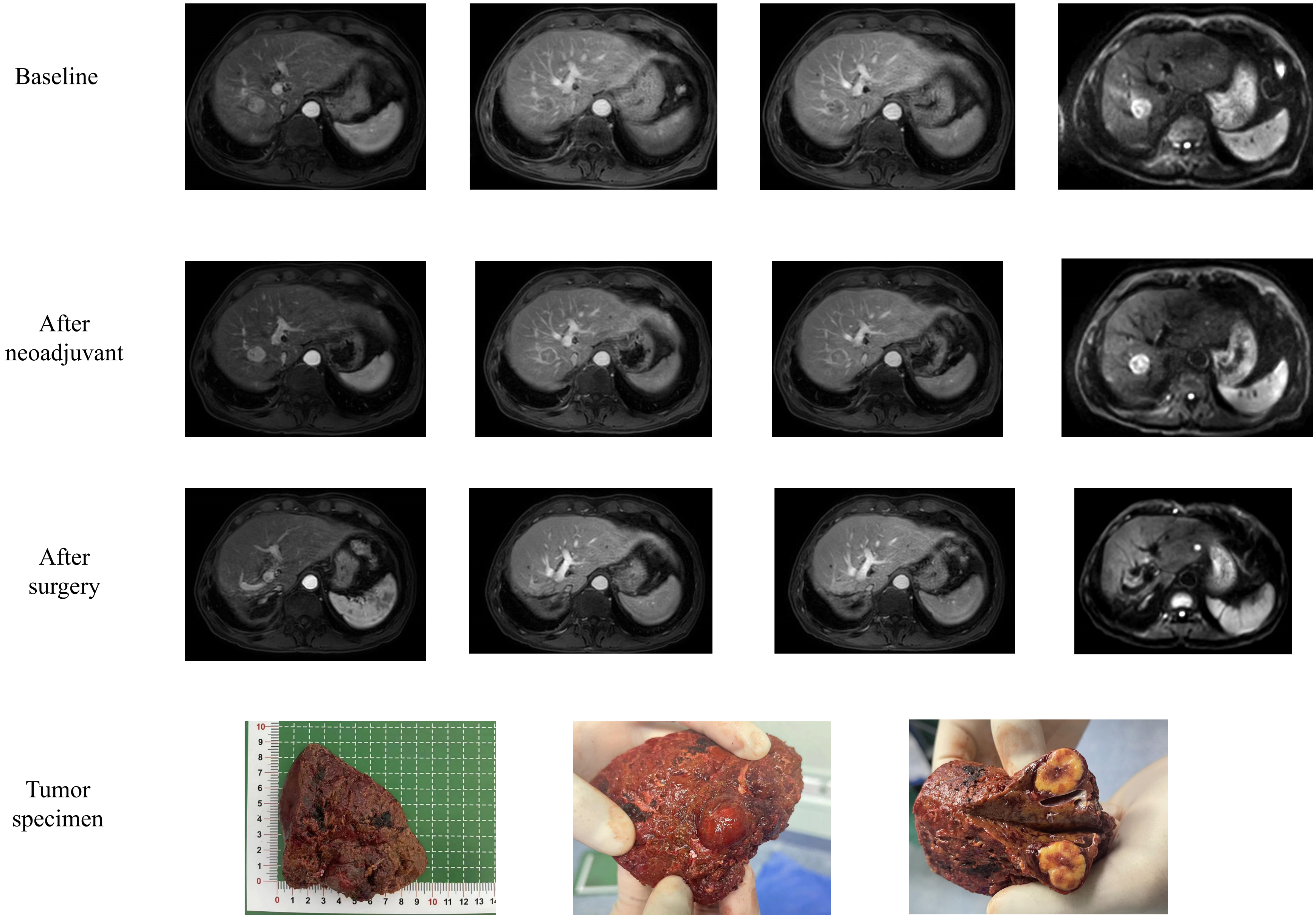

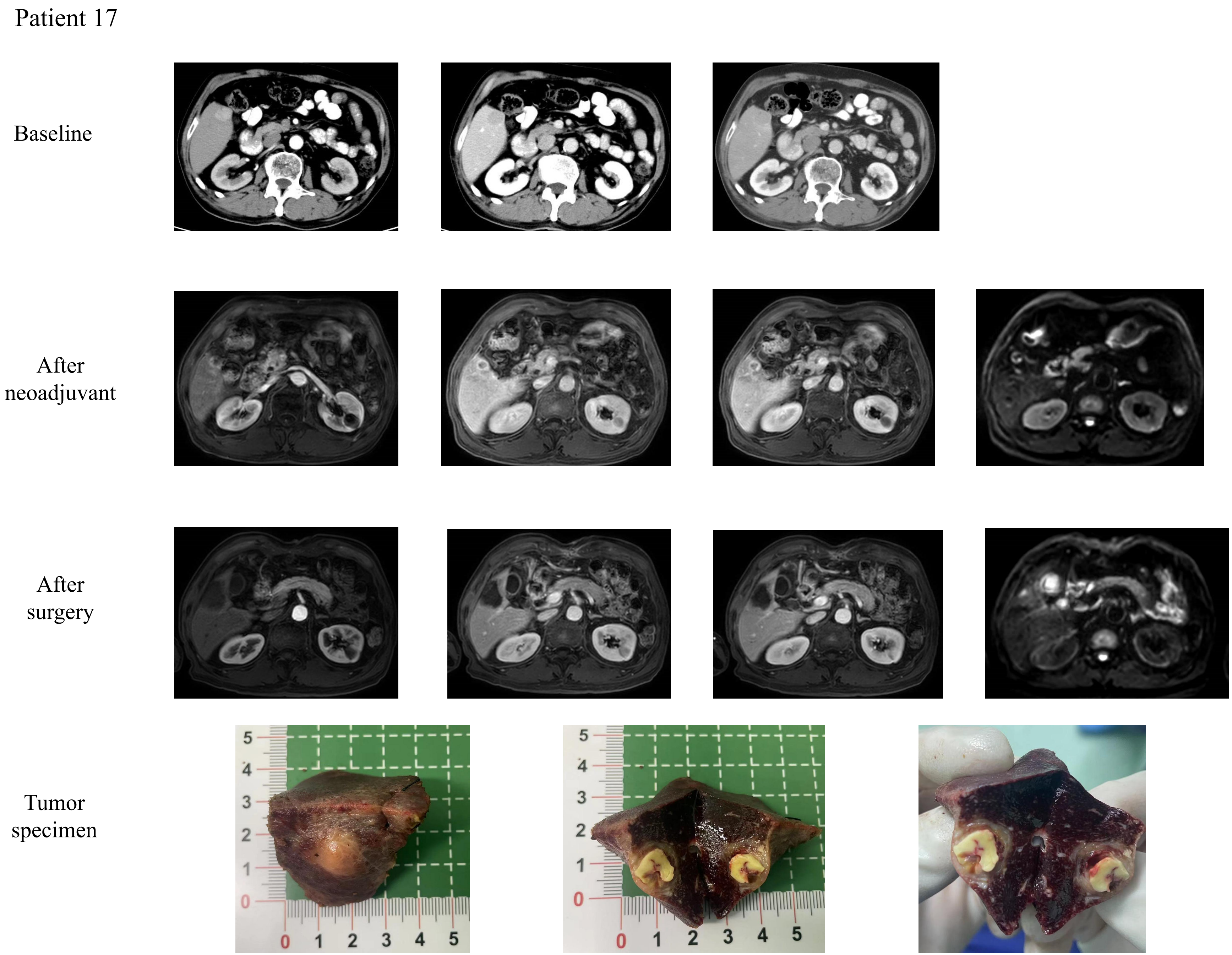

Patient 17
Baseline
After neoadjuvant
After surgery
Tumor specimen

Patient 18

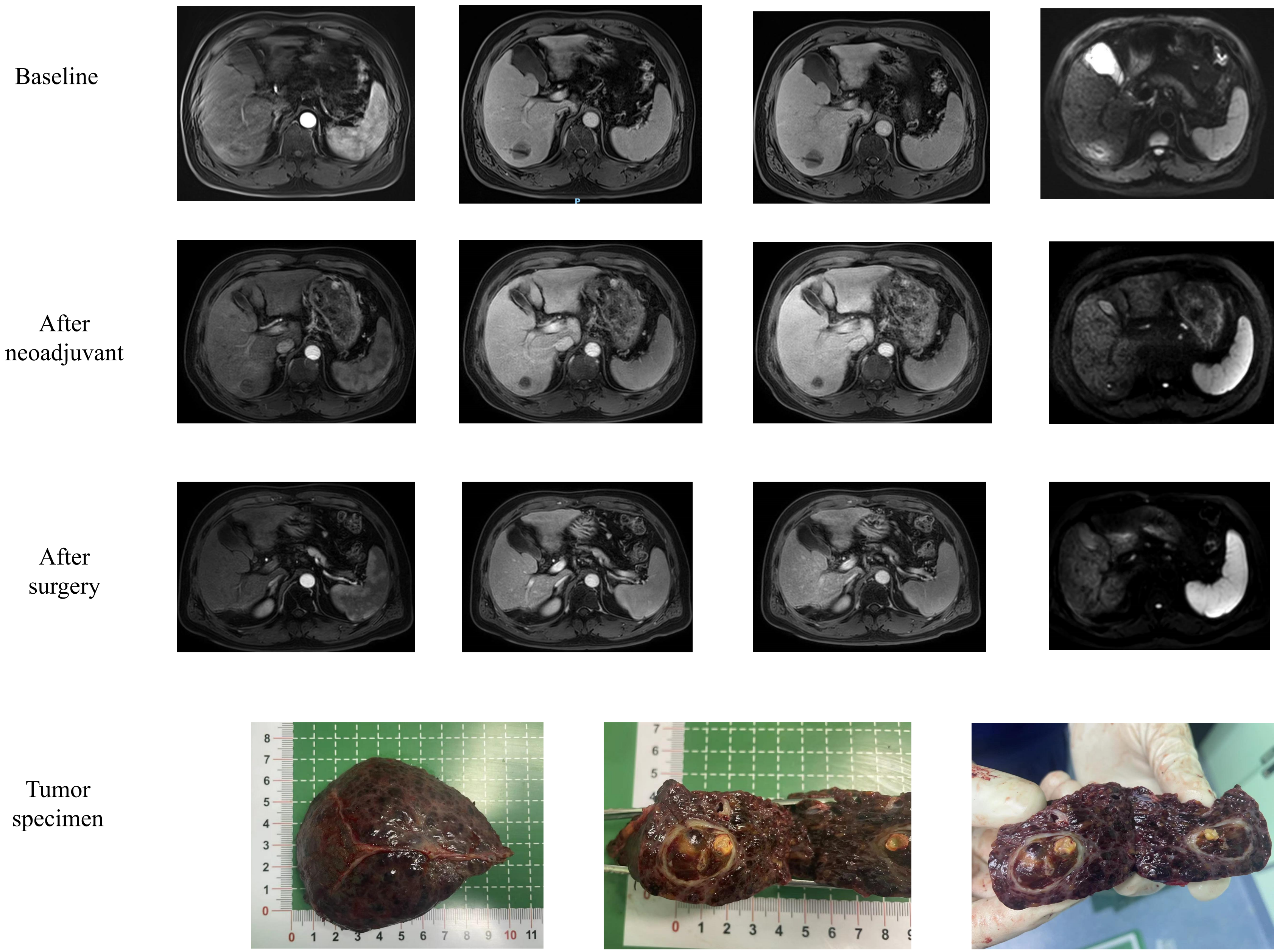

Patient 19

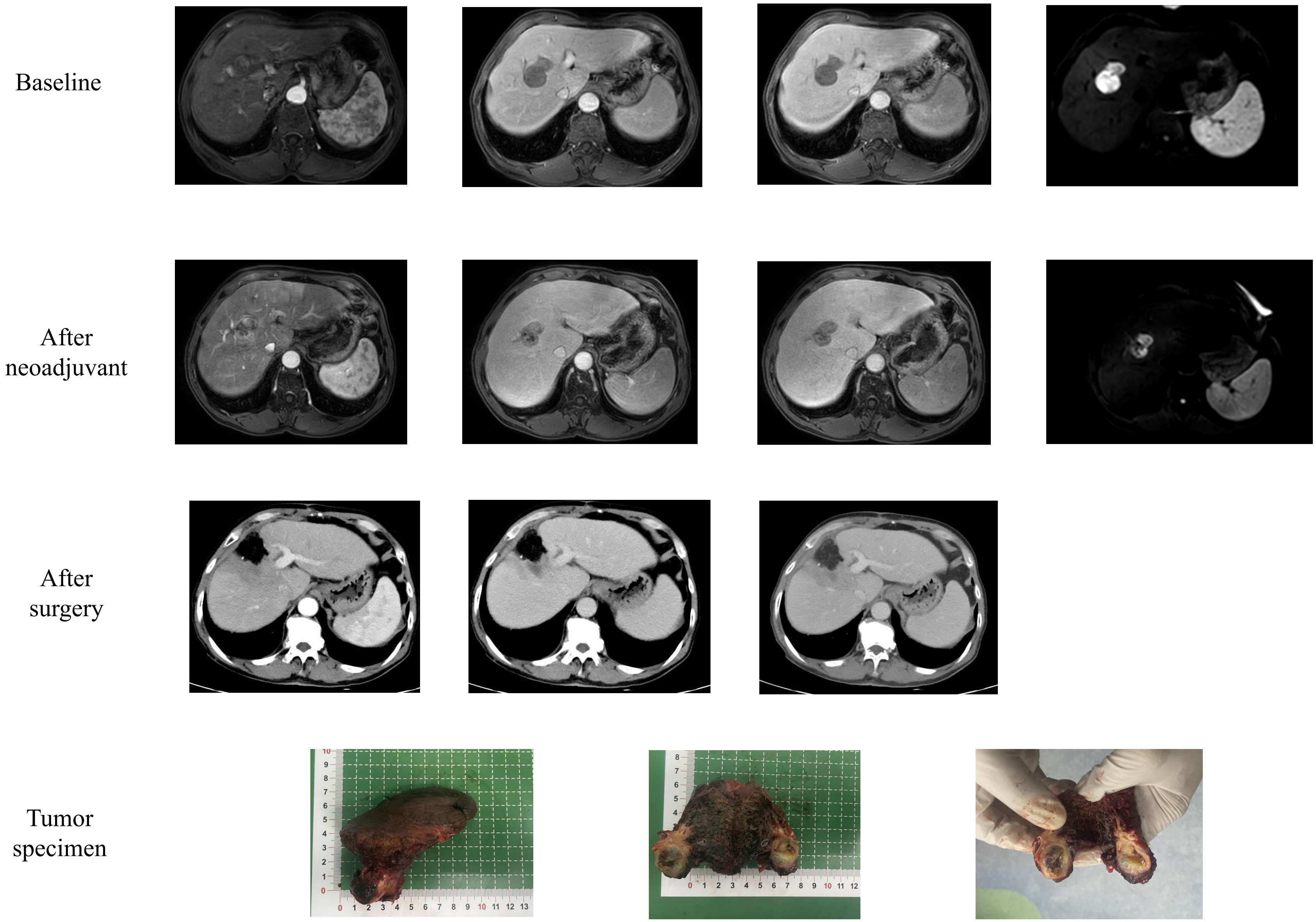

Patient 20

Baseline

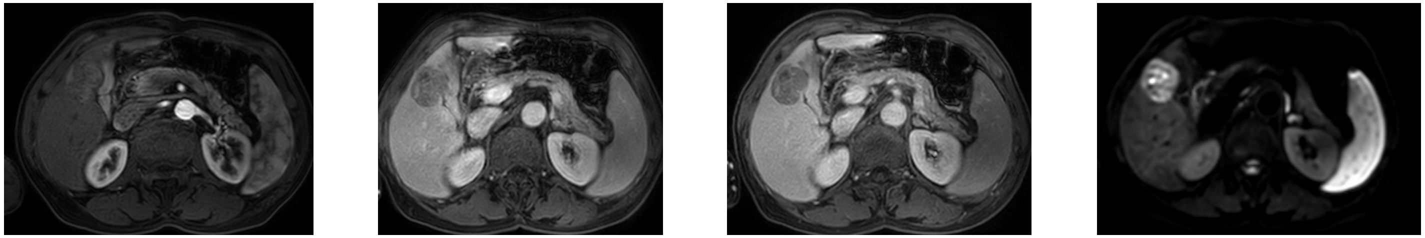

After neoadjuvant

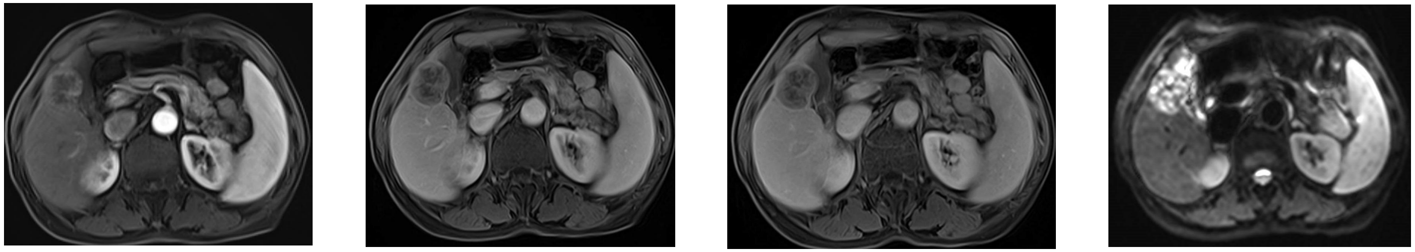

After surgery

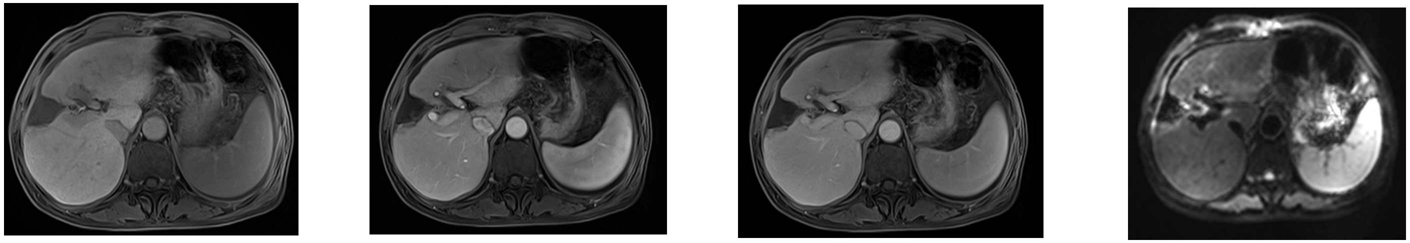

Tumor specimen

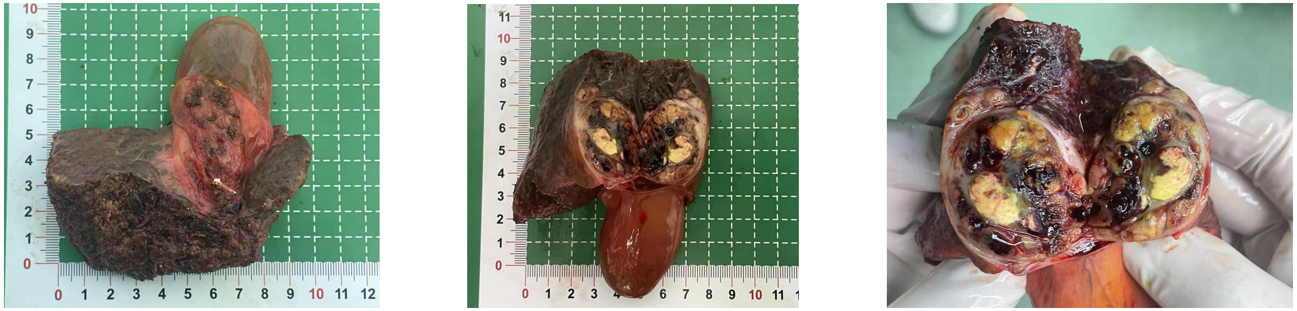

Supplement: Supplementary file 6 — Source Data [file 41467_2024_47420_MOESM6_ESM.zip › Source data_Representative MRICT images.pdf]
