# Supplementary material for: Neoadjuvant tislelizumab plus stereotactic body radiotherapy and adjuvant tislelizumab in early-stage resectable hepatocellular carcinoma: the Notable-HCC phase 1b trial
Source: Nat Commun. 2024 Apr 16;15:3260. doi: 10.1038/s41467-024-47420-3 (PMC11021407; doi:10.1038/s41467-024-47420-3)

SABR to HCC, 24Gy/3Fx，QOD

Patient.1

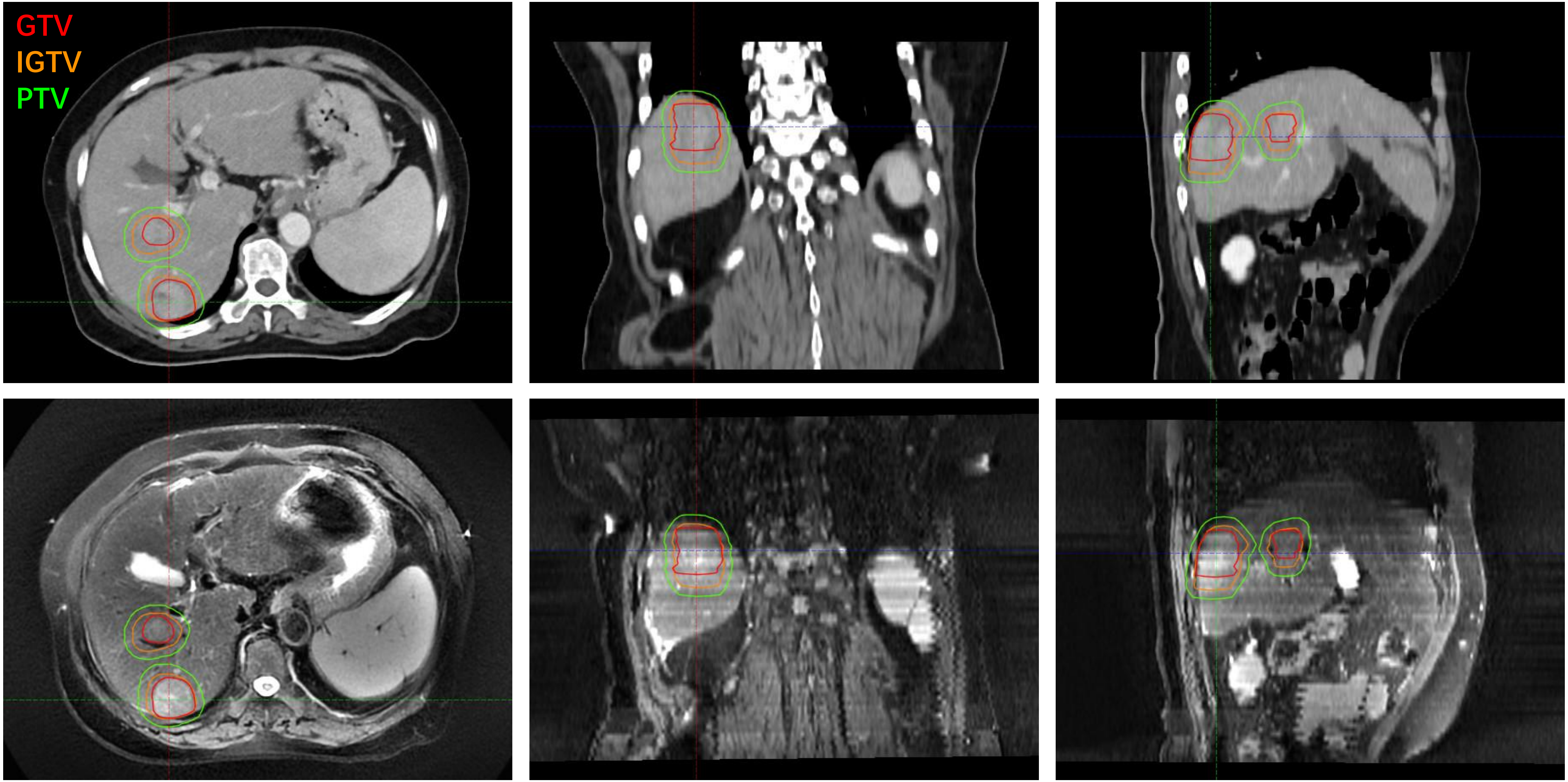

Patient.1

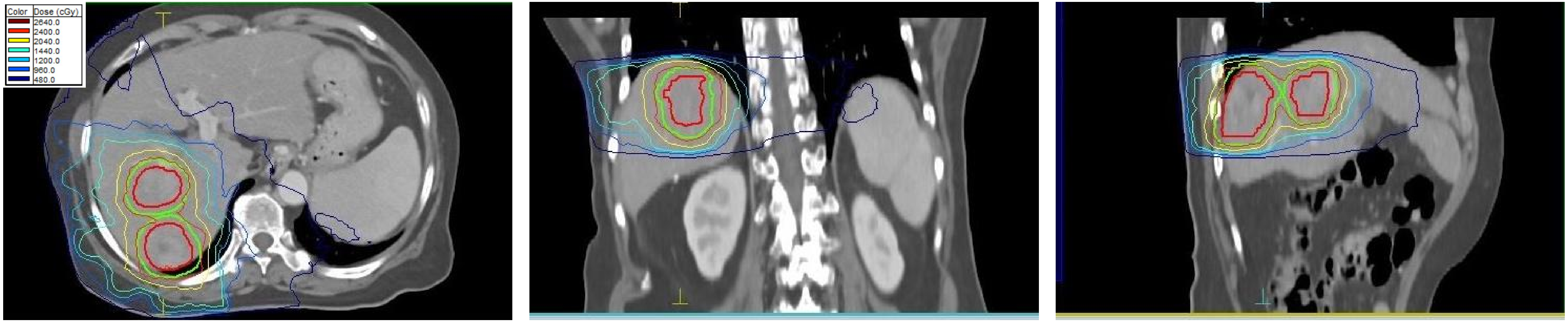

Patient.2

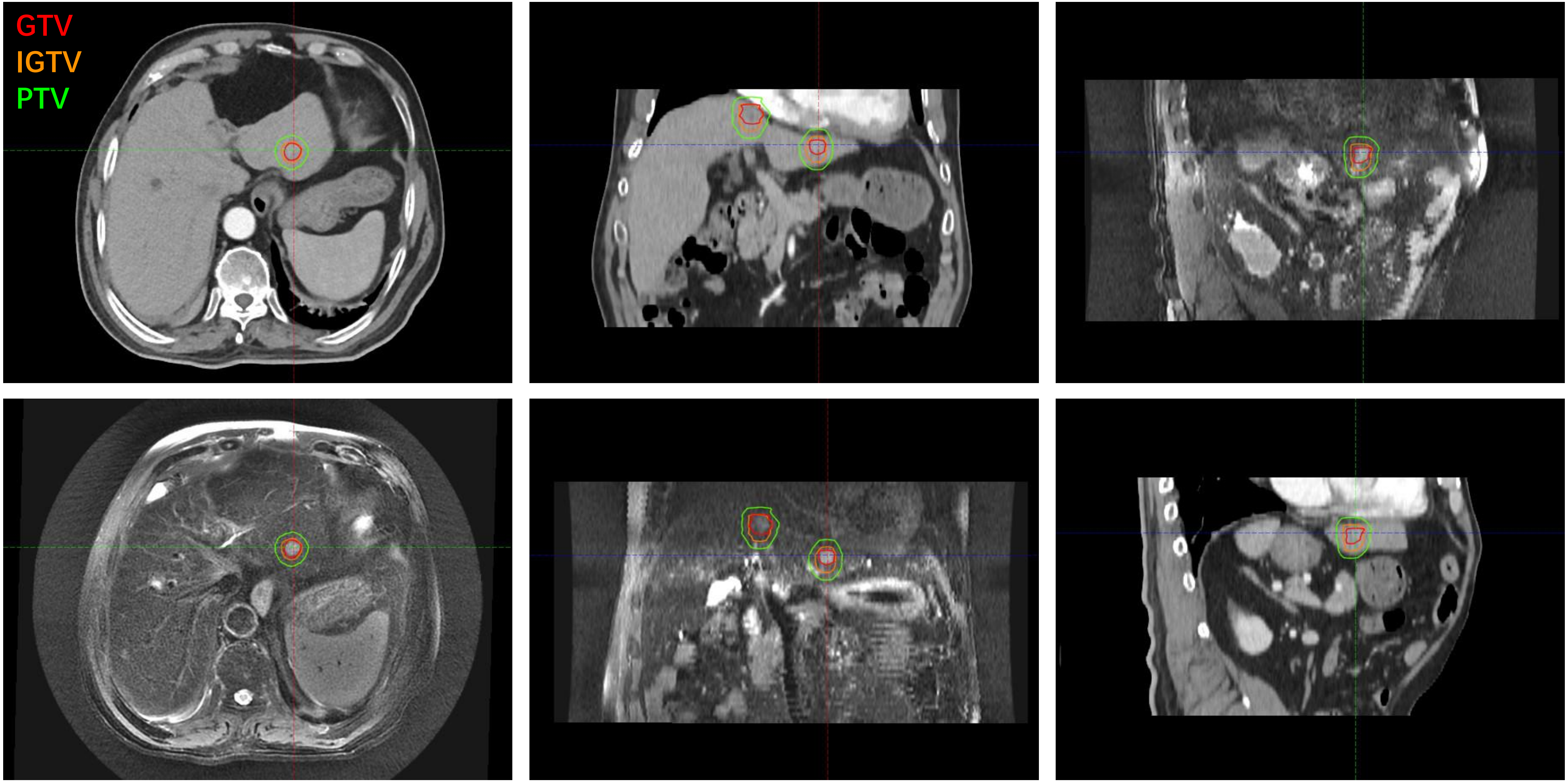

Patient.2

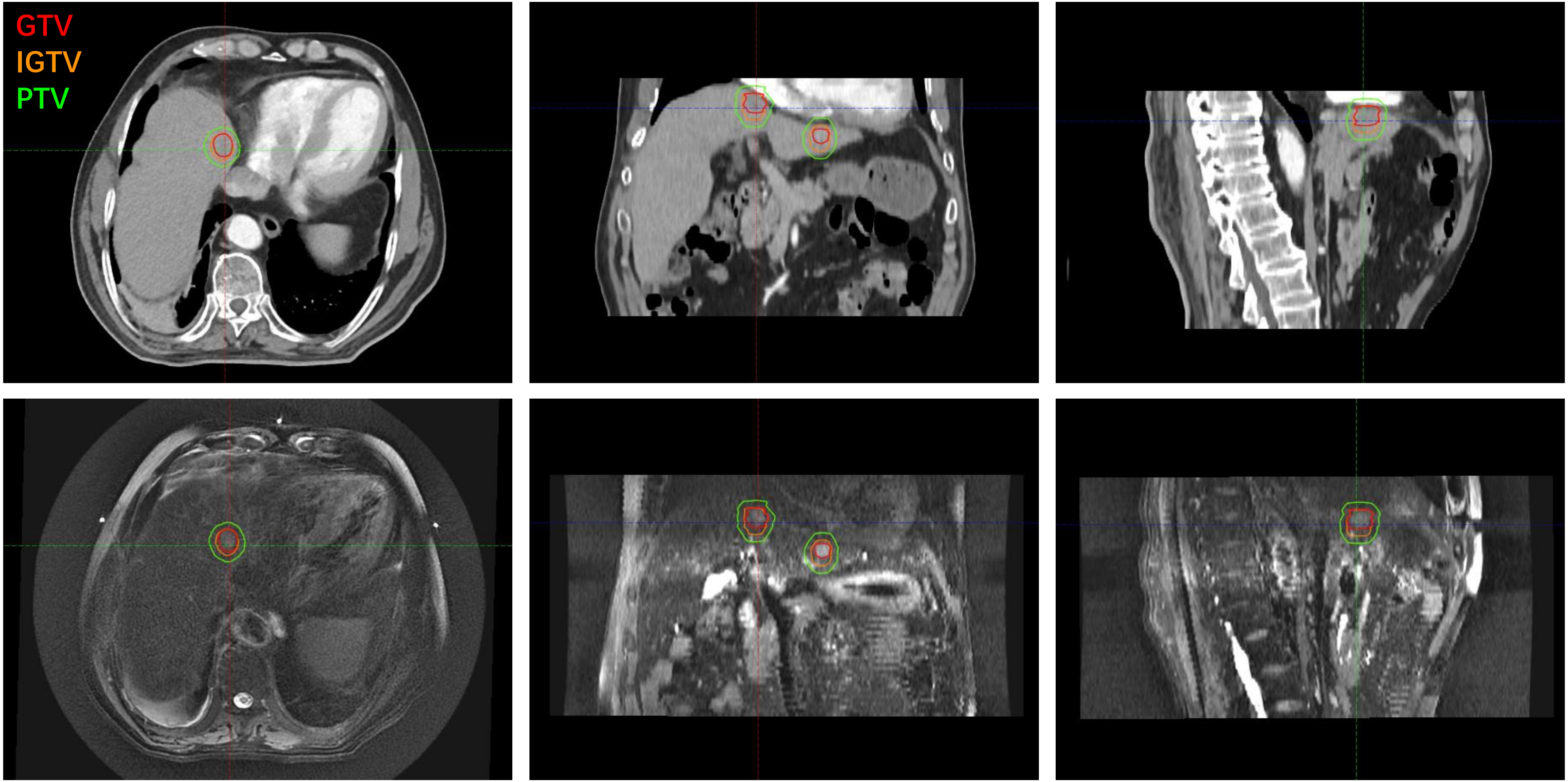

Patient.2

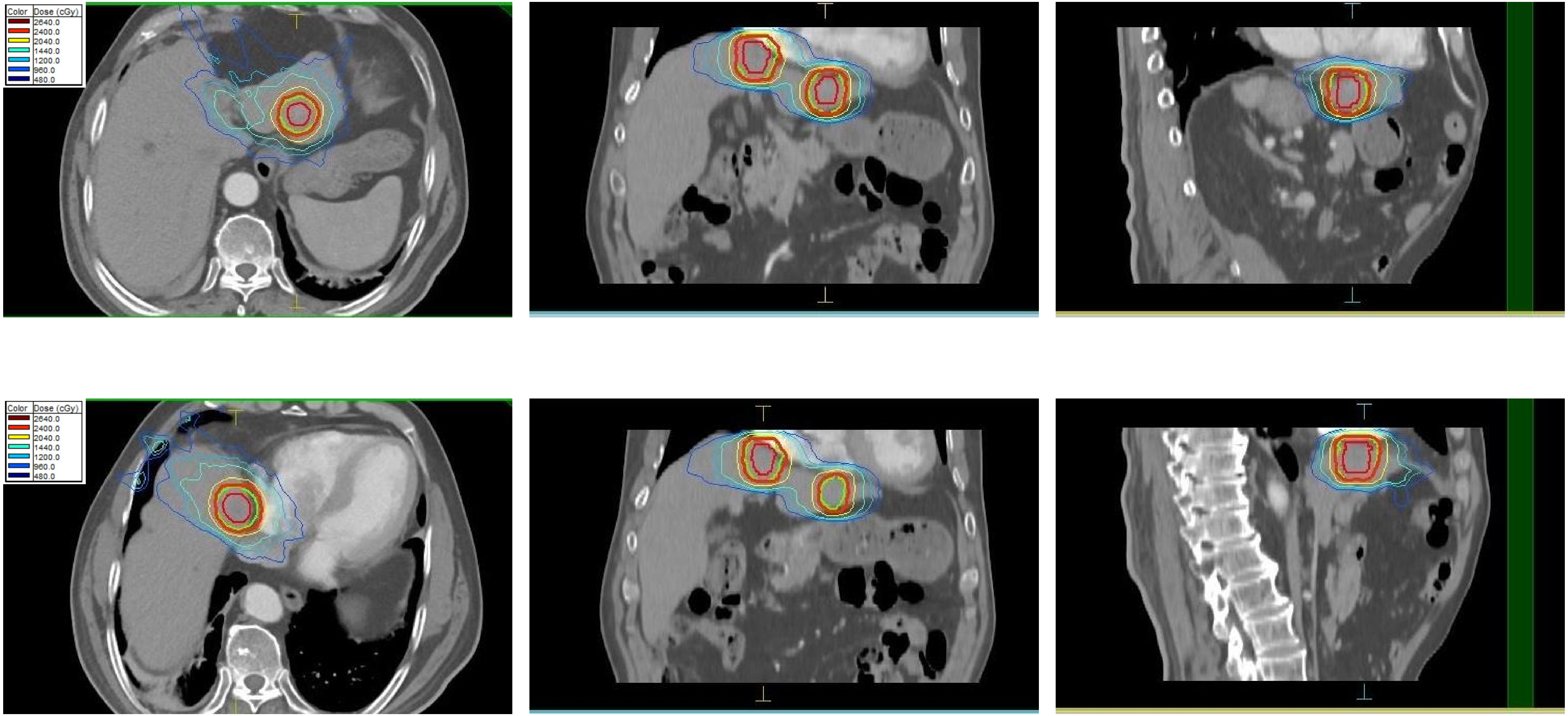
Color Dose (cGy)
2640.0
2400.0
2040.0
1440.0
1200.0
960.0
480.0
Color Dose (cGy)
2640.0
2400.0
2040.0
1440.0
1200.0
960.0
480.0

Patient.3

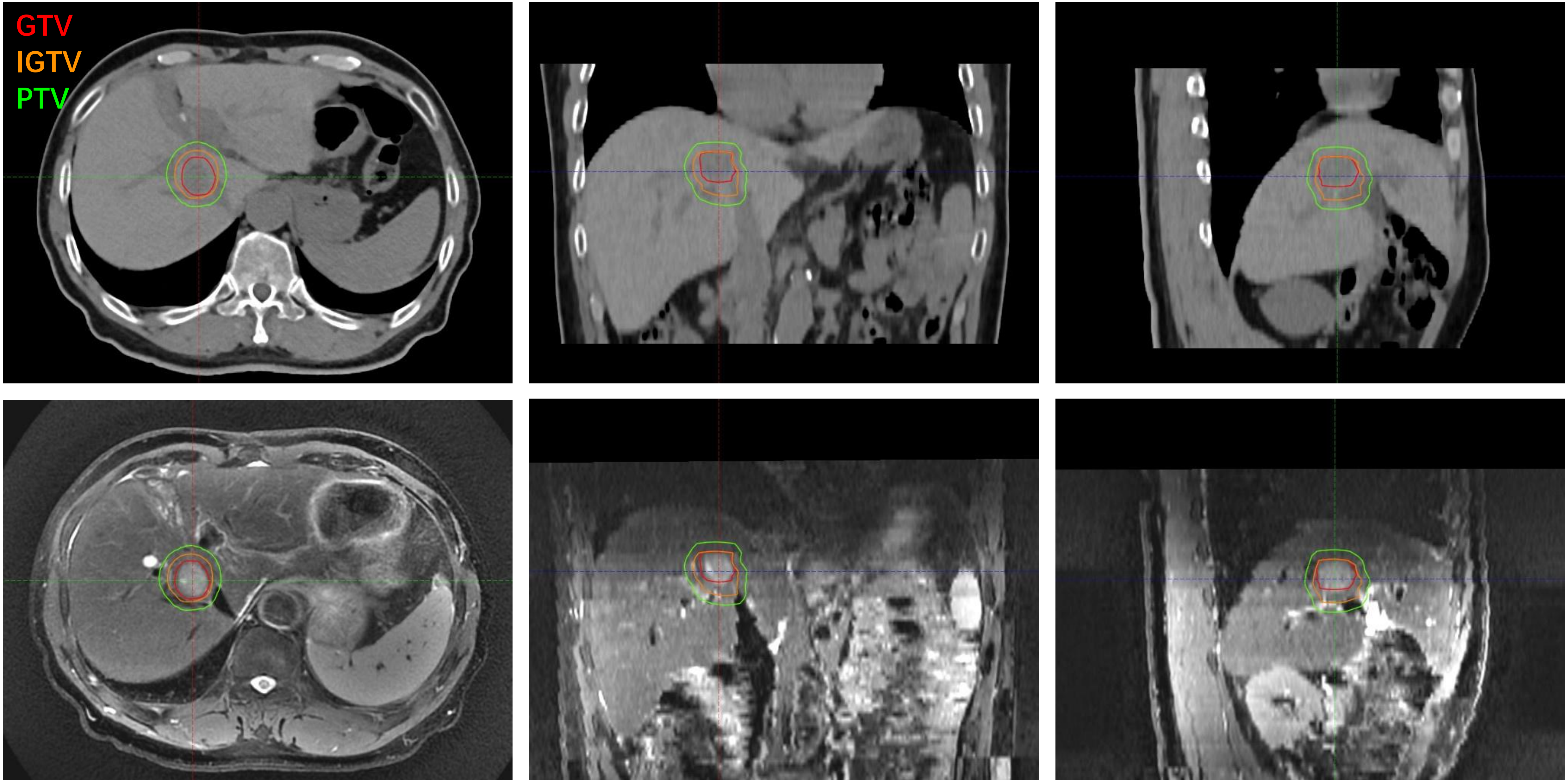

Patient.3

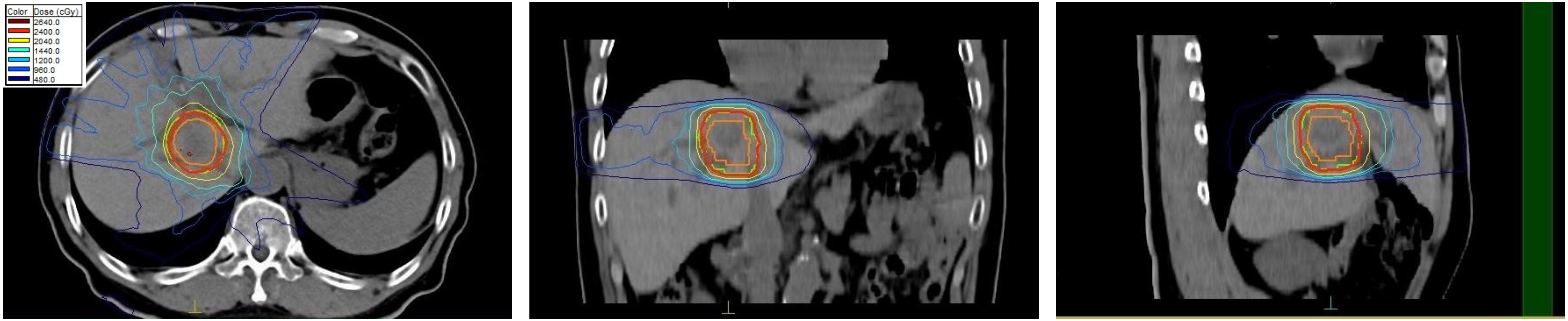

Patient.4

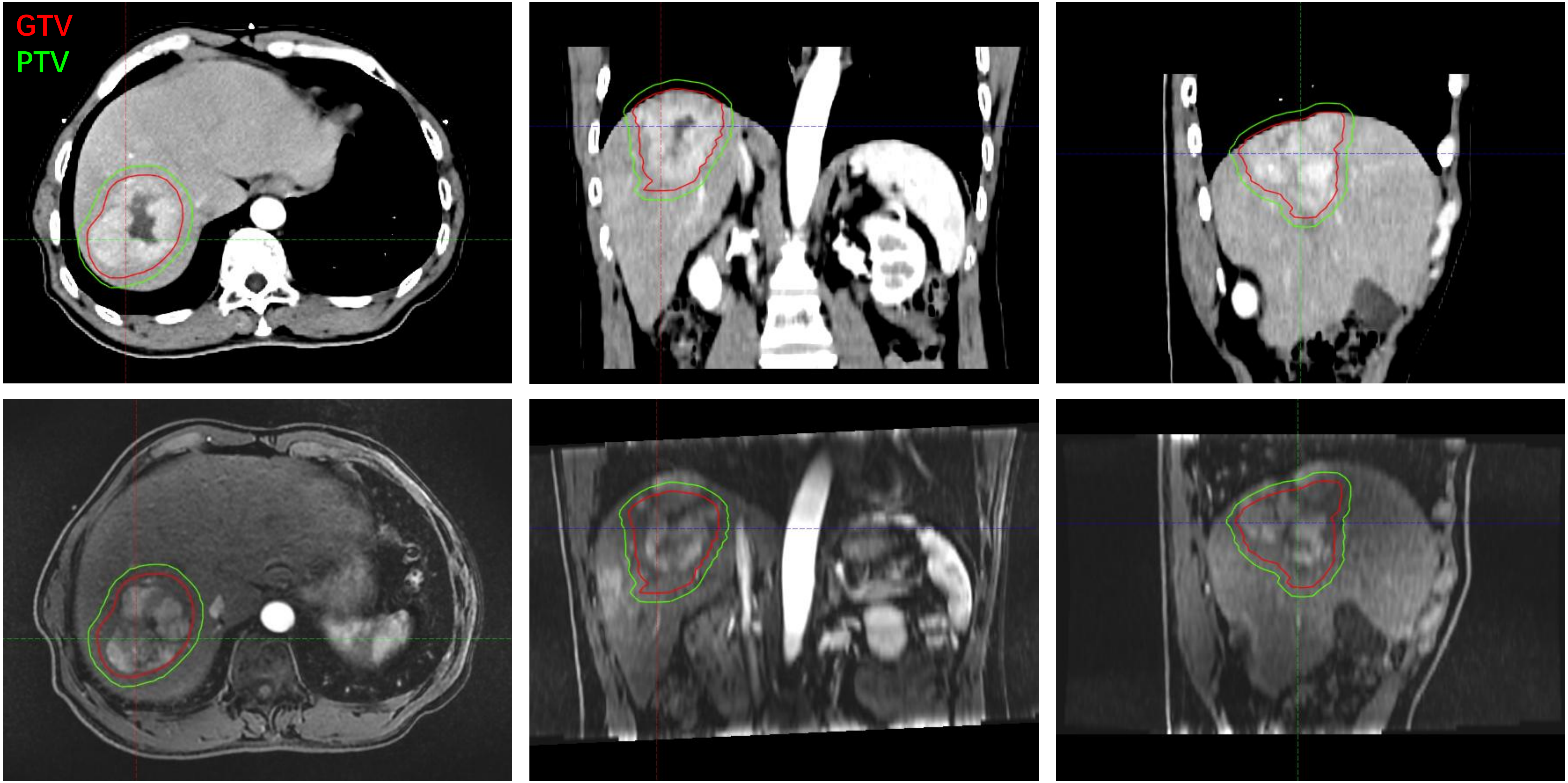

Patient.4

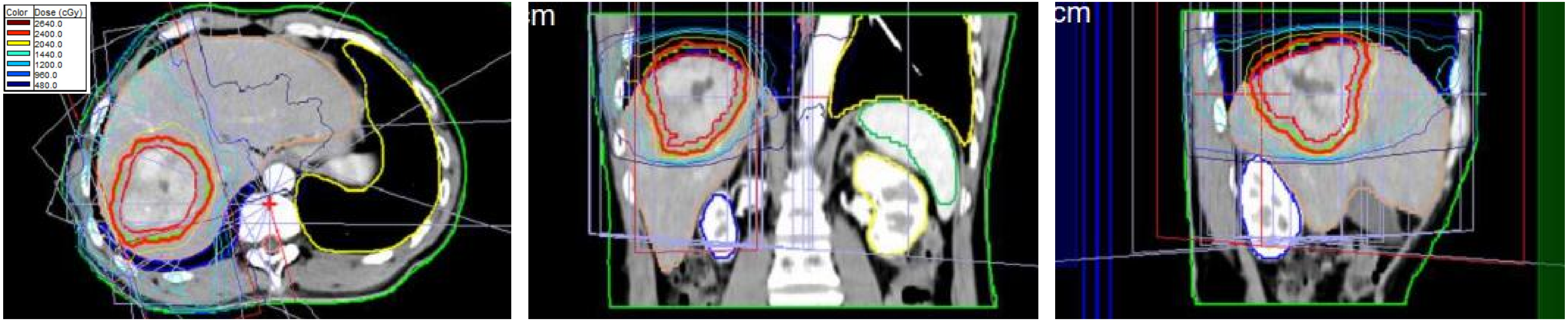

Patient.5

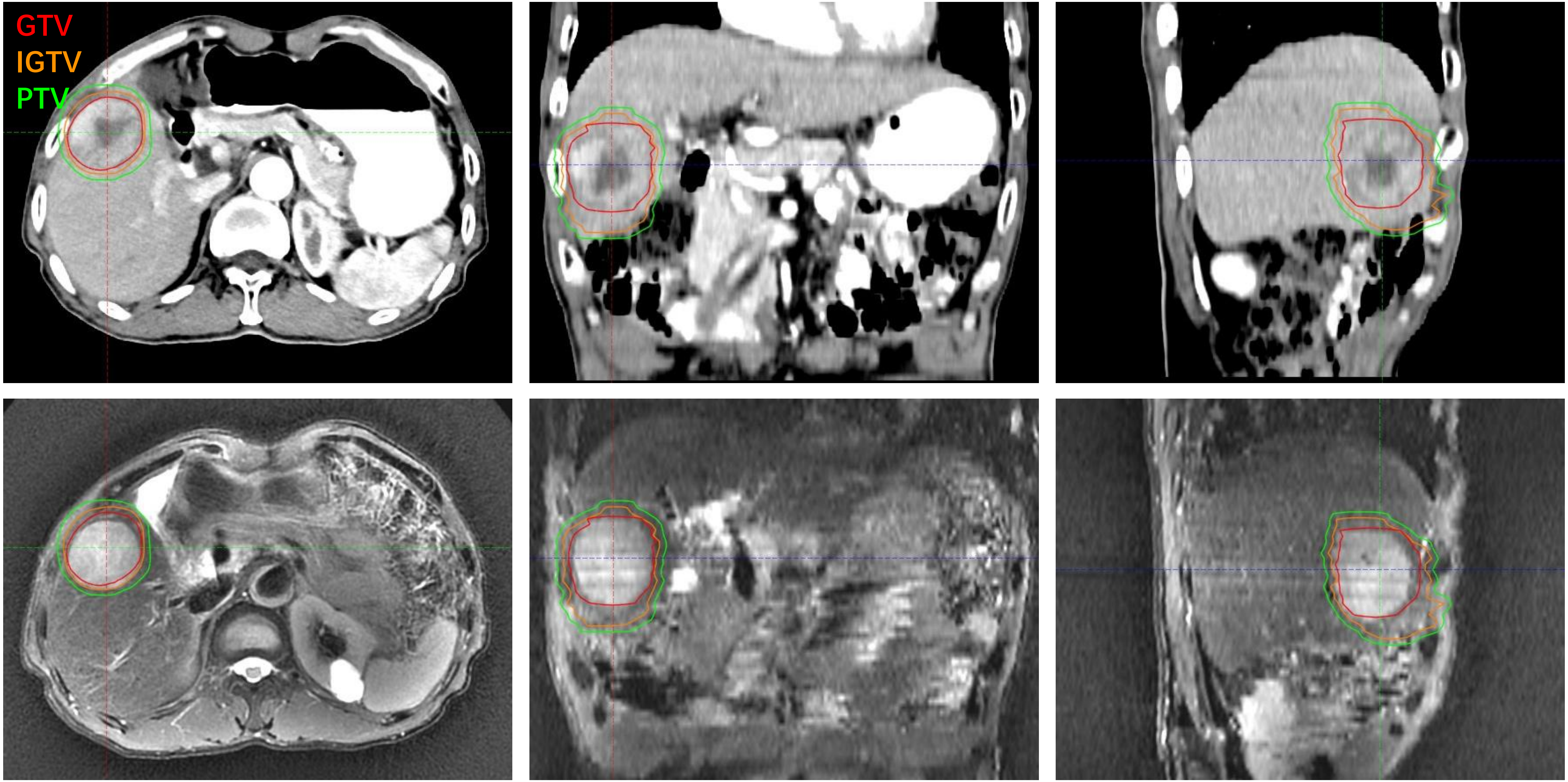

Patient.5

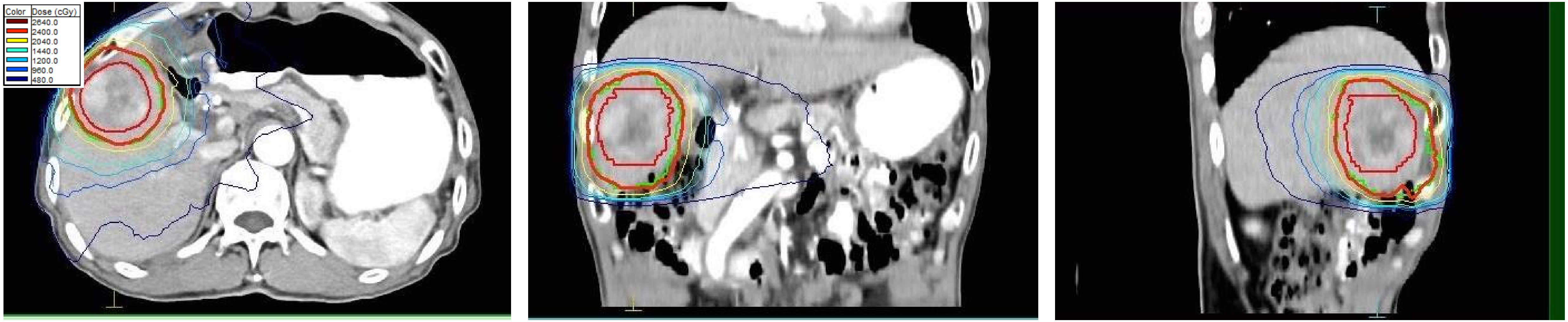

Patient.6

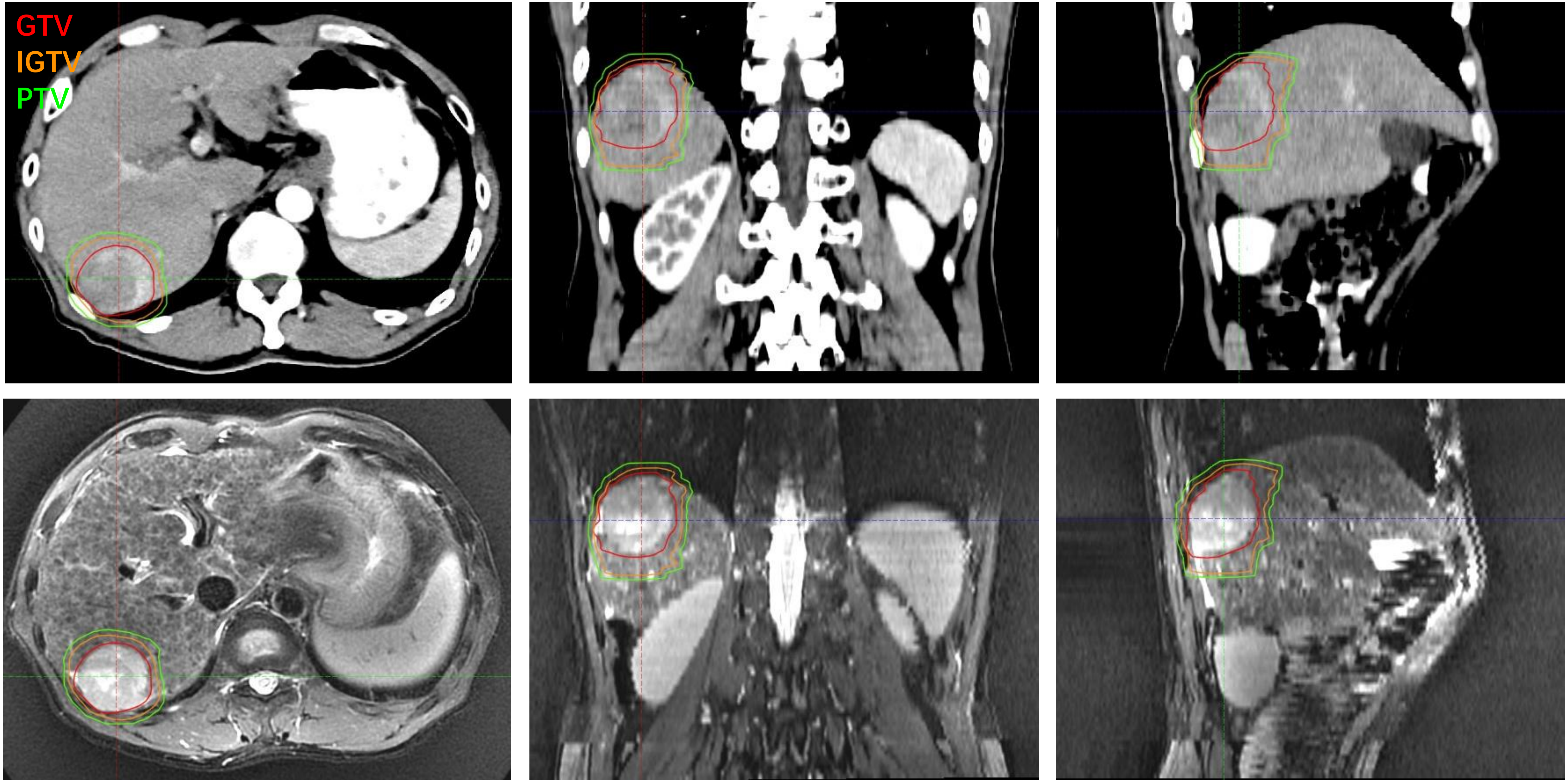

Patient.6

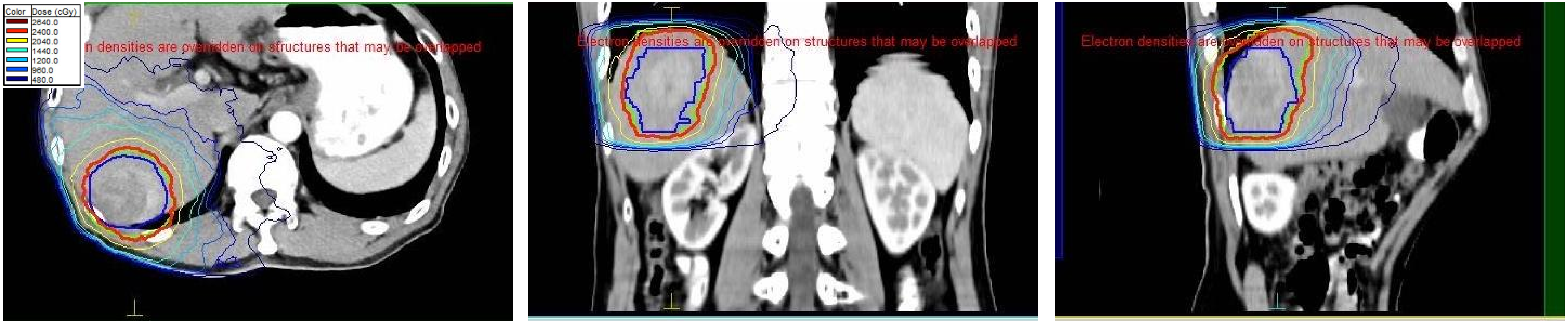
Color
Dose (cGy)
2640.0
2400.0
2040.0
1440.0
1200.0
960.0
480.0
Electron densities are overridden on structures that may be overlapped

Patient.7

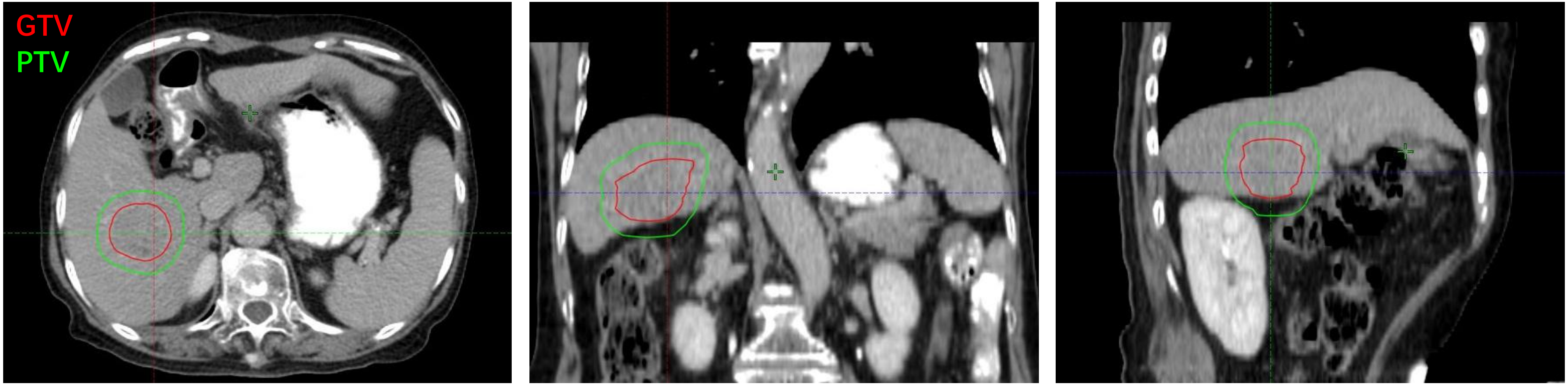

Patient.7

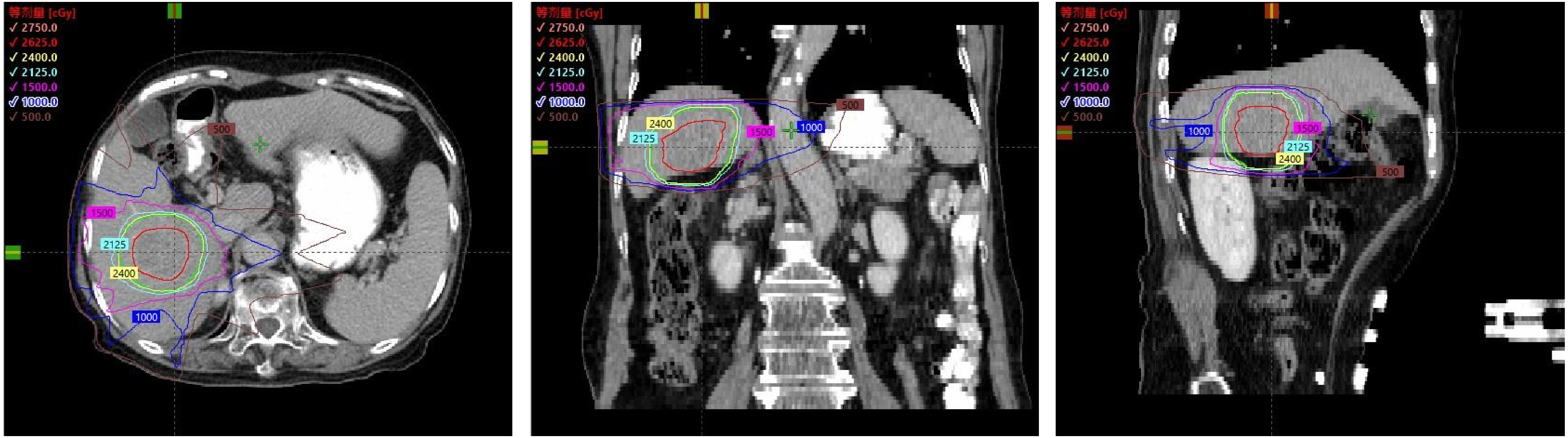
等剂量 [cGy]
2750.0
2625.0
2400.0
2125.0
1500.0
1000.0
500.0
500
1500
2125
2400
1000
等剂量 [cGy]
2750.0
2625.0
2400.0
2125.0
1500.0
1000.0
500.0
2400
2125
1500
1000
500
等剂量 [cGy]
2750.0
2625.0
2400.0
2125.0
1500.0
1000.0
500.0
1000
1500
2125
2400
500

Patient.8

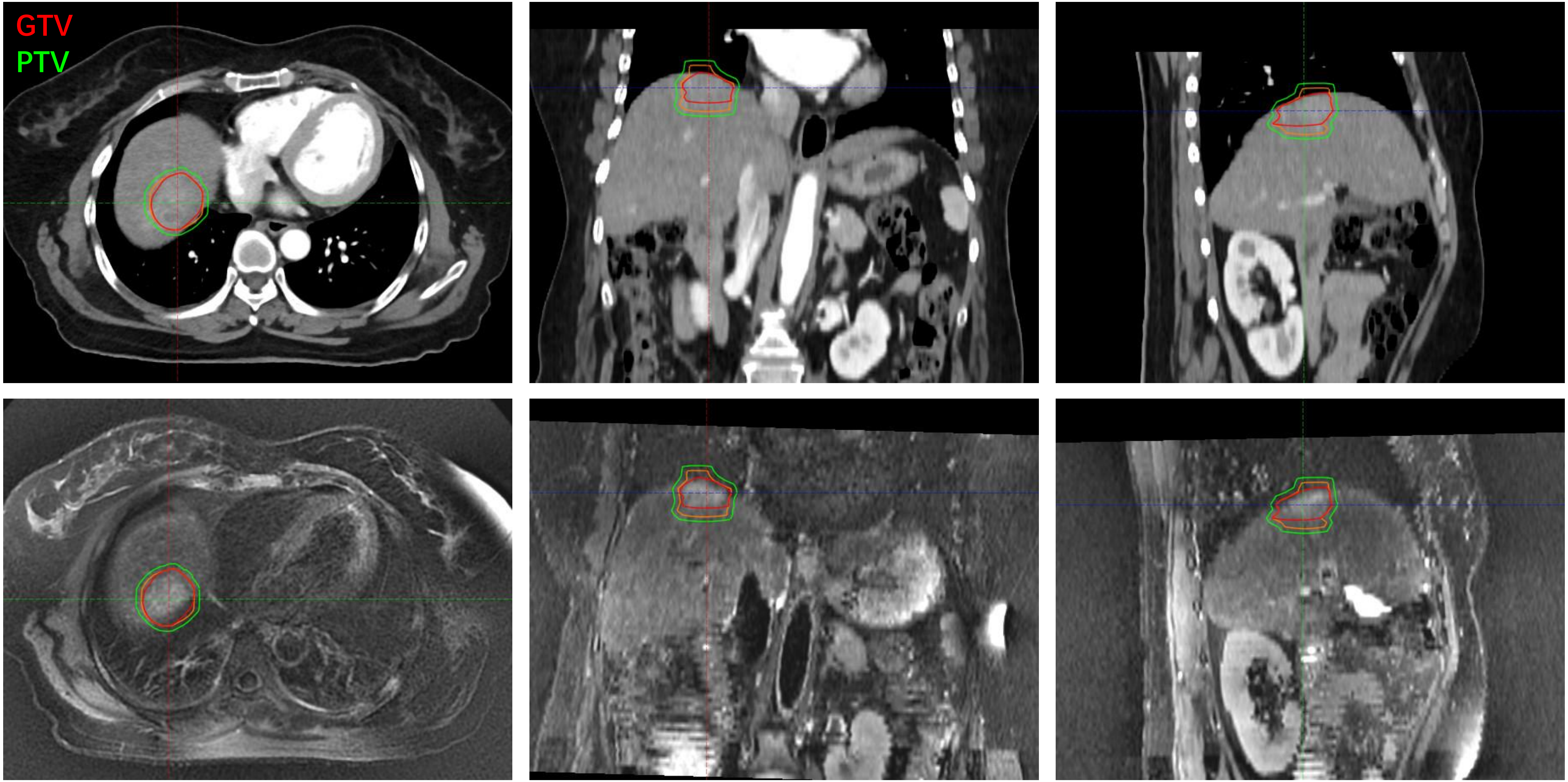

Patient.8

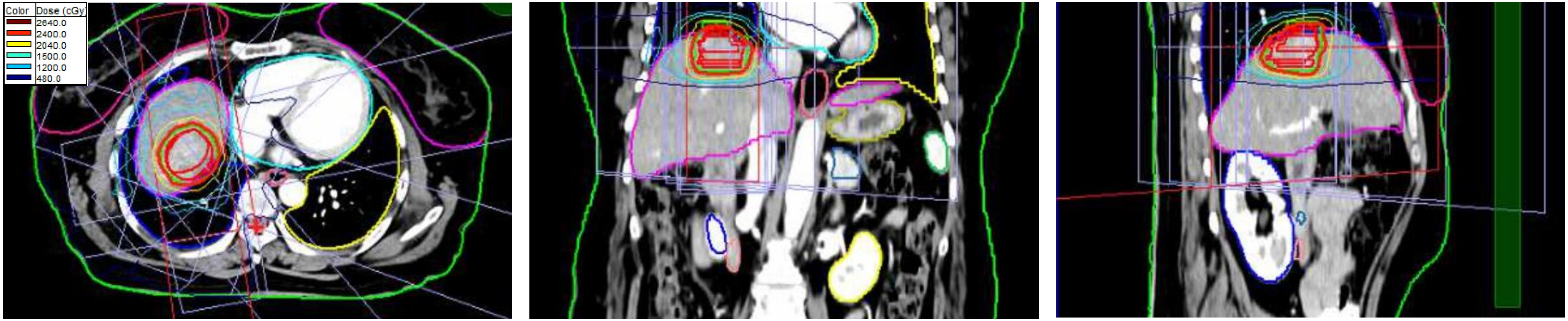

Patient.9

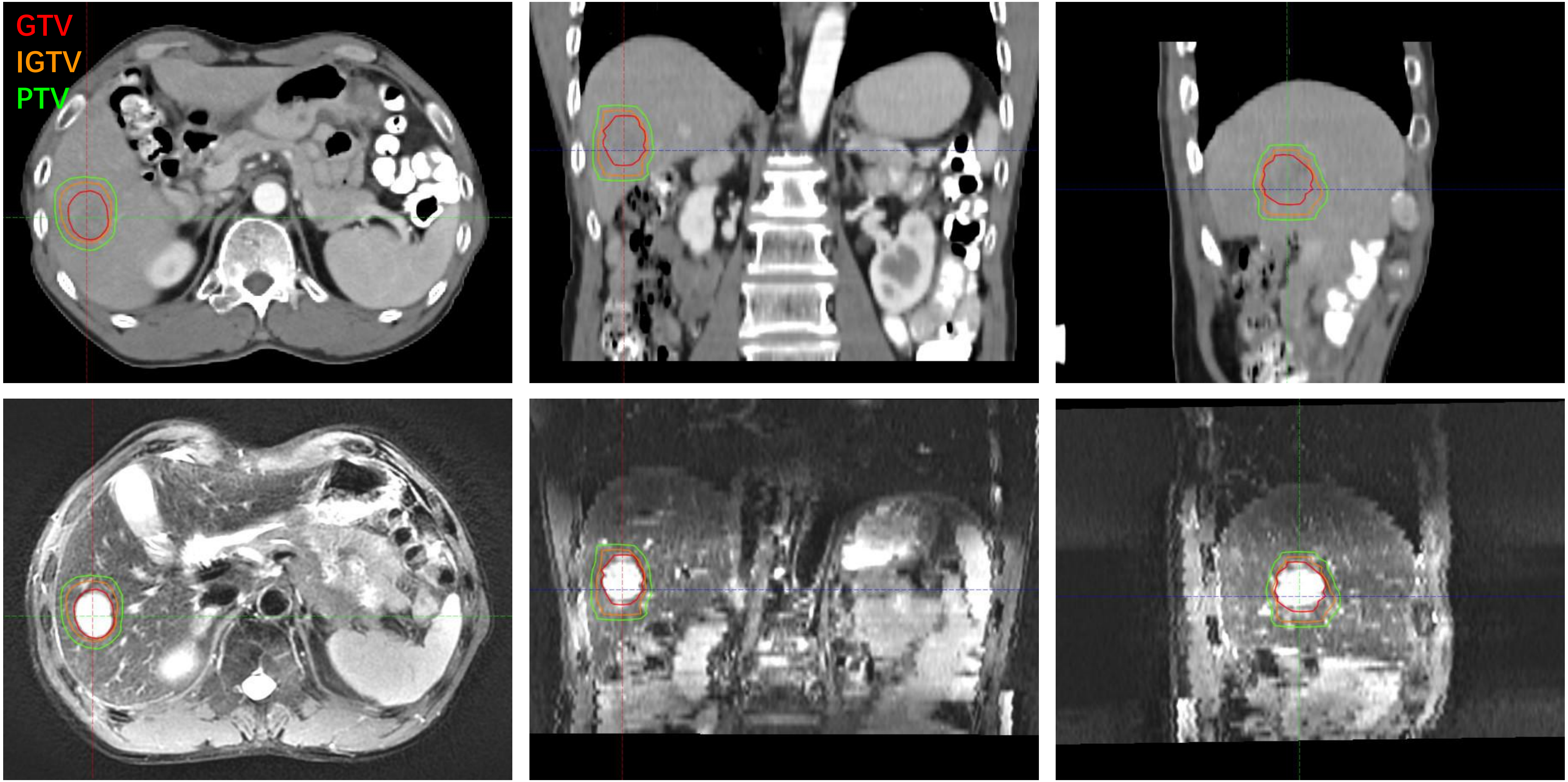

Patient.9

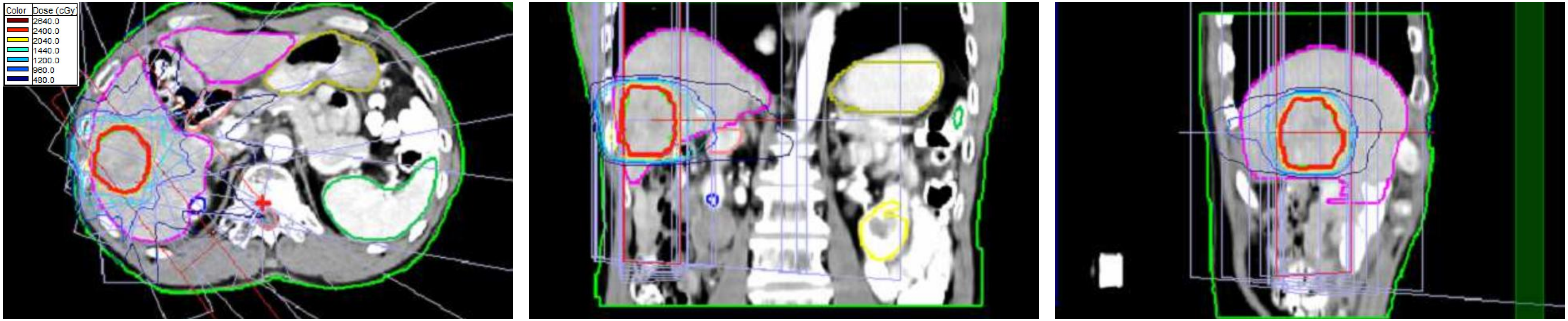

Patient.10

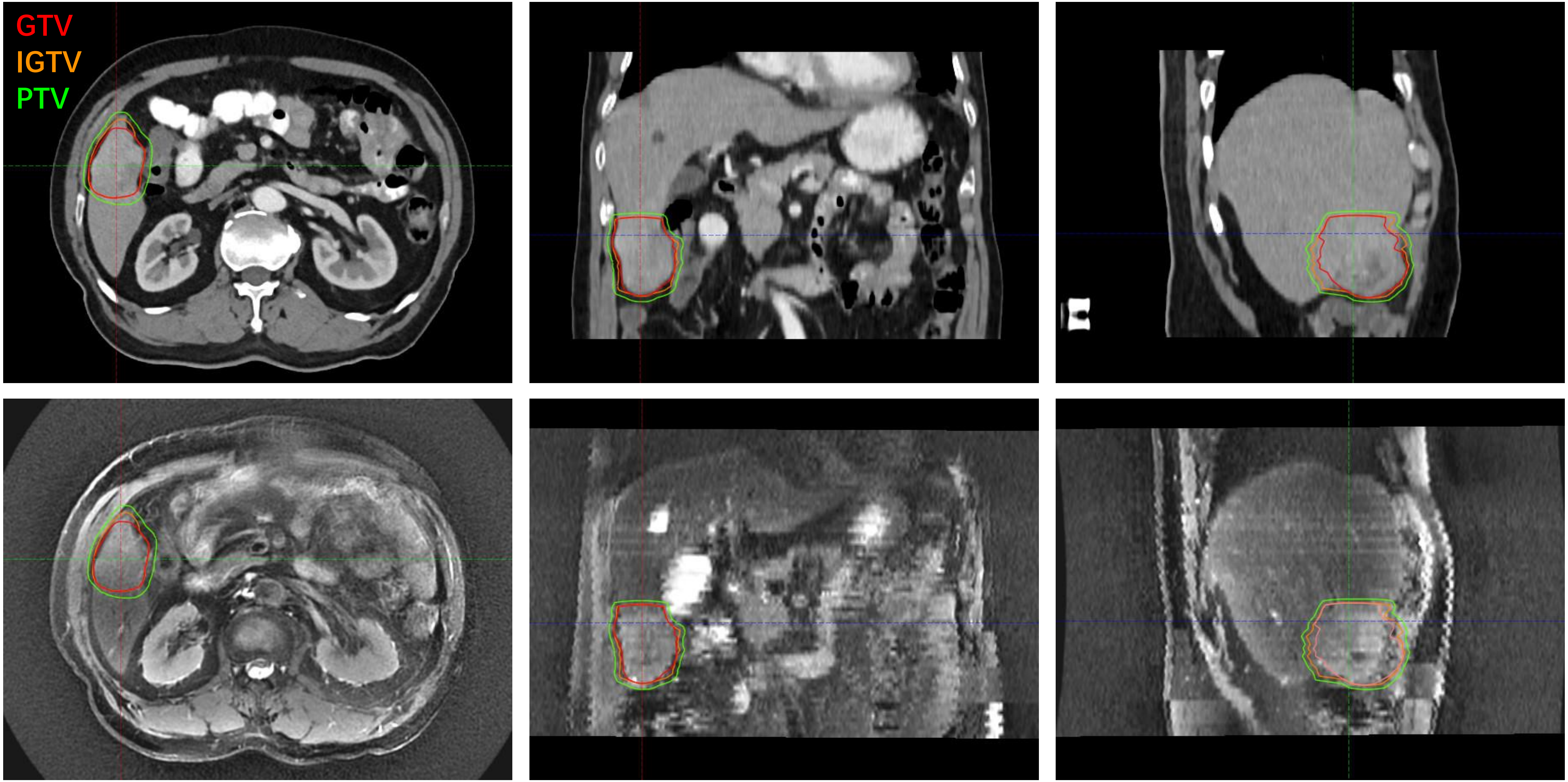

Patient.10

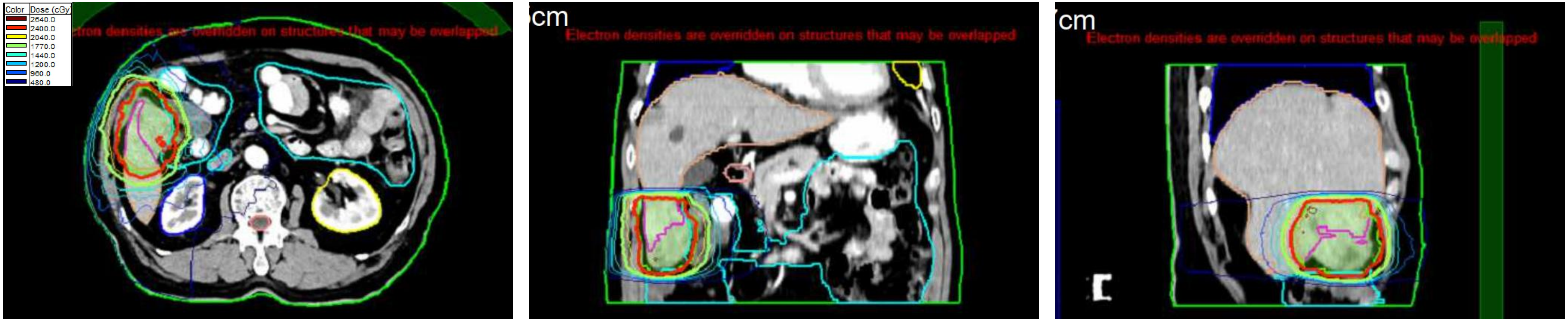
Color
Dose (cGy)
2640.0
2400.0
2040.0
1770.0
1440.0
1200.0
960.0
480.0
Electron densities are overridden on structures that may be overlapped
cm
Electron densities are overridden on structures that may be overlapped
cm

Patient.11

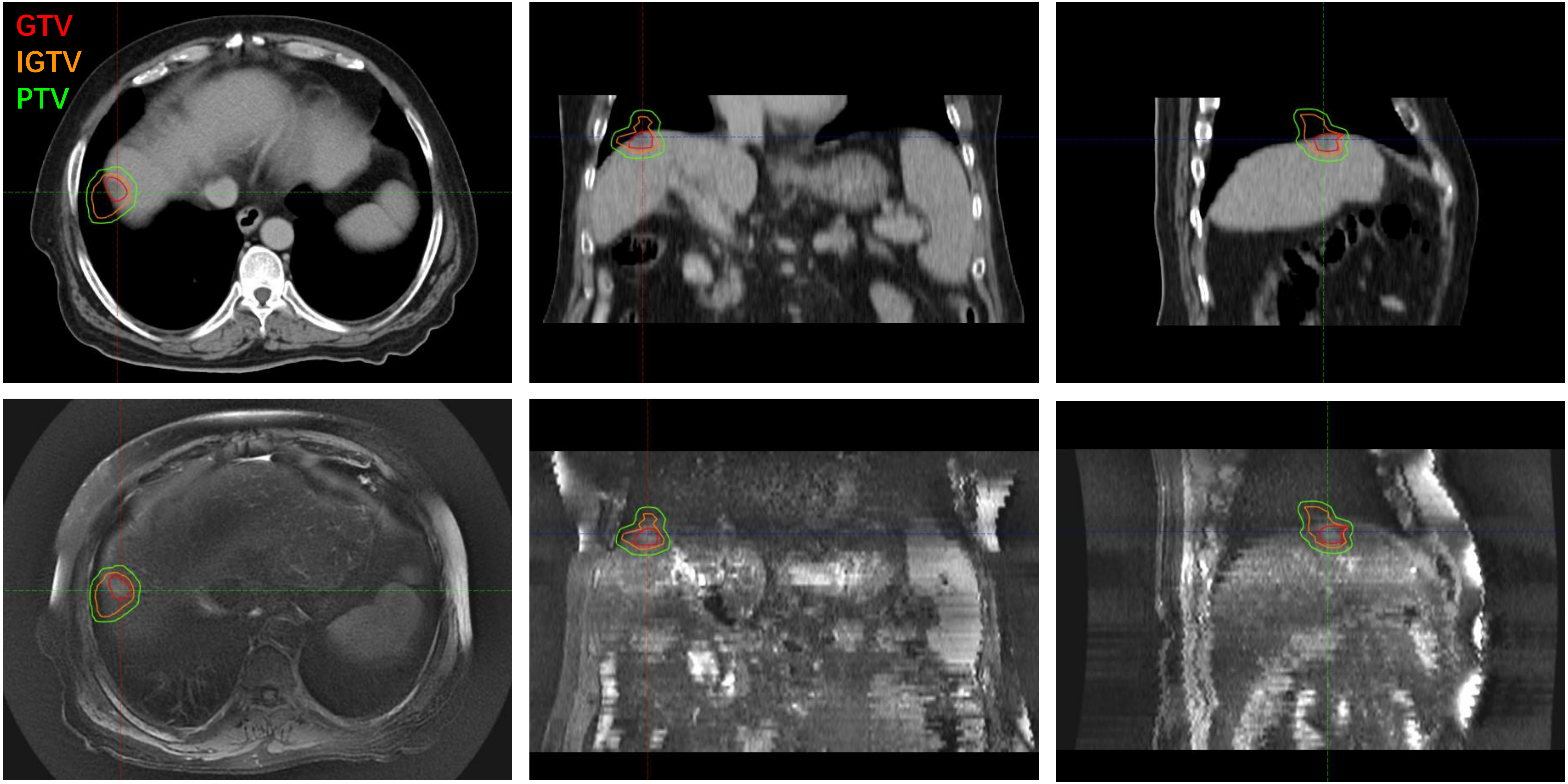

Patient.11

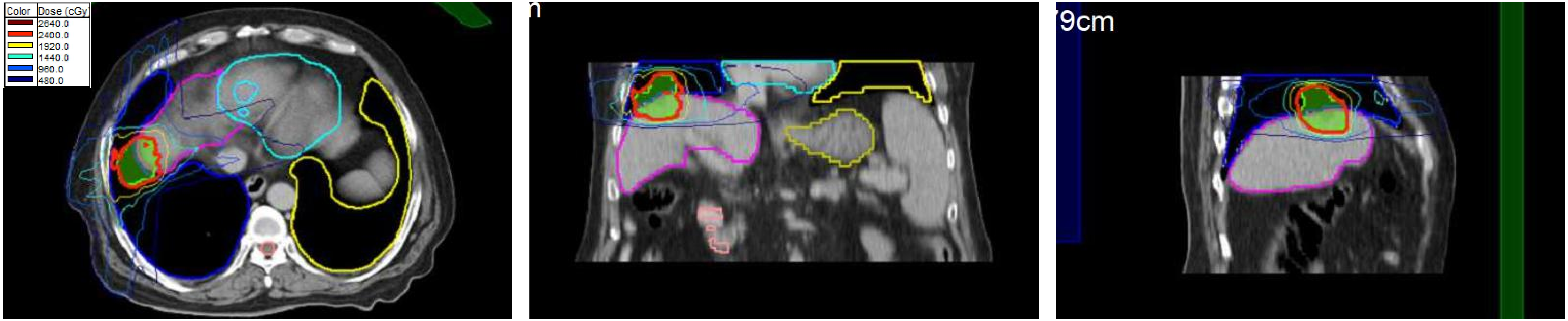

Patient.12

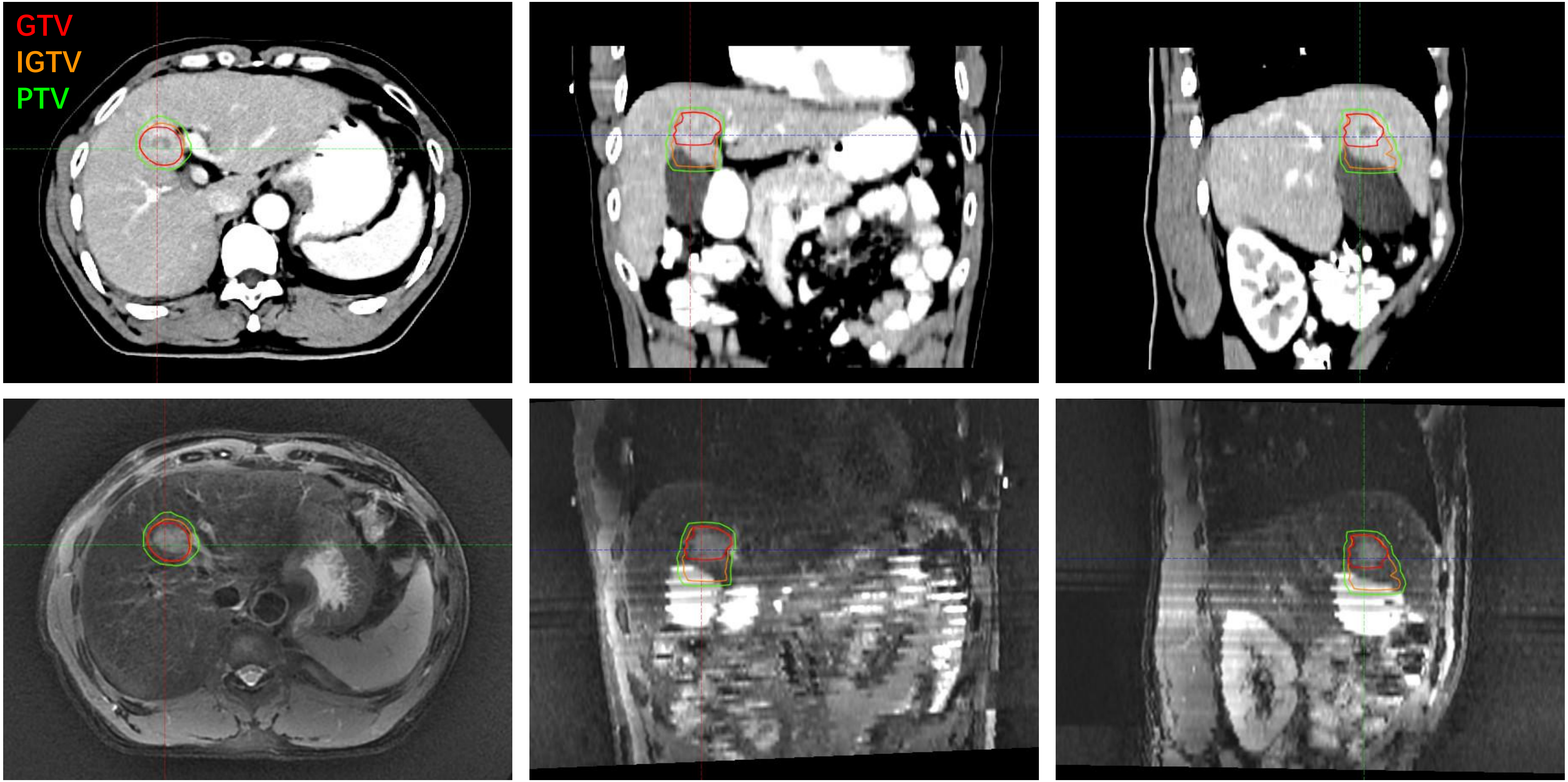

Patient.12

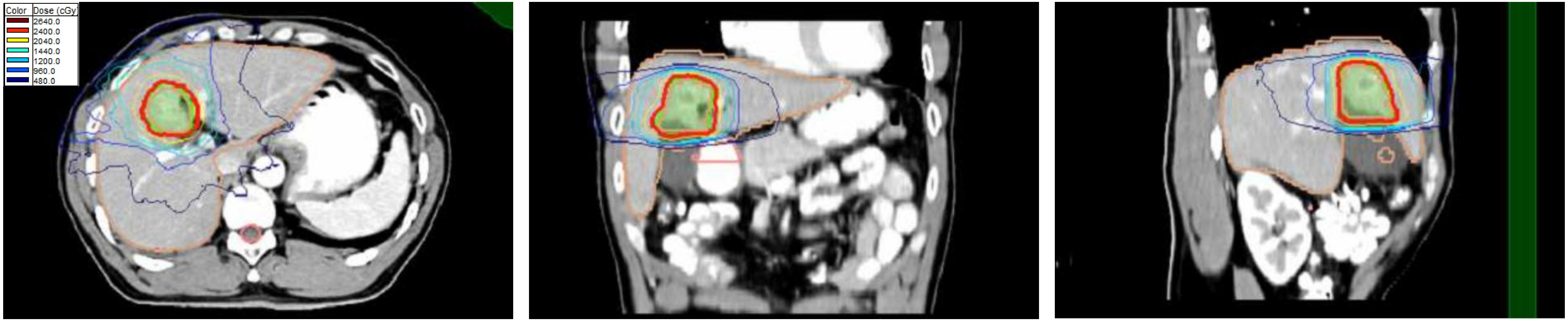

Patient.13

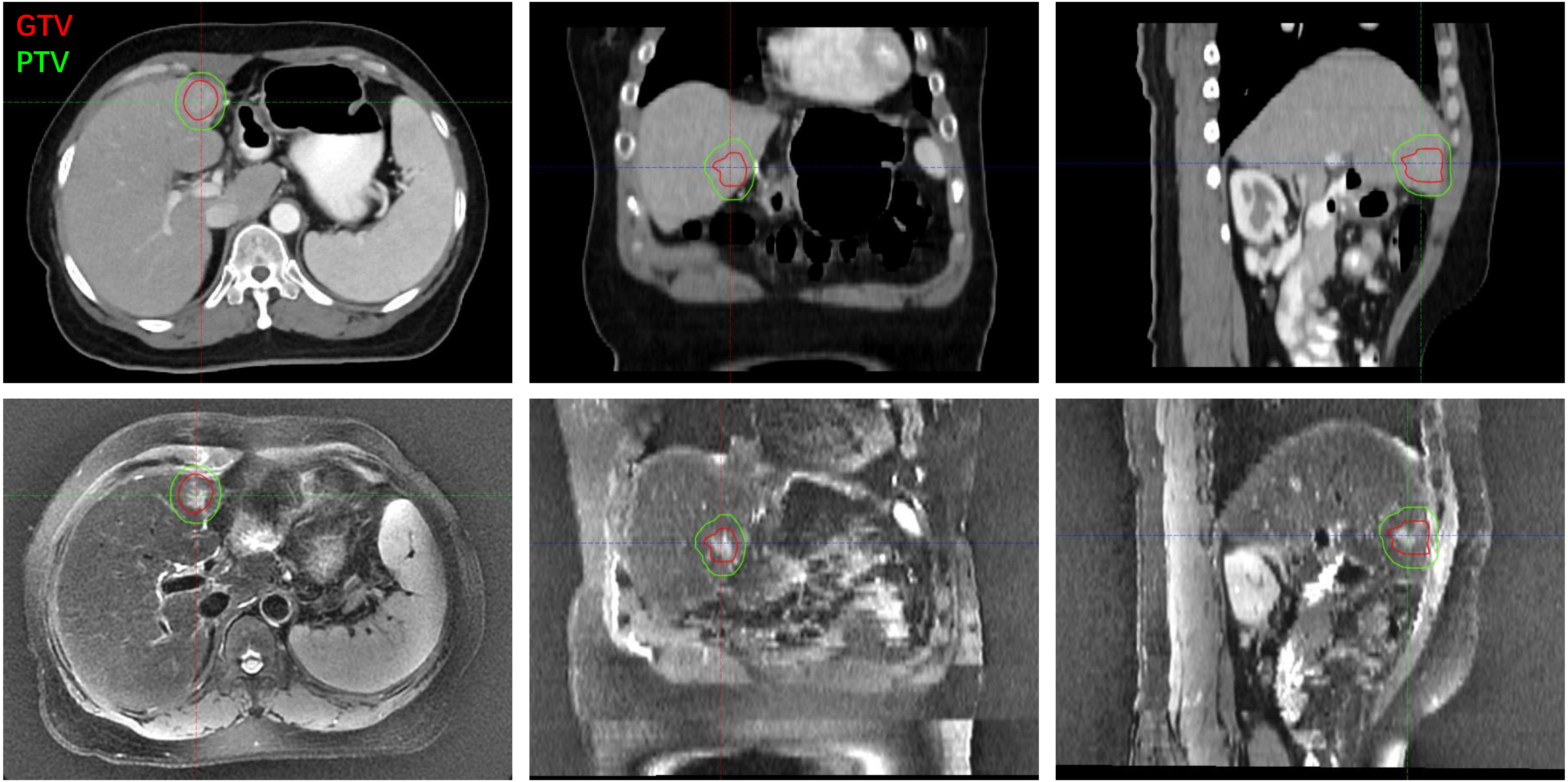

Patient.13

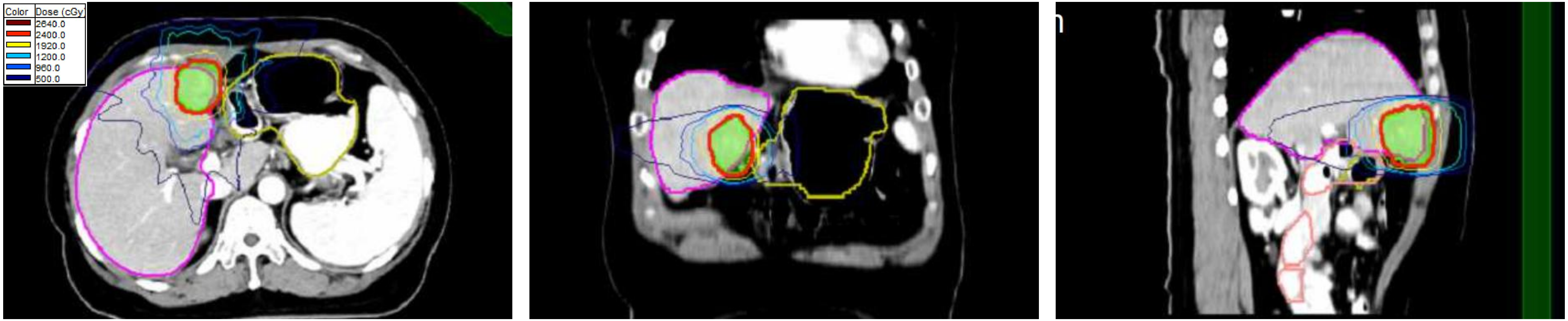

Patient.14

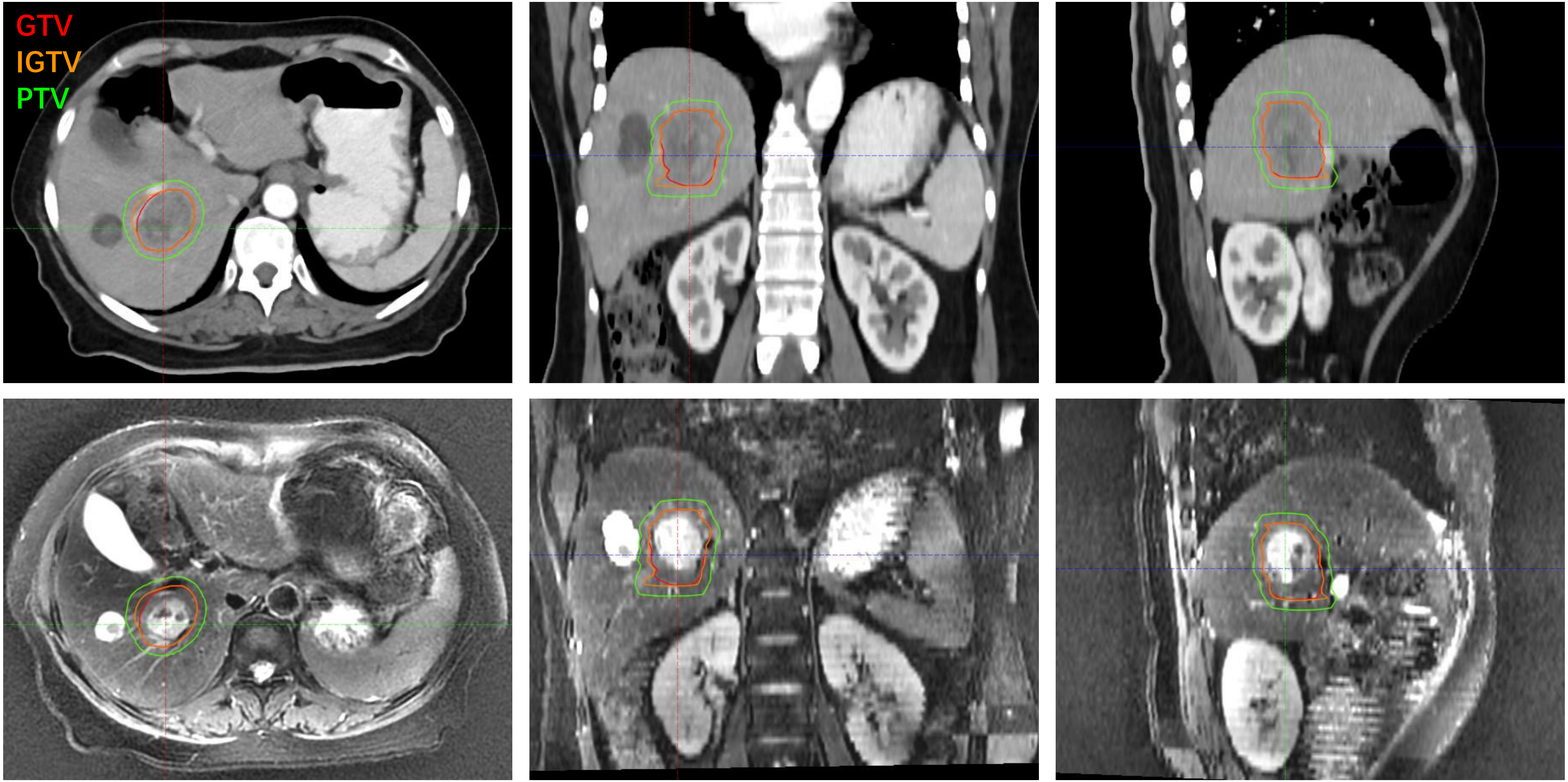

Patient.14

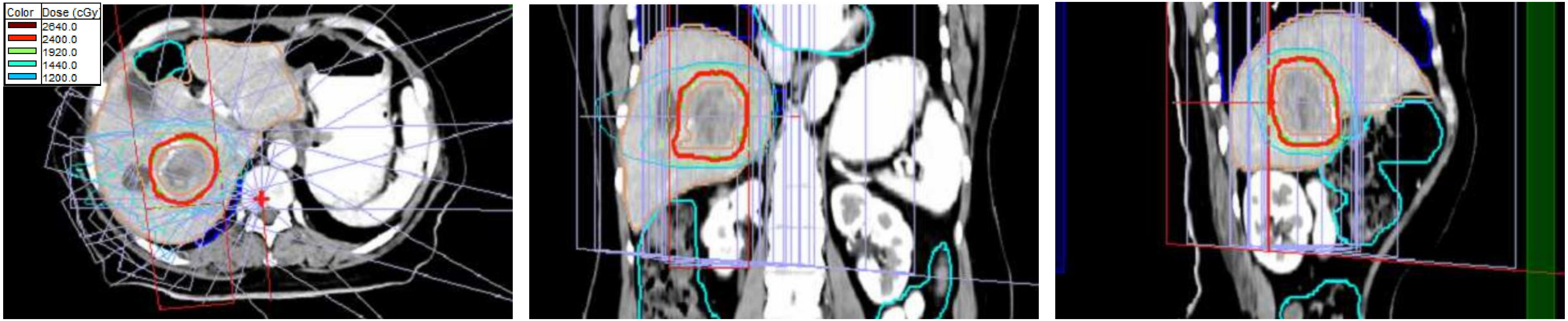

Patient.15

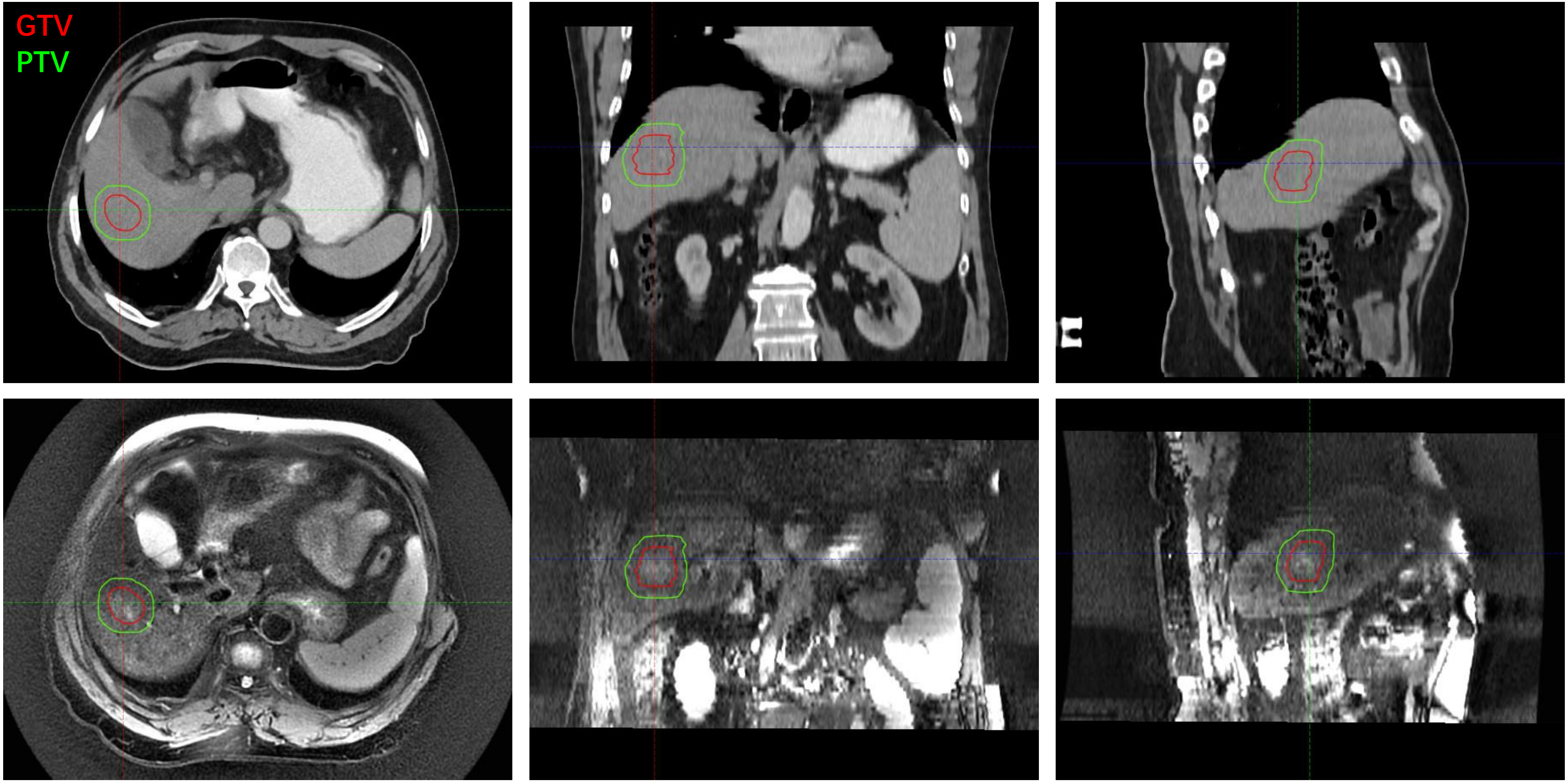

Patient.15

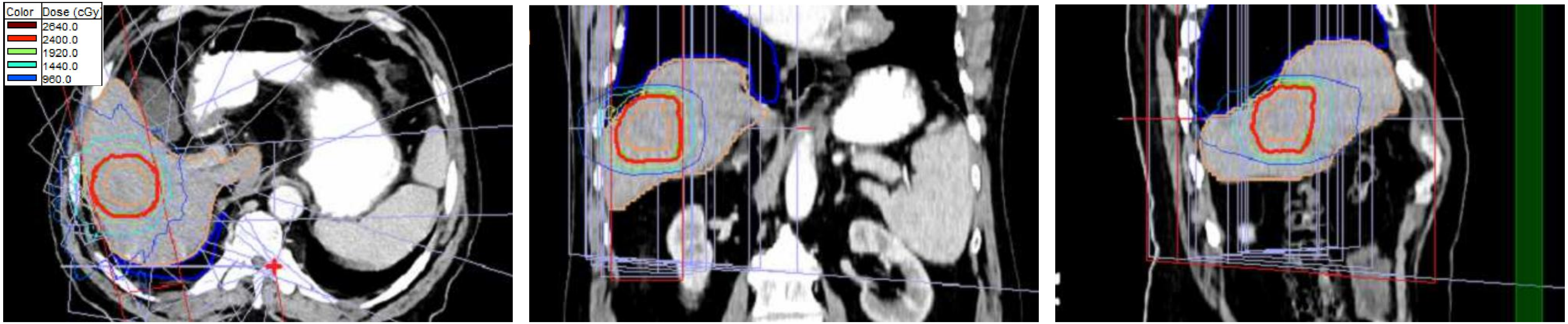

Patient.16

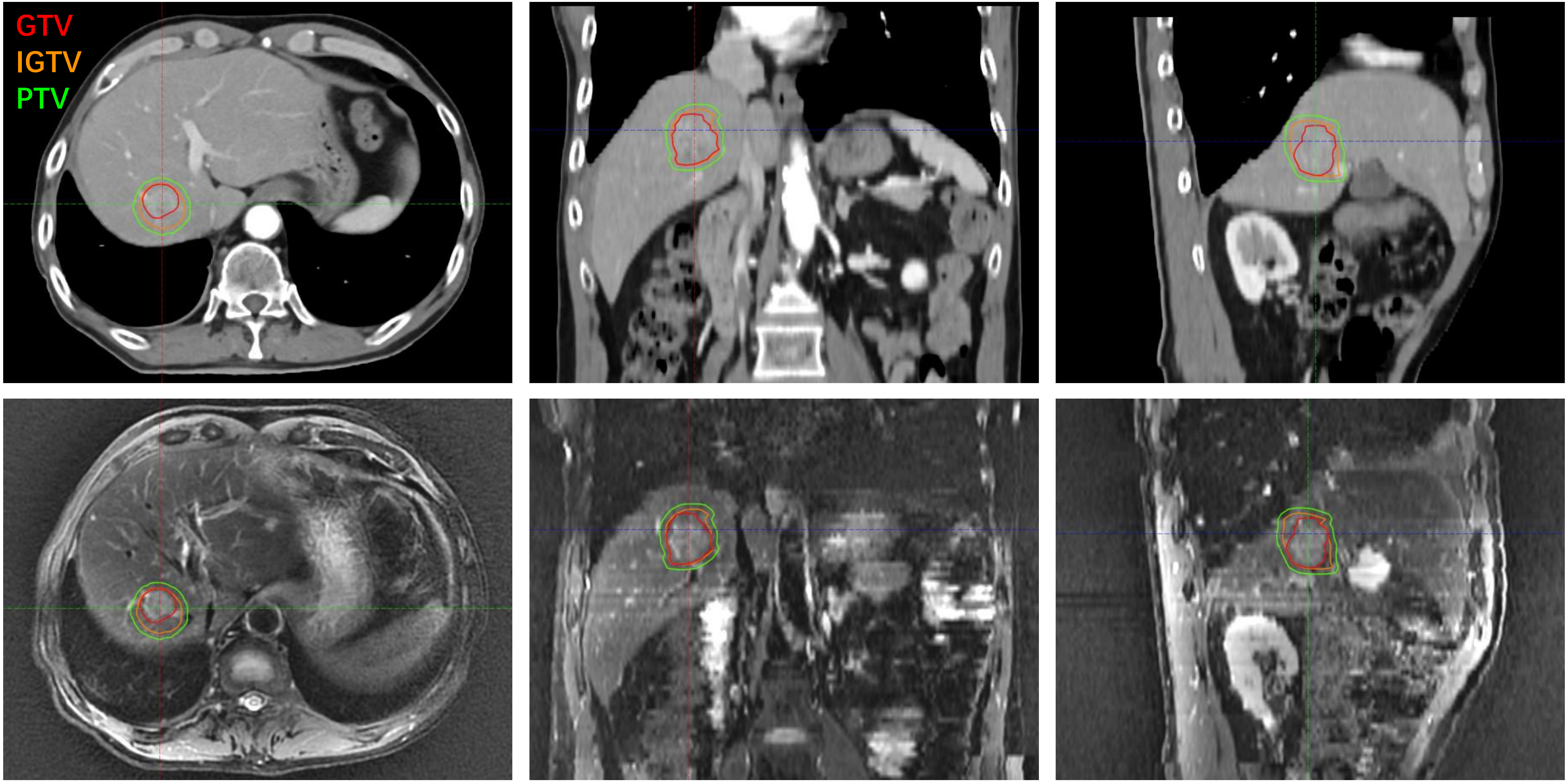

Patient.16

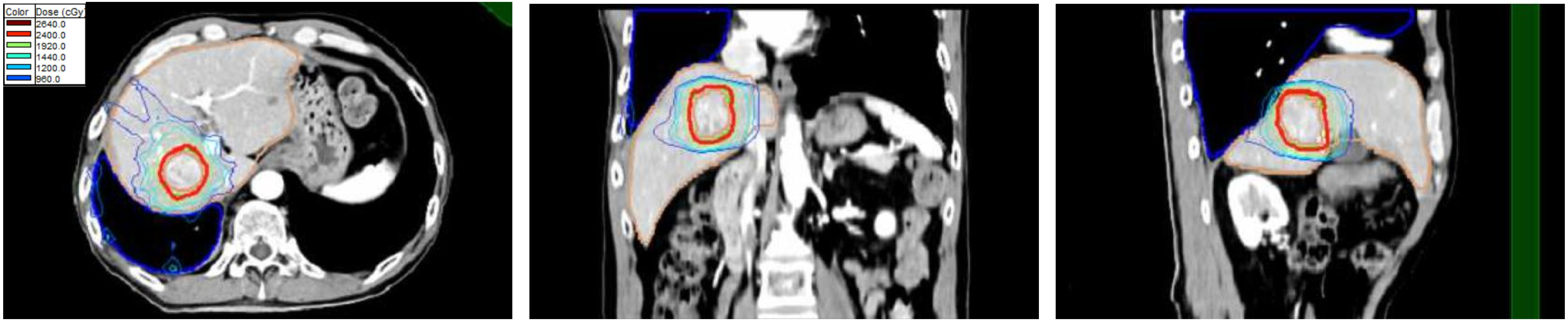

Patient.17

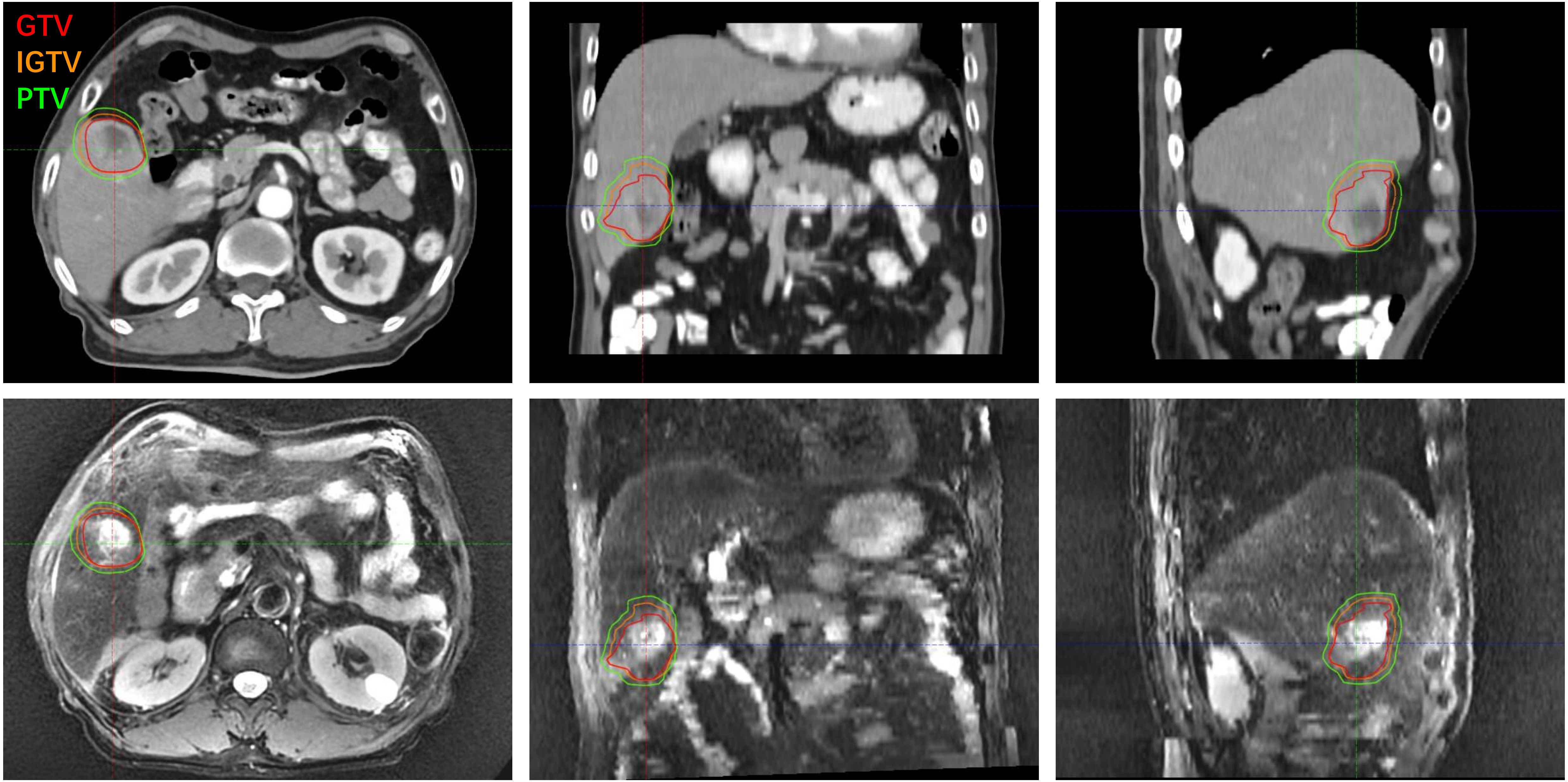

Patient.17

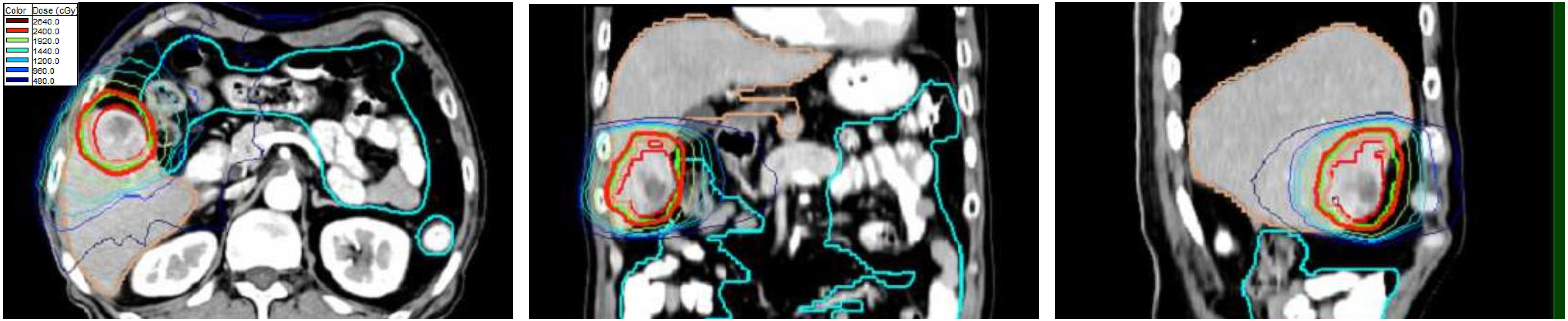

Patient.18

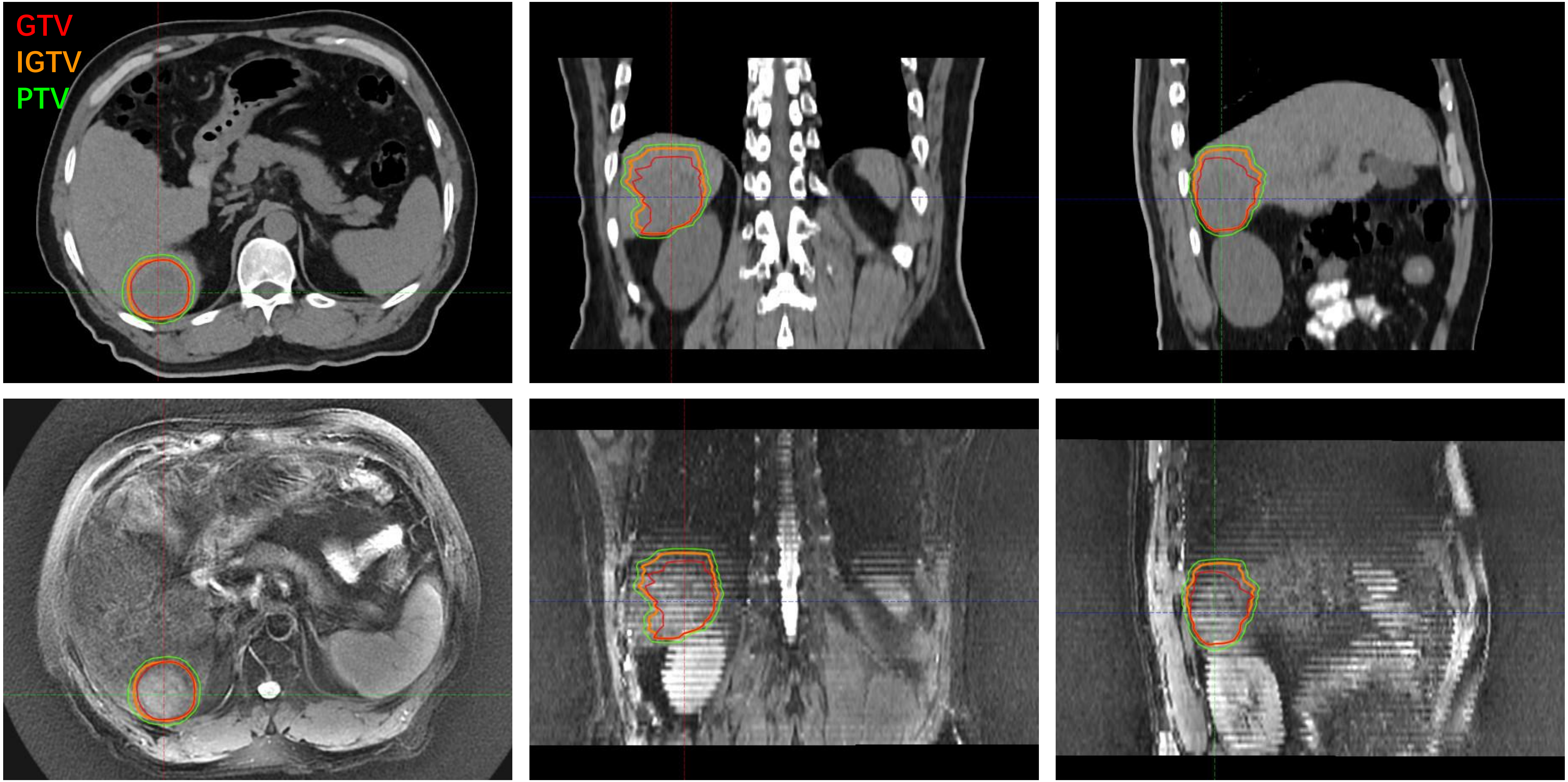

Patient.18

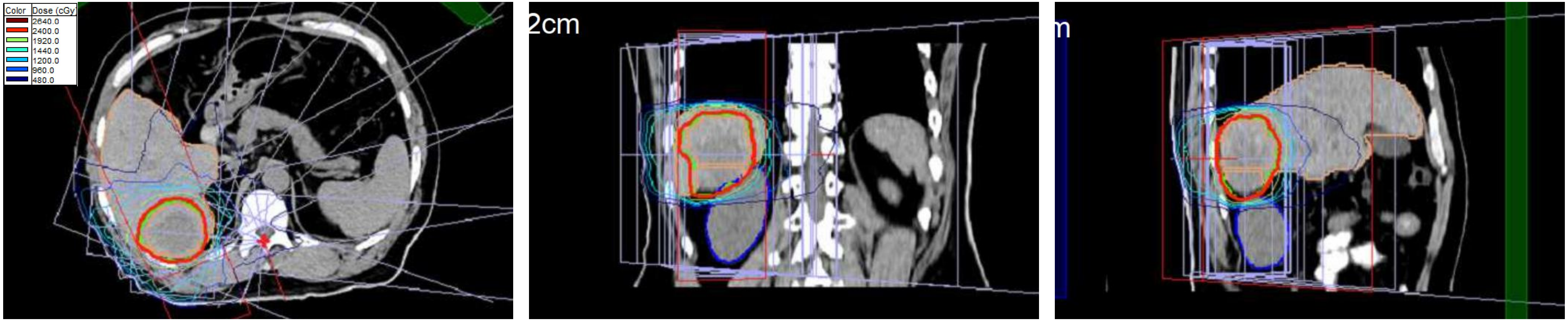

Patient.19

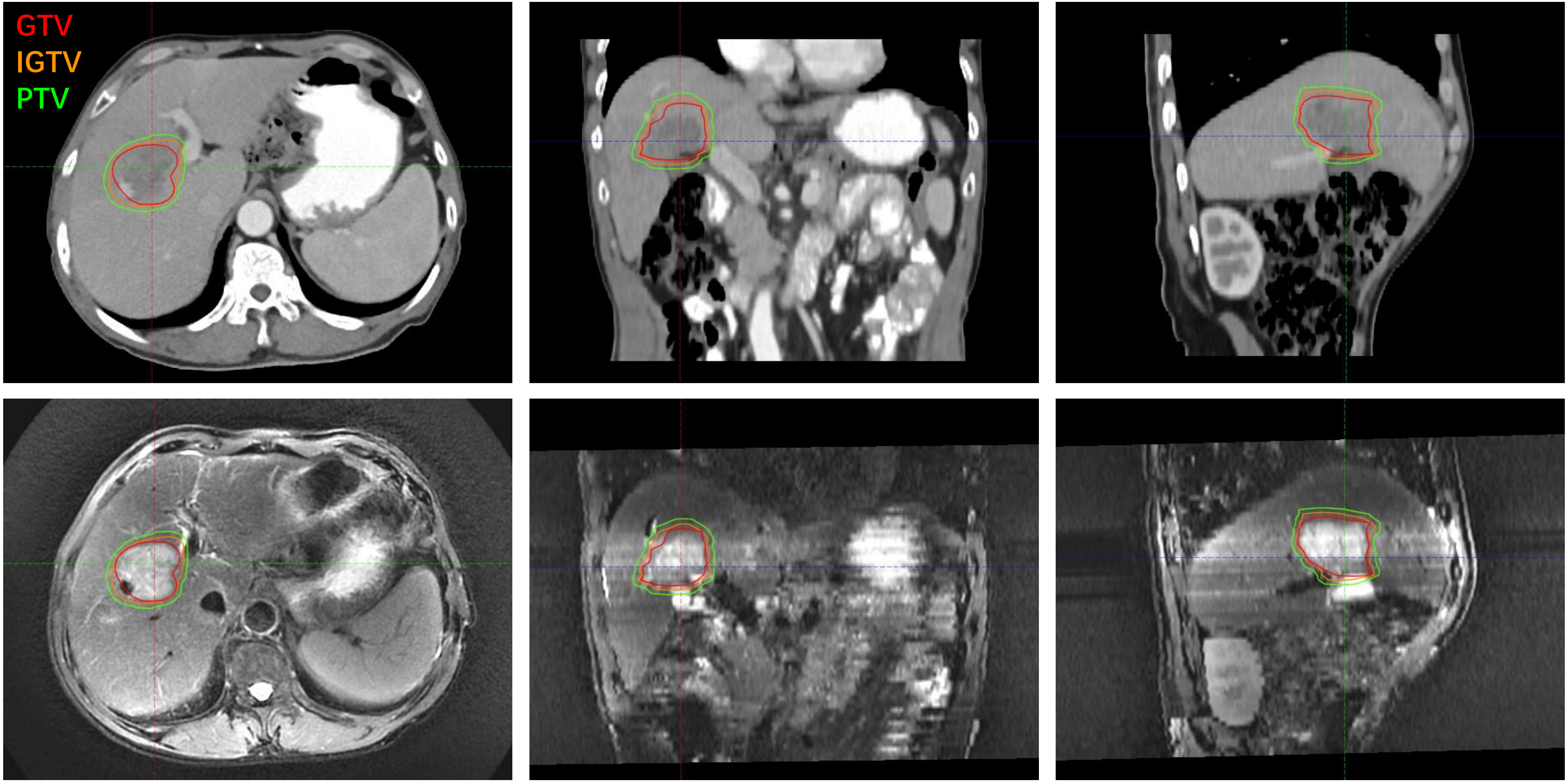

Patient.19

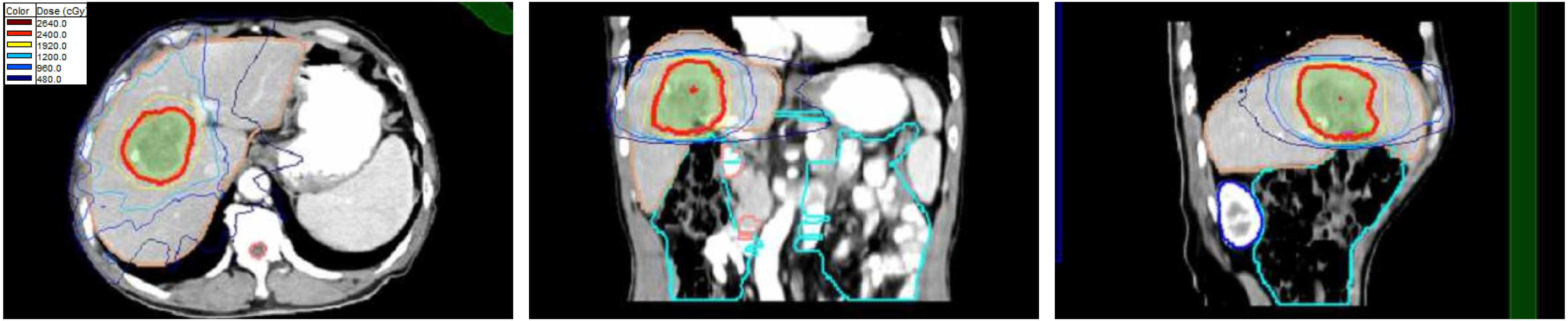

Patient.20

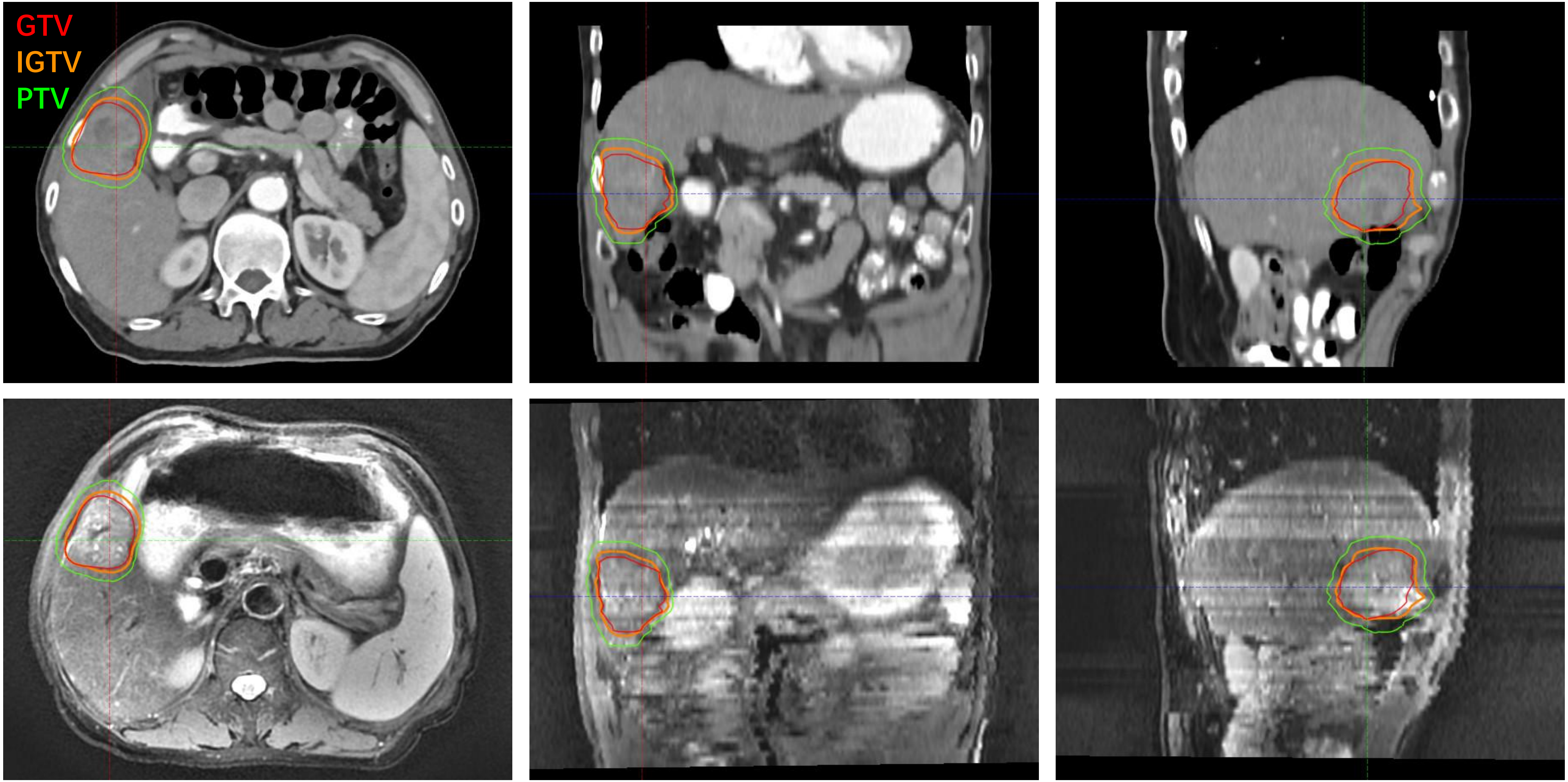

Patient.20

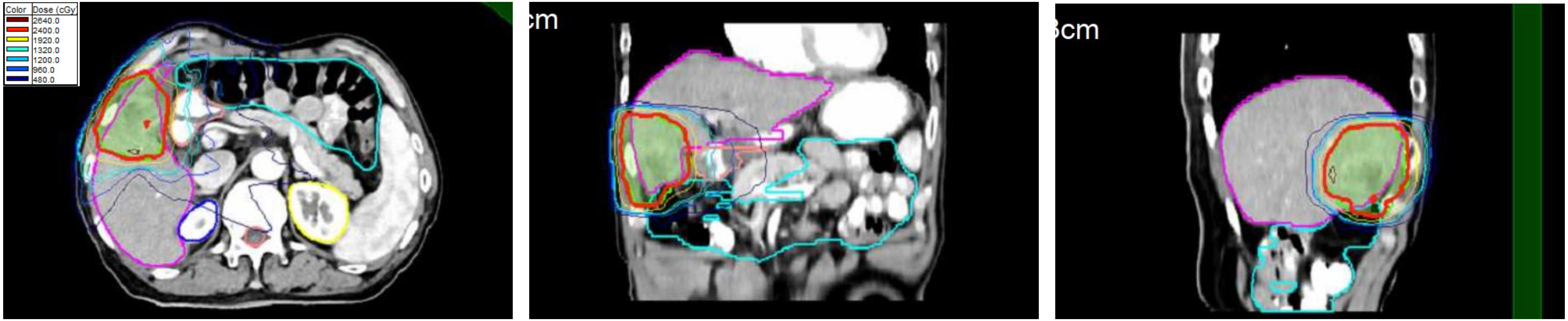

Supplement: Supplementary file 6 — Source Data [file 41467_2024_47420_MOESM6_ESM.zip › Source data_target delineations_plan evaluations.pdf]
